# Examining district-level disparity and determinants of timeliness of emergency medical services in Maharashtra, India

**DOI:** 10.1038/s41598-023-48713-1

**Published:** 2023-12-01

**Authors:** Arnab Jana, Ahana Sarkar, Vipul Parmar, Sujata Saunik

**Affiliations:** 1 Centre for Urban Science and Engineering, Indian Institute of Technology Bombay, Powai, Mumbai, Maharashtra India; 2 Indian Institute of Technology Bombay, Powai, Mumbai, Maharashtra India; 3 Administrative Reforms, Office and Management, Government of Maharashtra, Mumbai, India

**Keywords:** Health services, Health policy

## Abstract

The quality of emergency medical services remains a major public health issue in developing countries in terms of access, availability, or timely delivery, owing to high socio-economic and ethnic disparities. Particularly, the timeliness of EMS remains a drawback, leading to higher mortality and morbidity. The aim of the study is to assess the district-level differences and factors that influence ambulance travel time, as there was no study done in the Indian scenario. Sequential Explanatory Design was applied here, which involved a descriptive study and spatial analysis of the call volume and distribution to understand the operational challenges of MEMS, followed by in-depth interviews among medical officers and officials to explore the reasons for the challenges. The data, shared by the Department of Health, Government of Maharashtra, consisted of 38,823 records (emergency: 16,197 and hospital-to-hospital transfer: 22,626), including emergency and hospital-to-hospital transfer calls across 36 districts of Maharashtra for November 2022. Spatial analyses were performed to identify the districts with challenges of timeliness. The average ambulance response time (T) across the districts was reported at 134.5 min for emergency cases and 222.80 min for hospital-to-hospital transfer cases. The total ambulance response time, was classified as preparation time (t1:3.53 min for emergency, 3.69 min for hospital-to-hospital transfer), travel time from base to scene (t2: 23.15 min for emergency, 17.18 min for hospital-to-hospital transfer), time required at scene (t3: 12.12 min for emergency, 14.72 min for hospital-to-hospital transfer), travel time from scene to hospital (t4:39.41 min for emergency, 74.34 min for hospital-to-hospital transfer), patient handover time (t5: 10.85 min for emergency, 13.84 min for hospital-to-hospital transfer), and return from base to hospital (t6: 41.89 min for emergency, 94.72 min for hospital-to-hospital transfer). Multivariate linear regression was conducted to investigate the factors that influence ambulance travel time. The finding identifies that the ambulance travel time increased for the districts with lesser population density, lower road density, fewer hospitals, a higher district area served per ambulance, and a higher population served per ambulance. Additionally, socio-cultural reasons affecting health-seeking behaviour, early closing of healthcare centres, undercapacity and resource-deficit healthcare centres, and overloading of specialised tertiary hospitals were identified as determinants of delay in patient assessment and handover time in qualitative findings. A decisive and multi-sectoral approach is required to address the timeliness of EMS in the Indian context.

## Introduction

Emergency medical conditions can arise from the sudden onset or acute exacerbation of medical, surgical, or mental illnesses, such as cardiovascular diseases, trauma, sepsis, etc. ‘Emergency medical services (EMS)’ ensures the immediate treatment of such conditions through timely response and evaluation, provision of life-saving interventions, rapid transport, and transfer to the nearest operational healthcare facility^1^. These services, also called ‘Emergency Response Services’, aim to improve survival rates while controlling disability, stress, and morbidity levels. In the recent past, low- and middle-income countries have attempted to develop the EMS sector in terms of connectivity to sparse and distant rural transects, frequency of services, timely management of emergencies, and, more importantly, upgrading from a mere medical transport mode to the provision of arrival-to-stabilization treatment and prehospital care facilities.

Nevertheless, the quality of EMS remains a major public health issue in low- and middle-income countries in terms of access, availability, or timely delivery, mainly attributed to the high socio-economic and ethnic disparity. The ambulance response time emerges as a vital indicator for evaluating the efficiency of EMS in developing countries. Here, the rising population prompts concern among public managers regarding their ability to meet the demand for pre-hospital emergency care. EMS has recently operated through a single centralised telephonic contact in low-income countries such as Bangladesh, Cape Verde, Colombia, and the Dominican Republic^2^. A cross-country comparison revealed lower ambulance response times in Asia (7.3 min), Oceania (8 min), while higher in Ghana (19 min) and Brazil (21–27 min)^3^. Low-cost viable models for EMS, for instance, Rescue 1122 in Punjab province of Pakistan, with a GPS fleet tracking system, response time-induced service station positioning, certified trainers, etc., have turned out to be an effective system of pre-hospital care with an average response time of 7 min, even with bad road conditions, heavy traffic, and congested areas^4^. A study in South Africa revealed that the initial incremental addition of vehicles led to an improvement in response time^5^.

The Republic of India, the world’s seventh-largest landmass and second-most populous country with 28 states and 8 union territories, lacks a centralized body for EMS operations. Fragmented service, sparse accessibility, wide variability in their dispatch and transport capabilities, inconsistency in personnel training standards, and a lack of awareness and knowledge regarding Dial ambulances are major drawbacks in the prehospital emergency care sector^6^. As per the report published by the Indian Council of Medical Research (ICMR) in 2022, on reviewing the available prehospital services in the Indian subcontinent, the unmet needs were still reported to be high, with ‘EMS being fragmented, non-accessible throughout the country, and where available, underutilised, with only 43% of emergencies reaching hospitals via this mechanism’^7^. Severe health access inequality in India has witnessed situations where only 0.5% of head injury cases are transported by ambulances^8^, only 7% of head injury cases reach hospitals within the ‘first golden hour’, only 12% of stroke patients avail ambulances for transportation in an urban area^9^, and 80% of trauma patients fail to avail medical care access within the first hour^10^. Lastly, 50% of emergency cases receive prehospital treatment from non-qualified personnel^11^.

In most Indian states, ‘108 Ambulance Service’ is available, yet it often functions primarily as a transportation system. Maharashtra, a state in the western peninsular region of India and the second-most populous state in the country, launched a comprehensive EMS scheme in 2014. In this research, the operational schema of the implemented Maharashtra EMS (MEMS) has been put forward, along with the operational details and timeliness of the service. The study aims to assess the MEMS call details and volume while highlighting the district-level differences in emergency response services and inter-facility transfers as well as the factors influencing the response time in Maharashtra.

This research highlights the following: (i) it presents the operational framework of the MEMS pre-hospital care service delivery and the healthcare referral system across the state, and (ii) investigates the planning and infrastructure-related factors along with context-specific socio-cultural practices that influence ambulance response time and overall episode time. Furthermore, it identifies the current challenges related to service timeliness in different districts of Maharashtra.

## Methods

### About Maharashtra Emergency Medical Services

MEMS is a publicly funded scheme, offering free-of-cost service, by the Government of Maharashtra under the National Health Mission (NHM), and is operated via a public–private partnership model. MEMS functions with the help of the state-of-the-art Emergency Response Centre (ERC), operated from Pune. ERC operates 24 × 7 through a state-wise, centrally operated toll-free telephone number, 108, which can be dialled from any part of the state. MEMS aims to provide 24 × 7 prehospital EMS across Maharashtra, including disaster situations in the state, to achieve a 20% reduction in mortality and morbidity. To date, MEMS has served more than 7,583,904 patients since its launch in 2014 until July 31st, 2022, with 57.4% of calls coming from generic medical emergency cases, followed by 18.20% from pregnancy and labour cases (https://nrhm.maharashtra.gov.in/ems.html).

The service is integrated with appropriate technology, such as a voice logger system, GIS (Geographic Information System), GPS (Geographic Position System), AVLT (Automatic Vehicle Location System), and Mobile Communication System (MCS). The ambulances offered in this service are equipped with an ambulance cot, scoop stretcher, bi-phase defibrillator, cardiac monitor with recorder (for ALS only), transport ventilator (for ALS only), pulse oximeter (for BLS only), suction pump (manual and electronic), oxygen cylinder, and a disposable pregnancy kit. These ambulances are operated by trained Emergency Medical Service Officers (EMSOs).

### Study design and setting

Maharashtra has 36 districts; the demographic details are highlighted in Appendix I. The districts of Mumbai and Thane have a population of over 10 million, whereas Pune has over 9 million, and Nashik has more than 6 million as per the 2011 census. Nandurbar and Gadchiroli are the two major districts, with more than 50% of the population belonging to Scheduled Caste (SC) and Scheduled Tribe (ST), the nationally recognized historically marginalized and disadvantaged communities of India^12^.

### Methods

#### Study design

The mixed-methods approach, namely, Sequential Explanatory Design was adopted in this study, which involved a set of sequential quantitative and qualitative analyses. A descriptive study and district-wise spatial analysis of the call volume and MEMS ambulance episode time were followed by in-depth interviews among the MEMS medical officers and state government officials to explore the reasons behind the operational discrepancies in MEMS^13^. The theoretical underpinning of the qualitative component was the constructivist paradigm and the researchers’ plan to describe the various reasons for the MEMS delivery challenges. While the quantitative analysis-derived associations established the determinants, the qualitative study’s methodological emphasis was phenomenology, and in-depth interviews were undertaken to explore the phenomenon experienced by the medical officers and programme functionaries. The quantitative data was analysed first, followed by the collection and analysis of qualitative data. The researchers first attempted to establish quantitative relationships regarding determinants of ambulance response time and then delve deeper into the qualitative aspects to validate the quantitative findings.

#### Sampling, recruitment and data collection.

*Quantitative.* The quantitative data consists of all the service delivery records of 38,823 calls for November 2022. Using this data, we contrasted hospital-to-hospital transfers (22,626) and emergency calls (16,197). This study encompasses a secondary data analysis of single-month call details, including information such as call timing, initial patient condition, patient location, ambulance response time in travel, patient handling, and hospital transfer.

*Qualitative.* The interview guide was designed to elicit perspectives on the reasons behind the operational challenges of MEMS. A set of follow-up qualitative semi-structured interviews were conducted with medical officers from the MEMS control centre and officials from the Department of Health, Government of Maharashtra, working with 108 emergency services in Maharashtra. The in-depth interviews were conducted both offline and online at a time and place convenient for the medical officers as well as the officials. To align with the inquisitive approach of the qualitative research strategy, a combination of purposive sampling and snowball sampling techniques was utilized to engage with the participant group. The details of the qualitative analysis-related method are explained in Supplement 1.

### Data sources

The public call records or calls from the ‘Call 108 MH’ app^14^ are stored and archived by the Department of Health, Government of Maharashtra. The details of the call and patient records, ambulance details, and the episode of prehospital care are maintained through manual records as well as digitally using voice logger systems, GIS, GPS trackers, and MCS. The data for November 2022 was shared by the respective department.

### Details of the data

The study involved two types of call data, which are as follows:

- Emergency service: In the case of emergency service, the control center initiates the process to verify the caller to assess the need and pinpoint the accurate location.
- Hospital-to-hospital service: The concerned medical personnel at the hospital initiates the call, and as per the need for specialised care, the destination health center is identified, and the service is provided to the concerned patient.

In either emergency or hospital-to-hospital services, the hospital officials or the patient (or the family members or anyone in the vicinity) call service 108, free of charge. This data consists of both call details along with the time of the call, duration of the call, the time required by the ambulance to reach the scene from the base station, the time taken by the ambulance to transfer the concerned patient to the ambulance, and the overall time required by the ambulance to reach the destination health care facilities. The medical personnel assess the patients at the scene and record the base-line health condition of the patient based on the Glasgow Coma Scale (GCS), recording three aspects of responsiveness: eye-opening, motor, and verbal responses, along with other information such as age, gender, impression, and severity of disease before and after the transfer to the concerned healthcare service.

**Figure 1:**
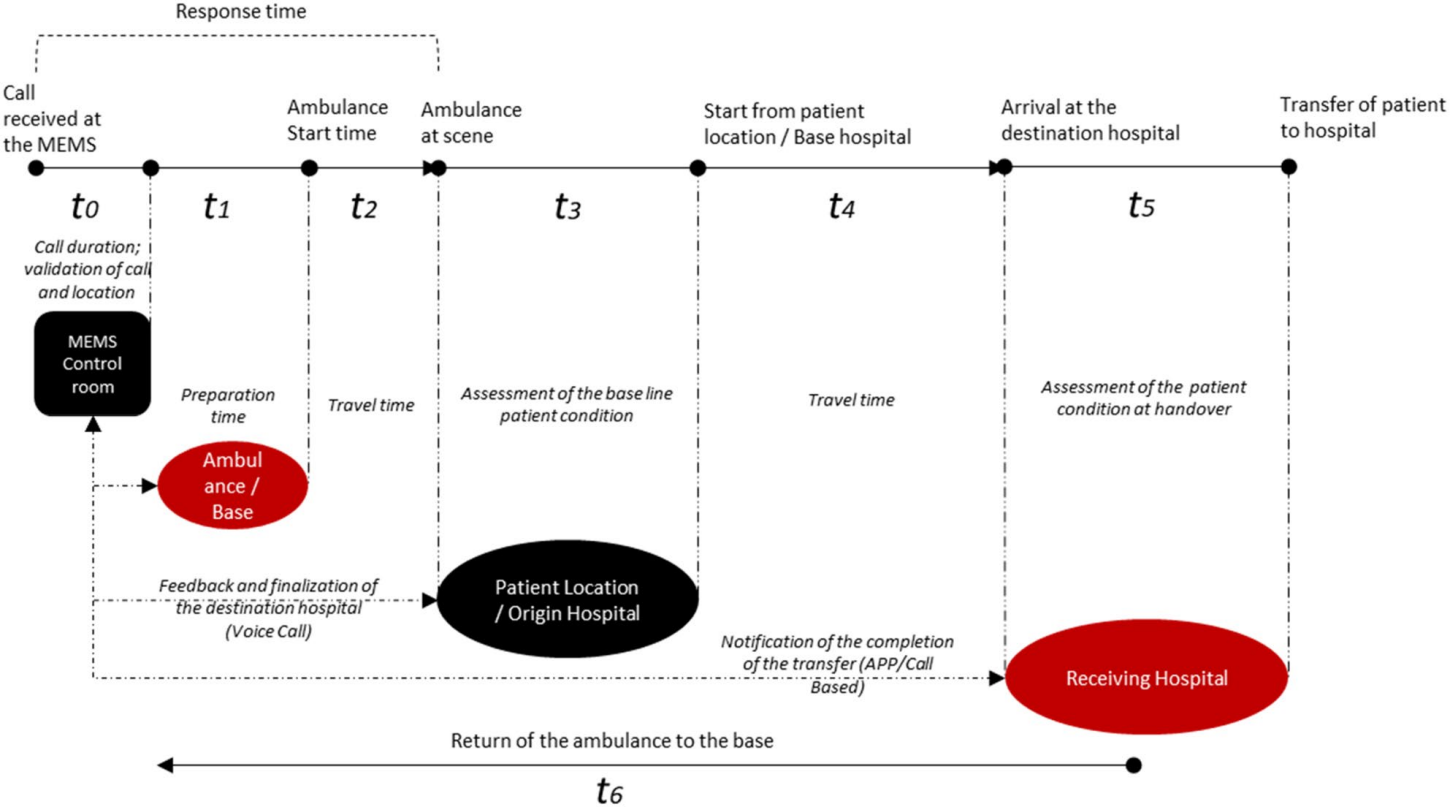
Conceptual framework demonstrating the operational framework and complete episode of patient transfer by MEMS (108 Ambulance service) (Source: Authors’ compilation).

Based on the initial assessment, the control centre initiates the process of dispatching the ambulance, which begins with the search for the nearest available ambulance. The search is based on the live location of the ambulances and depends on the estimated time of arrival (ETA) of the ambulance at the scene. It adopts an incremental increase in search time in cases of unavailability until an ambulance is identified and dispatched. The complete episode of the patient transfer through the MEMS is shown in Fig. 1. The total episode has been subdivided into six components, as shown in Eqs. (1 and 2), and the assessment of the MEMS has been done to improve these six segments.

1 $$ {\text{Total episode time }}\left( {\text{T}} \right) \, = {\text{ t1 }} + {\text{ t2 }} + {\text{ t3 }} + {\text{ t4 }} + {\text{ t5}} $$

2 $$ {\text{Total ambulance base to base time T}}_{{{\text{base}}}} = {\text{ T }} + {\text{ t6}} $$

where, t1 = Time taken to prepare and start the ambulance service from the base after receiving the call. t2 = Time taken by MEMS 108 ambulance service to reach the scene from base. t3 = Patient assessment time required at the scene. t4 = Time required to travel from patient location to reach the hospital. t5 = Patient handover time at the hospital. t6 = Time required by the service to return to the base from the hospital.

The NHM recommends that the ambulance response time for reaching the patient should be within 30 min for rural areas and 20 min for urban areas after receiving the call, and the patient should reach the health facility within the next 30 min as a quality benchmark^7^.

### Operational definition

Different operational definitions were considered in this study for spatial and statistical analyses.

In order to understand the prominent health issues, present in a district, the districts recording more than 150 calls and 250 calls of any particular disease, such as trauma or injury, abdominal pain or problem, respiratory discomfort, head injury, weakness, poisoning, or chest pain, in that month were considered to be prominent health issues for emergency and hospital-to-hospital transfer cases, respectively.

For calculating the six components of MEMS ambulance episode time (t1–t6) for both emergency and hospital-to-hospital transfer calls, time (in minutes) recorded equal to 0 and more than 360 min (6 h) were removed from the dataset for calculating the average values (details in Appendix I).

The statistical analyses performed in this study considered the following indicators: population density, road density, the area covered per unit ambulance, and whether one ambulance is serving more than 100,000 people. While the data concerning the ‘Total number of hospitals per district’ were directly obtained from the MEMS report, the other variables were formulated in this study for the 36 districts of Maharashtra.

‘Population density', population per unit land area, is defined as the total number of populations residing per sq. km of district area.

In order to understand the road network and connectivity across districts, the indicator of road density was calculated. ‘Road density', in this study, is calculated as the total length of road (in km) per 100 km^2^ of district area.

Third, to investigate the MEMS health infrastructure quantity and availability in the districts, this study formulated the indicator of ‘Area covered per unit ambulance’, which determines the total area of the district in sq. km that is served by one ambulance. It is calculated as the total district area divided by the total number of ambulances allocated for that particular district.

The last indicator used in this study measured the number of people in the district served by a one-unit ambulance. As per World Health Organisation (WHO) standards, at least one ambulance should serve 100,000 people in plain areas and 70,000 in distant, tribal areas or hilly terrain. Using the WHO standard, this study identified whether ‘one ambulance was serving more than 100,000 people in a district’.

### Data management and analysis

The data was analysed in two stages. The first stage involved the district-level spatial analysis of socio-demographic characteristics, emergency and hospital-to-hospital transfer calls, and MEMS episode times to recognize the district-level variations in MEMS episode time. Second, a series of correlations and statistical analyses were conducted to investigate the role of infrastructure on MEMS episode time.

First, the data was cleaned in Python^15^. QGIS^16^ and Python were utilised to analyse and map the call pattern and episode timing for all districts. Similar approaches have been employed in recent research that used Python to develop the framework and QGIS to visualize and amalgamate all the different data^17^. A weighted overlay analysis was performed to explore the district-wise distribution of prominent health issues among males and females. The methodological details of all spatial analyses are provided in Appendix II.

Secondly, the post-cleaning data was further analysed to identify the factors influencing ambulance response time and overall episode time, along with their variations and delays. Multivariate linear regression was employed to identify the factors influencing ambulance response time and episode time. Variables with p-value < 0.25 in the bivariate analysis and other critical infrastructural determinants of episode time were considered for multivariate analysis. Variables with p-value < 0.1 were declared significant, while variables with p-value < 0.05 and 0.001 were highlighted accordingly^18^. Adjusted R^2^ values were reported for individual models as a measure of goodness of fit.

To better understand MEMS across the districts, several rounds of stakeholder discussions were conducted with officials from the Department of Health, Government of Maharashtra, and health officials and doctors from ERC.

### Ethical consideration

The quantitative data was provided by the Department of Health, Government of Maharashtra, with due approval from the concerned authorities. The data was not collected by the authors. The data shared for this study does not contain any personal identifiers or fields that reveal the identity of the patient or caller (family member, acquaintances, friends/Good Samaritan). All authors consent to the publication of this manuscript.

The study documents were reviewed by the Institutional Review Board (IRB) of the Indian Institute of Technology, Bombay. The committee approved the ethical conduct of the study. The committee issued a declaration of no objection confirming that the project fulfils the scientific and ethical standards for research.

## Results

### Socio-demographic characteristics and Emergency and Hospital-to-hospital transfer call details in different districts of Maharashtra, India

**Figure 2:**
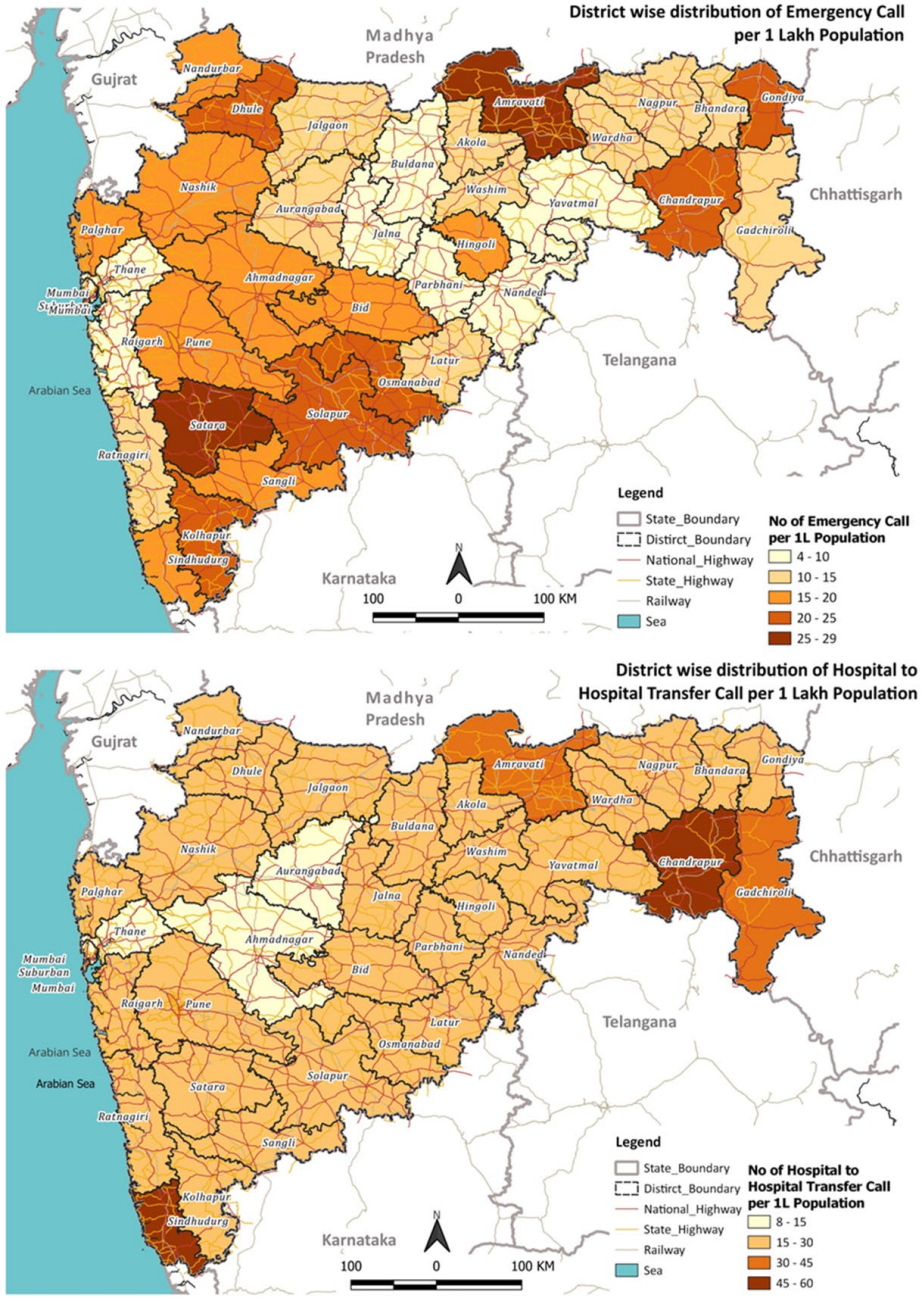
Distribution of Emergency and Hospital-to-Hospital transfer calls in the different districts of Maharashtra. 1 Lakh = 100,000. QGIS Geographic Information System v3.28.3-Firenze. QGIS.org, 2023. QGIS Association. http://www.qgis.org.

The analyses revealed that the district-wise highest number of emergency calls were recorded in the districts of Amravati (29) and Satara (29), while the district average was 15 (refer to Fig. 2). Chandrapur and Sindhudurg also reported the highest hospital-to-hospital transfer calls (49 and 60, respectively) per 100,000 population, exceeding the district average (23).

The districts in eastern Maharashtra, such as Chandrapur and Amravati, have relatively lower populations but constitute 32% and 33% of the Scheduled Caste/Scheduled Tribe (SC/ST) population, respectively, with a lower district domestic product. Nonetheless, they reported noteworthy call volumes, likely due to the affordability of the MEMS in these areas.

**Figure 3:**
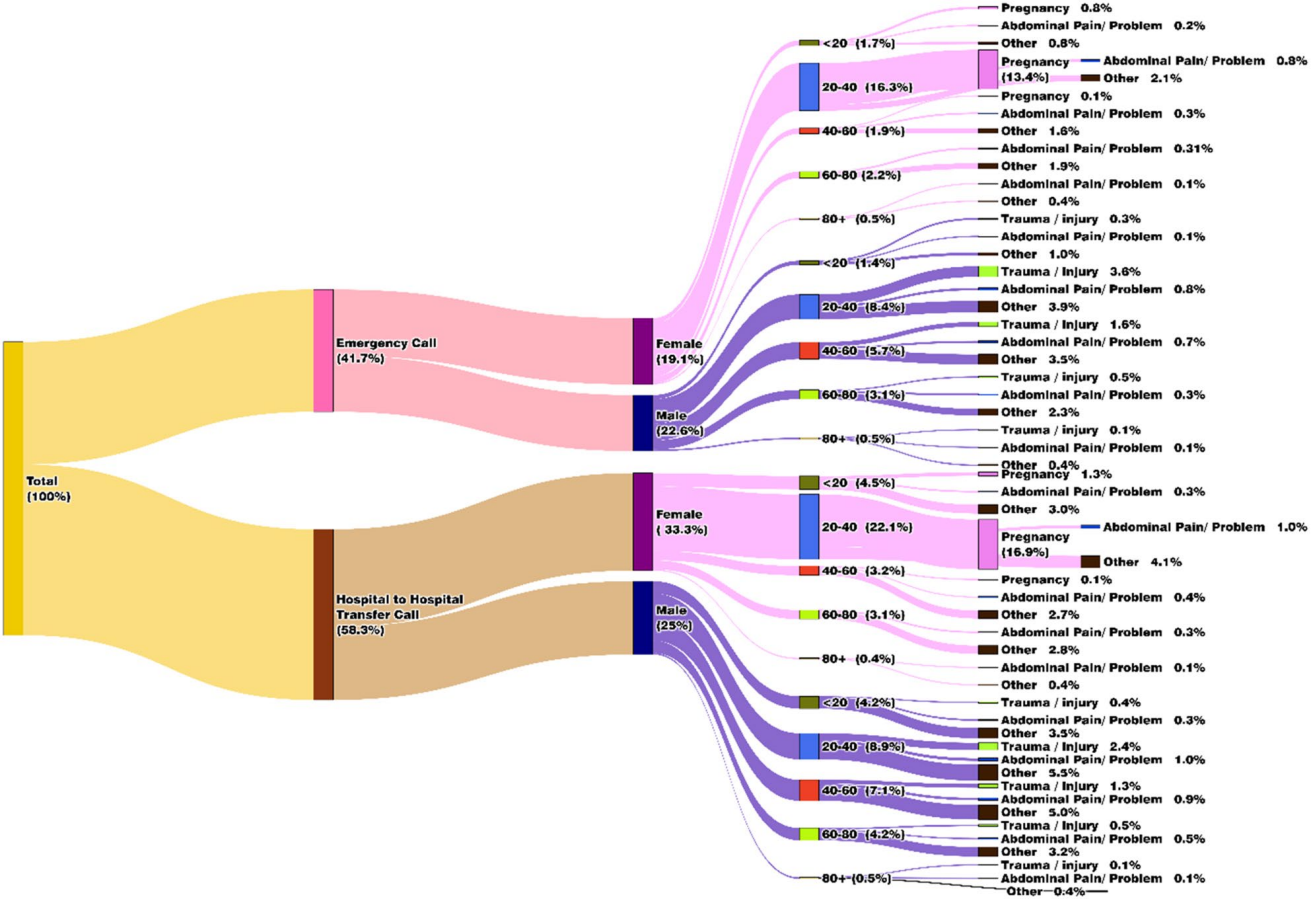
Sankey diagram demonstrating the socio-economic distribution of patients’ calls for MEMS 108 Ambulance services (Visualisation is created using python^13^).

Figure 3 explains that out of 38,823 MEMS calls in November 2022, emergency calls accounted for 41.7%, whereas the rest, 58.3%, were for hospital-to-hospital transfer calls. The share of calls recorded from females was 19.1% for emergencies and 33.3% for hospital-to-hospital transfer cases, and for males, it was 22.6% for emergencies and 25% for hospital-to-hospital transfer cases. This phenomenon can be attributed to reasons such as culture and societal norms-driven differences in health-seeking behaviour between genders, variations in healthcare needs and utilization often leading to higher hospital-to-hospital transfers for women due to reproductive health or maternity-related issues^19^, public awareness and health education differences between genders, and lastly, varying population distribution (52% male and 48% female in Maharashtra).

The observation that both males and females belonging to the age group 20–40 years (emergency: 16.3% females and 8.4% males; hospital-to-hospital transfer: 22.1% females and 8.9% males) availed of the service more than the other age cohorts can be attributed to several factors. This age group includes young male workers who engage in travel-related activities and females belonging to the reproductive cohort. This entails that while males may be exposed to a higher risk of injuries, accidents, or health emergencies, women experience reproductive health issues related to pregnancy and childbirth^19,20^. While pregnancy recorded the highest share among females (13.4% for emergency and 16.9% for hospital-to-hospital transfer), a higher proportion of males availed of MEMS for trauma, injury, and abdominal pain.

**Figure 4:**
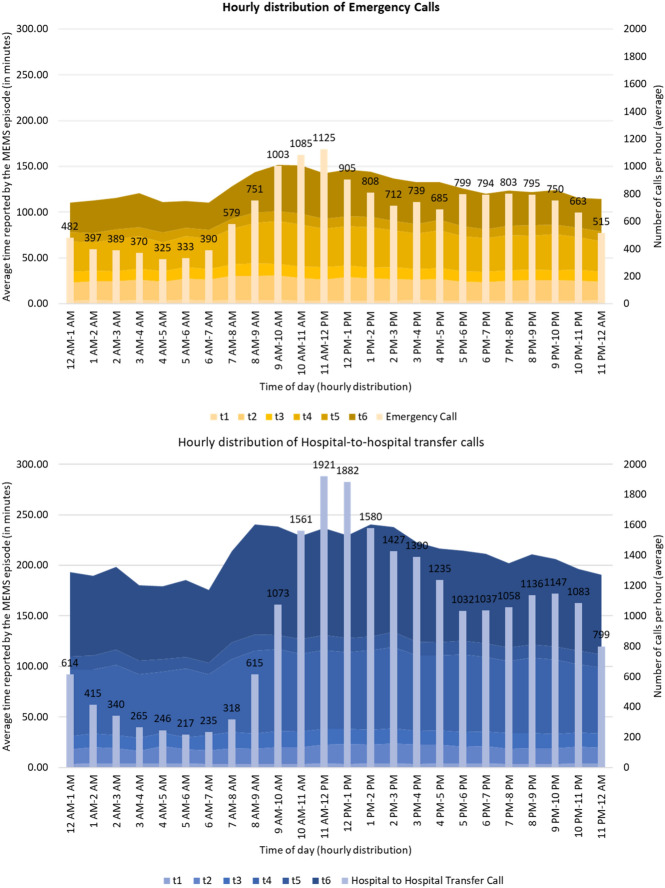
Hourly calls and episode time distribution for emergency and hospital-to-hospital transfer cases (Authors’ computation).

**Figure 5:**
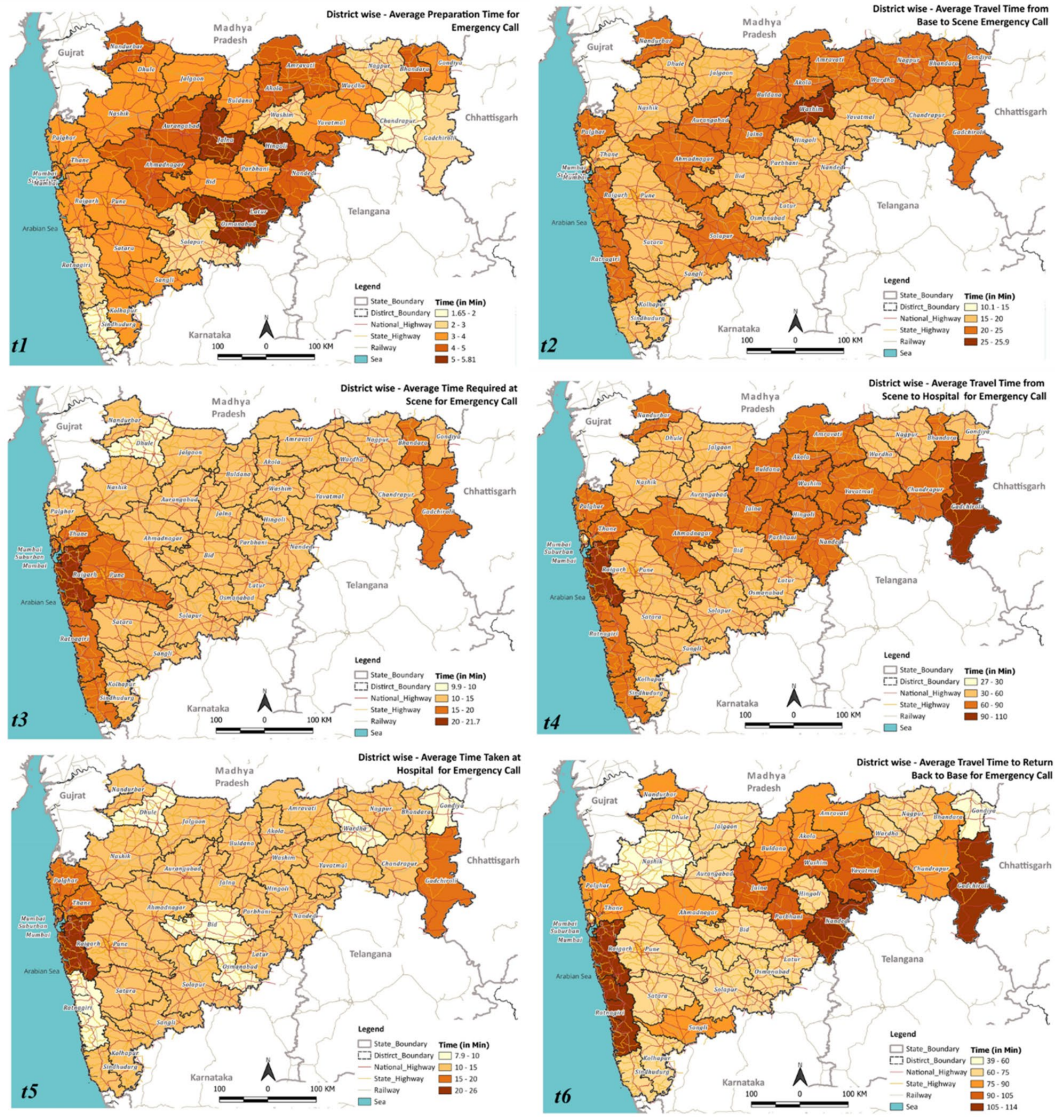
Total response time for emergency call responses in the different districts of Maharashtra. QGIS Geographic Information System v3.28.3-Firenze. QGIS.org, 2023. QGIS Association. http://www.qgis.org.

The weighted overlay analysis of the district-wise distribution of prominent health issues among males and females highlighted that the disease prominence for emergency calls was high in Amravati, Gondiya, Satara, Sangli, Palghar, and Sindhudurg for males and Amravati, Satara, and Kolhapur for females. For hospital-to-hospital transfer cases, Sindhudurg, Chandrapur, and Amravati had the maximum number of prominent male health issues; Sindhudurg and Chandrapur had the leading female health issues (details in Appendix III: refer to Figs. 4, 5, 8, and 9).

### MEMS episode in Maharashtra

The average hourly variation of the volume of calls and distribution of MEMS episode time for emergency and hospital-to-hospital services is shown in Fig. 4. The volume of hospital-to-hospital calls was found to be higher than that of emergency cases, and the volume increased with the opening hour of health facilities (i.e., 9 a.m. onwards). As expected, given the state’s health infrastructure hierarchy, compared to emergency call-related ambulance travel time, the time for the ambulance to reach the scene from the base and from the scene to the hospital was higher for hospital-to-hospital transfer cases.

**Figure 6:**
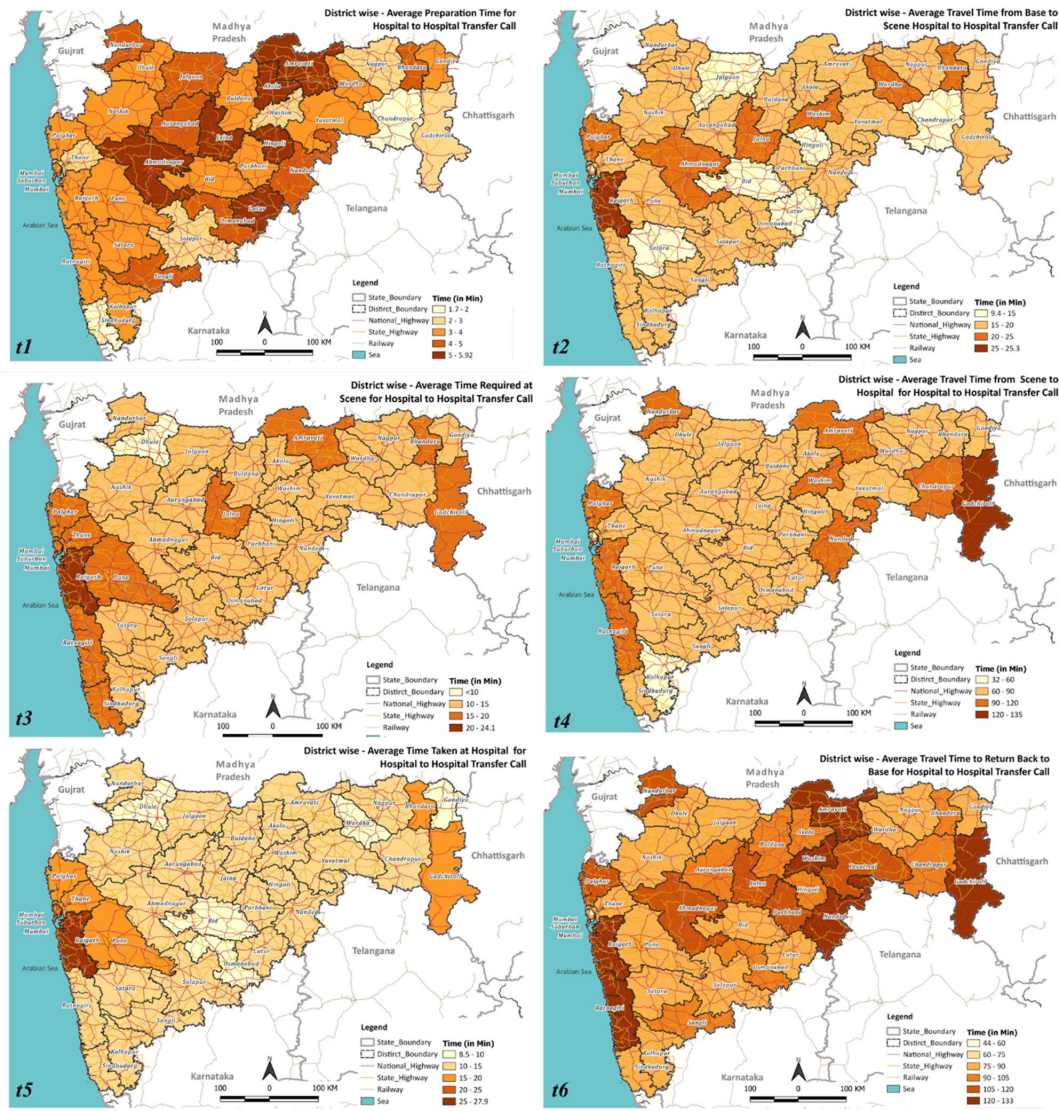
Total response time for hospital-to-hospital transfer call responses in the different districts of Maharashtra. QGIS Geographic Information System v3.28.3-Firenze. QGIS.org, 2023. QGIS Association. http://www.qgis.org.

The district-wise performance with respect to the episode duration for emergency calls and hospital-to-hospital transfer calls is shown in Figs. 5 and 6, respectively. On average, the overall episode time varies between 130 and 210 min for most of the districts, with Raigad having an average response time of 278 min, much exceeding the district average of 134.5 min. Appendix I, demonstrating the variation of t1 to t6, highlighted that the average time taken was t1 = 3.53 min, t2 = 23.15 min, t3 = 12.12 min, t4 = 39.41 min, t5 = 10.85 min, and t6 = 41.89 min. The average travel time for the ambulance to reach the patient location (t2) was found to be above 15 min across all districts in Maharashtra, with a few exceeding 25 min. The average scene time (t3) has been less than 15 min for 29 out of 36 districts. For the travel time from scene to hospital (t4), 13 out of 36 districts averaged more than an hour, with Gadchiroli and Raigad exceeding 90 min. For the average patient handover time (t5), 24 out of 36 districts exceeded 10 min, with Raigad reporting around 30 min. When investigated at individual events, Raigad was observed to require more time at the scene (t3: 21 min > average 12.12 min), to travel from the scene to the hospital (t4: 77 min > average 39.41 min), handover time at the hospital (t5: 26–30 min > average 10.85 min), and lastly, to return to base from the hospital (t6: 105 min > average 41.89 min).

The observed variation of t1 to t6 for hospital-to-hospital transfer calls for different districts of Maharashtra highlighted that the average time taken was t1 = 3.69 min, t2 = 17.18 min, t3 = 14.72 min, t4 = 74.34 min, t5 = 13.84 min, and t6 = 94.72 min (refer to Appendix I). Figure 6 demonstrated that most districts’ total episode time (T) varies between 184 and 233 min (average: 222.80 min). However, Raigad has an average response time of 282 min. While seven districts took more than 20 min to reach the scene from the base, with Raigad taking the maximum time (25 min), all the districts exceeded one hour to reach a hospital from the scene, with Gadchiroli and Raigad taking more than 100 min. Patient handover time at the hospital was higher in Mumbai (23 min) and Raigad (27 min). Notably, in this case, Raigad required more time at the scene (t3: 24 min > average 14.72 min), handover time at the hospital (t5: 26–28 min > average 13.84 min), and lastly, to return to base from the hospital (t6: 111–130 min > 94.72 min).

The causes of the variations are distinct for various districts; nevertheless, a few plausible reasons are given below. The delay in each event may be attributed to the following reasons:

- *t1*—Unavailability of the required ambulances and healthcare professionals at the base.
- *t2*—poor road infrastructure and lack of connectivity of the base station to the scene.
- *t3*—(i) assessment of the patient; (ii) intrinsic characteristics of the patient.
- *t4*—poor road infrastructure and lack of connectivity of the scene to the destination hospital.
- *t5*—Availability of infrastructure at the hospital; availability of required human resources and medical personnel.

### Determinants of ambulance response time in transportation

#### Correlation analysis

**Figure 7:**
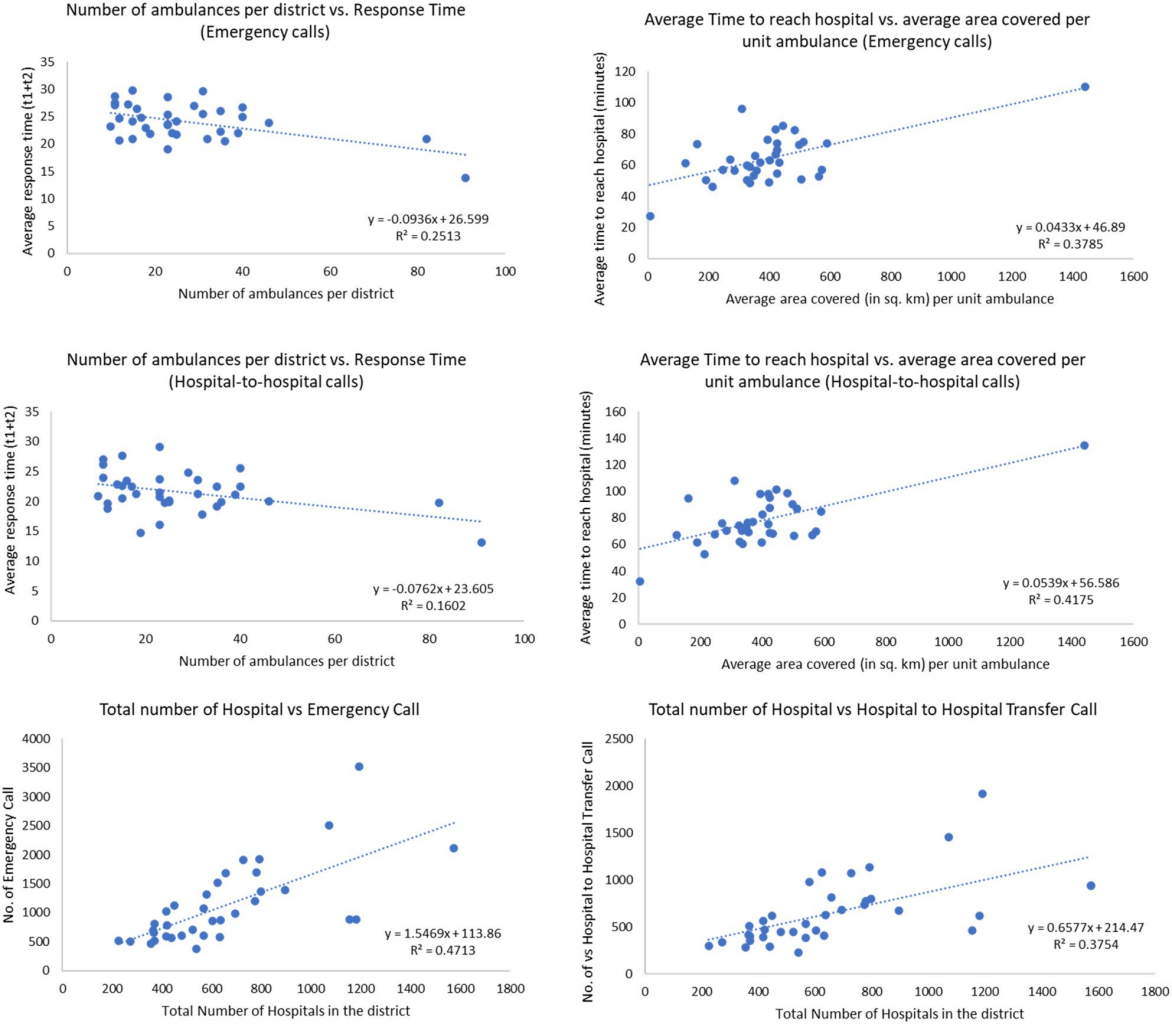
Correlations analyses of number of calls and average response time with respect to spatial characteristics representing demography and infrastructure (Authors’ computation).

Figure 7 demonstrates a negative correlation between the number of ambulances available per district and response time (t1 + t2), indicating that the districts with fewer MEMS ambulances expressed higher response time in serving the patients during emergencies and hospital-to-hospital transfers after receiving the calls. The unavailability of adequate ambulances at base or in close proximity to the scene increases the allocation time for the search algorithm, thereby delaying the overall response time. A positive correlation was observed between the average time required by the ambulances to reach the hospital from the scene and the average district area covered per unit ambulance, highlighting that the districts with ambulances covering a higher catchment area expressed delay in reaching hospitals from the scene for both emergency and hospital-to-hospital transfer cases. The study also found that 27 out of 36 districts had less than one ambulance per 100,000 people. Therefore, both correlations showcase that the number of available ambulances critically impacts the quality of MEMS in terms of episode time. A similar event was observed in South Africa, where an increase in vehicles could reduce response time^5^.

Figure 7, demonstrating a high positive correlation between the number of hospitals in the district and the number of emergency and hospital-to-hospital transfer calls, indicates that health infrastructure availability in terms of the ‘number of health care services present in the district’ strongly influences the utilisation of the MEMS scheme through increasing the volume of calls.

#### Model estimation

In total, four multiple linear regression models were conducted, two for emergency calls and two for hospital-to-hospital transfer calls, to understand the determinants of travel time for MEMS ambulances (refer to Table 1). All variables with a P-value of < 0.25 on bivariate analysis were included in the multivariate regression model.

**Table 1:** Linear regression estimates of travel time from ambulance base to scene (t2) and travel time from scene to the hospital (t4) for emergency and hospital-to-hospital transfer cases.

Dependent variable	Emergency call				Hospital to hospital transfer call			
	Travel time from base to scene		Travel time from scene to hospital		Travel time from base to scene		Travel time from scene to hospital	
Independent variables	Standardized coefficient	t-stat	Standardized coefficient	t-stat	Standardized coefficient	t-stat	Standardized coefficient	t-stat
Total number of hospital in the district	− 0.290	− 1.757*			− 0.465	− 2.687**		
Population density (district level)	− 0.546	− 3.196***	− 0.402	− 2.441**	− 0.273	− 1.528	− 0.413	− 2.617**
Road length per 100 km^2^	− 0.259	− 1.789*	− 0.293	− 1.825*	− 0.112	− 0.736	− 0.276	− 1.795*
Area (in km^2^) covered per unit ambulance			0.395	2.520**			0.427	2.839***
If 1 ambulance serving more than 100,000 population	0.233	1.671*			0.394	2.700**		
Goodness of fit								
Sample size	35		35		35		35	
Adjusted R square	0.406		0.433		0.347		0.479	

*Significant at 90% CI, ** Significant at 95% CI and *** Significant at 99% CI.; Mumbai and Mumbai suburban has been considered together.

Five infrastructural characteristics were found to be significantly associated with the travel time of ambulances. For both emergency calls and hospital-to-hospital transfer calls, the factors were found to be population density, road density, the total number of hospitals, the area covered per unit ambulance, and whether one ambulance was serving more than 100,000 people.

The population density of a district showed a negative relationship with the ambulance travel time from base to scene and from scene to hospital, both for emergency and hospital-to-hospital transfer cases. Table 1 demonstrates that, for each unit decrease in population density (i.e., total number of populations residing per sq. km of district area), the ambulance travel time (in minutes) from base to scene and from scene to hospital is expected to increase by 0.546 and 0.402 units, respectively, for emergency calls and by 0.273 and 0.413 units, respectively, for hospital-to-hospital transfer calls, holding all other variables constant. This finding indicates that the MEMS ambulance would require more time to reach the scene from the base in sparsely populated districts than densely populated districts.

A negative association was observed between the road density of a district and ambulance travel time from base to scene and from scene to hospital for both emergency and hospital-to-hospital cases. For each unit increase in the road length per 100 km^2^ (i.e., total length of roads in km per sq. km of district area), the ambulance travel time (in minutes) from base to scene and from scene to hospital is expected to decrease by 0.259 and 0.293 units, respectively, for emergency calls, holding all other variables constant. This indicates that districts with higher road networks involve less travel time. Improvements in road infrastructure and an increase in road connectivity help the mobile healthcare delivery system reach the scene on time.

The current models also indicate a negative and statistically significant relationship between the total number of hospitals in the district and ambulance travel time from base to the scene for emergency and hospital-hospital transfer cases. It was found that if the number of hospitals increases by one unit, the ambulance travel time (in minutes) from the base to the scene is expected to decrease by 0.290 for emergency calls and by 0.465 for hospital-to-hospital transfer calls, while holding all other variables constant. Owing to the primary location of ambulance base within different hospital campuses, the districts having a higher number of hospitals spread across different locations experienced lesser ambulance travel time from base to scene. This indicates that increased healthcare infrastructure would aid in reducing the average response time (t1 + t2) of the MEMS ambulance service.

The models further estimated a positive and statistically significant association between the area covered per unit ambulance and ambulance travel time from scene to hospital for both cases. The coefficients of 0.395 and 0.427 represents the rate of change in travel time (in minutes) for emergency and hospital-to-hospital transfer calls concerning the area covered by each ambulance (i.e., sq.km of district area covered by one ambulance). This indicates that in the districts where few ambulances serve a particular area, the ambulance requires more time to travel from scene to hospital, thereby increasing the EMS challenges. Therefore, increasing the number of ambulances for lesser service areas would decrease the average travel time.

Lastly, a positive and statistically significant relationship was found between the population served by one ambulance and ambulance travel time from base to scene for both cases. This study has identified that only for nine out of 36 districts in Maharashtra, one ambulance is serving less than 100,000 population. This phenomenon, indicating a lack of ambulances per 100,000 population, has increased the ambulance travel time from base to scene, delaying the overall episode time and reducing timeliness.

### Determinants of ambulance response time in transportation from qualitative finding

Ten interviews were conducted with medical officers at MEMS ERC and officials from the Dept. of Health, Govt. of Maharashtra, to deeply understand the determinants of ambulance response time, particularly the patient assessment time at the scene and patient handover time at hospitals.

The results of the qualitative study showed different factors that influence ambulance response time. In general, the qualitative study identified the following factors as timeliness determinants: socio-cultural reasons affecting health-seeking behaviour, early closing of healthcare centres, undercapacity and resource-deficit healthcare centres, and overloading of specialised tertiary hospitals.

“Pregnancy is the leading health issue for emergency calls, as pregnant women, even with extreme labour pain (at the final stage of delivery), often tend to wait for their husbands to return from work and then contact MEMS, thereby aggravating the emergency situation. This situation leads to two cases: (i) the medical team with an ambulance reaches the scene and conducts delivery at home as prehospital treatment, and (ii) the medical team conducts the delivery at the ambulance, depending on the emergency level.” Medical Officer, MEMS ERC. District-wise and time-wise details of pregnancy call distribution are presented in Appendix IV.

In addition to this “nearest primary healthcare centres often close down at 4 p.m., compelling the ambulances to travel a longer distance to reach tertiary hospitals with 24-h facilities, which might be distant”. Medical Officer, MEMS ERC.

“The specialised hospitals are generally overloaded and overstretched. Hence, the patient handover time increases due to the unavailability of authorised medical personnel and other required workforce.” Medical Officer, MEMS, ERC.

## Discussion

The study looks into the service delivery of MEMS (108-Dial Ambulance Service) regarding district-level performance and transfer of patients in Maharashtra. Overall, male patients used MEMS ambulance services slightly more often than females for emergency cases. Similar observations were noted in Bangladesh, where women suffering illness report seeking care significantly less often than men^21^. The ambulance utilization level across all districts was noted as maximum for males with trauma or injury and females with gynaecologic emergencies (pregnancy or obstetric). This finding is in line with studies conducted in other parts of India^19^ and the Tigray and Addis Ababa regions of Ethiopia^22,23^. The context-specific socio-cultural factors include the tendency of low-income females to delay seeking healthcare to avoid wage loss, dependence on male family members for accompanying healthcare visits^24^, prevalent altruistic behaviour causing delays when assistance is unavailable, the desire to reduce out-of-pocket expenses, and countering a lack of insurance, ultimately reducing disability-adjusted life years (DALY). Sindhudurg district recorded high emergency calls from males with high head injury cases as a prominent disease among all districts since the Konkan belt is prone to accidents^25^.

The study found that out of 36 districts in Maharashtra, Chandrapur and Sindhudurg have recorded the highest emergency and hospital-to-hospital transfer calls per 100,000 population. This phenomenon can be attributed to the following reasons: (i) lower population density of Chandrapur (193/km^2^) and Sindhudurg (163/km^2^) than the district average (940/km^2^); (ii) lower populations served per hospital in Chandrapur (153) and Sindhudurg (192) than the district average (210); (iii) higher road density in Sindhudurg (154) in comparison to the district average (104). The increased number of hospitals per population allowed the district residents to access the MEMS facility easily. In contrast, augmented road connectivity improved the MEMS performance, increasing its reliability among the population. This finding aligns with a study conducted in West Bengal, India, which identifies that the healthcare seeking episode is often influenced by the availability of physical and healthcare infrastructure, as well as personal, household, occupational characteristics, and latent perceptions of the health-seeker^26^.

Ambulance response time turns out to be most critical, as studies conducted in diverse settings in Bhutan^27^, the UK^28^, Germany^29^, and the US^30^ have established that a reduction in response time improves the odds of survival, further reducing morbidities and mortalities. Hence, understanding factors affecting ambulance travel time, a significant component of ambulance response time, becomes essential. This study found that five factors determine the travel time of MEMS ambulances in all districts of Maharashtra. These were population density, road density, the total number of hospitals, the area covered per unit ambulance, and whether one ambulance was serving more than 100,000 people.

The ambulance travel time, a critical parameter of MEMS episodes, was found to be higher in districts with a lower population density. This finding aligns with studies done in Singapore^31^, where it was observed that areas in the far outskirts (suburbs), such as Boon Lay (in the west) and Sembawang (in the north), with lower population density, fared badly in terms of ambulance response times. Response time in rural settings with lower population density was found to be longer than in urban settings in Qatar^32^. Additionally, with the increase in road density, the response time was reduced. This finding aligns with studies conducted in the USA, where researchers have specifically investigated road density as a factor contributing to the EMS response time in transportation, resulting in additional response delay^33^. Furthermore, the response time increased due to the lesser number of hospitals and ambulances. This finding is consistent with a study conducted in Jimma City, Ethiopia, where a combination of a limited number of ambulances and poor road infrastructure were found to be major challenges to prehospital care^34^.

When investigating ambulance response time in transportation, the district-wise spatial analyses highlighted that Raigad required more time at the scene to travel from scene to hospital, hand over patients at the hospital, and return to the base from the hospital, exceeding the ‘golden hour’ adopted by MEMS, i.e., a patient has to be shifted within the first hour (after receiving the call) to the nearest hospital. This phenomenon can be attributed to the determinants of travel time found in this study, i.e., a lower number of ambulances (23 < district average 27), a lower population density (368/km^2^ < district average 940/km^2^), and lastly, a lesser area covered per ambulance (311 km^2^/unit ambulance < district average 400 km^2^/unit ambulance). Sparse location, remote road connectivity, and a lack of adequate health infrastructure have increased the Raigad district’s average response time. Literature has identified that the Gadchiroli district suffers from increased cases of time-sensitive emergencies such as stroke and rheumatic diseases^35–38^. This phenomenon might have led to delays in time required at the scene (t3), catering to prehospital care at the patient location for emergency calls in this district.

The findings of the qualitative study show distinct socio-cultural reasons affecting health-seeking behaviour, early closure of healthcare centres, resource-deficit and undercapacity healthcare centres, and overloaded specialised hospitals as significant determinants of delay in ambulance response time, particularly t3, t4, and t5. The observation of early closure of nearby healthcare centres and the unavailability of nearby centres with required facilities leading to an increase in travel time to reach an available hospital validates the Raigad district's taking more than 100 min to reach the nearest hospital from the scene. The finding of increased ambulance response time, particularly patient handover time, in Raigad owing to overcrowding of emergency departments at tertiary hospitals is in line with studies conducted in the diverse settings of developed and developing nations, where overcrowding of emergency departments at tertiary hospitals impacts patient health^39–41^.

### Limitations

However, the limitations of the current study are as follows: this study was conducted based on data available for one month, which should be extended for more extensive periods to analyse the impacts of a) seasonal variations (impact of rains/flood, landslides), b) variations in disease patterns with the change of weather, and c) increased industrial activity and pollution level. Additionally, outcome data related to feedback from the served patient needs to be further studied to understand quality and satisfaction from the user perspective.

## Conclusion

This study focuses on emergency medical services (EMS) exclusively for the state of Maharashtra in India. The researchers sequentially employed quantitative and qualitative methods to understand the current MEMS operational challenges and further discover the factors that influence delays in MEMS ambulance response time in transportation for different districts of Maharashtra. The study concludes that ambulance response time in Maharashtra was affected by infrastructure and planning-related factors as well as various socio-cultural and socio-economic reasons.

The overall health episode can be augmented by improvising on the supply scenario of the ambulances. In certain districts, there might be a need to augment the number of ambulances compared to the overall area of the district. Furthermore, road connectivity could be a possible consideration, especially in remote rural districts. In districts with larger areas, there is a potential for: (i) optimizing ambulance placement to minimize response time; (ii) considering expansion of the ambulance fleet to balance the larger coverage area; and (iii) considering upgradation of public health facilities such that they could be incorporated as recognized “Emergency Health Service Providers."

Nevertheless, it is crucial to bear in mind that EMS in Maharashtra has evolved rapidly since its inception, and it still needs time to adapt, mature, and strengthen over time. The subsequent action involves setting up a detailed, publicly accessible, user-friendly mobile application together with a dashboard incorporating live tracking and collecting feedback on the outcomes of the patients who availed of the service. From the supply perspective, automatized monthly report system with key performance indicators such as response time metrics, call volumes, spatial distribution of ambulances, historical trends, equipment and staff allocation and monitoring, relevant benchmarks, and audit filters should be incorporated to interpret the trend over time and make location-specific informed decisions in a subdistrict, urban, or rural area, etc.

This study, by adding to the current body of evidence on factors influencing EMS in the context of Maharashtra, holds implications for the future development of health infrastructure planning, the upgradation and augmentation of public health facilities, setting up procurement priorities based on incident-, need-, and disease-based requirements, and the formulation of emergency health service policies. The findings of this study can be used as policy determinants for improving the quality of EMS in Maharashtra in the future. While conducted specifically for the state of Maharashtra, this study could provide guidance for other states across India to explore the current scenario, regulate, and standardise the EMS in other states, while EMS in Maharashtra could aid in creating benchmarks, adjusting it as per the contextual requirement, and creating infrastructural systems where it is unavailable.

### Supplementary Information

Supplementary Information 1.

Supplementary Information 2.

Supplementary Information 3.

Supplementary Information 4.

Supplementary Information 5.

## Data Availability

The data collected and/or analyzed during the current study is available upon reasonable request to the correspondence author.

## Abbreviations

ALS: Advanced life support ambulance
AVLT: Automatic vehicle location tracker
BLS: Basic life support ambulance
EMS: Emergency medical services
EMSO: Emergency medical service officers
ERC: Emergency response centre
ERS: Emergency response services
ETA: Estimated time of arrival
DALY: Disability adjusted life years
GCS: Glasgow Coma Scale
GIS: Geographic Information System
GPS: Geographic Position System
ICMR: Indian Council of Medical Research
MCS: Mobile communication system
MEMS: Maharashtra Emergency Medical Services
NHM: National Health Mission, Govt. of India
SC & ST: Scheduled Caste (Article 341 and 342 of the Constitution of India define as to who would be Scheduled Castes and Scheduled Tribes with respect to any State· or Union Territory)
WHO: World Health Organisation
