# Supplementary material for: Examining district-level disparity and determinants of timeliness of emergency medical services in Maharashtra, India
Source: Sci Rep. 2023 Dec 1;13:21239. doi: 10.1038/s41598-023-48713-1 (PMC10692338; doi:10.1038/s41598-023-48713-1)
Supplement: Supplementary file 1 — Supplementary Information 1. [file 41598_2023_48713_MOESM1_ESM.docx]

**Appendix I**

**District-wise demographic details of Maharashtra**

| Appendix I: Table 1: District of Maharashtra, Population and Area | | | | |
| --- | --- | --- | --- | --- |
| District Name | **Population** | | | **Area  (In sq. km)** |
|  | **Males** | **Females** | **Total** |  |
| Ahmadnagar | 23,42,825 | 22,00,334 | 45,43,159 | 17,048 |
| Akola | 9,32,334 | 8,81,572 | 18,13,906 | 5,673 |
| Amravati | 14,80,768 | 14,07,677 | 28,88,445 | 12,210 |
| Aurangabad ### | 19,24,469 | 17,76,813 | 37,01,282 | 10,131 |
| Bhandara | 6,05,520 | 5,94,814 | 12,00,334 | 4,087 |
| Bid | 13,49,106 | 12,35,943 | 25,85,049 | 10,693 |
| Buldana | 13,37,560 | 12,48,698 | 25,86,258 | 9,661 |
| Chandrapur | 11,23,834 | 10,80,473 | 22,04,307 | 11,443 |
| Dhule | 10,54,031 | 9,96,831 | 20,50,862 | 7,195 |
| Gadchiroli | 5,41,328 | 5,31,614 | 10,72,942 | 14,412 |
| Gondiya | 6,61,554 | 6,60,953 | 13,22,507 | 5,234 |
| Hingoli | 6,06,294 | 5,71,051 | 11,77,345 | 4,827 |
| Jalgaon | 21,97,365 | 20,32,552 | 42,29,917 | 11,765 |
| Jalna | 10,11,473 | 9,47,573 | 19,59,046 | 7,694 |
| Kolhapur | 19,80,658 | 18,95,343 | 38,76,001 | 7,685 |
| Latur | 12,73,140 | 11,81,056 | 24,54,196 | 7,157 |
| Mumbai | 16,84,608 | 14,00,803 | 30,85,411 | 157 |
| Mumbai Suburban | 50,31,323 | 43,25,639 | 93,56,962 | 446 |
| Nagpur | 23,84,975 | 22,68,595 | 46,53,570 | 9,892 |
| Nanded | 17,30,075 | 16,31,217 | 33,61,292 | 10,528 |
| Nandurbar | 8,33,170 | 8,15,125 | 16,48,295 | 5,955 |
| Nashik | 31,57,186 | 29,50,001 | 61,07,187 | 15,530 |
| Osmanabad | 8,61,535 | 7,96,041 | 16,57,576 | 7,569 |
| Parbhani | 9,42,870 | 8,93,216 | 18,36,086 | 6,214 |
| Pune | 49,24,105 | 45,05,303 | 94,29,408 | 15,643 |
| Raigarh | 13,44,345 | 12,89,855 | 26,34,200 | 7,152 |
| Ratnagiri | 7,61,121 | 8,53,948 | 16,15,069 | 8,208 |
| Sangli | 14,35,728 | 13,86,415 | 28,22,143 | 8,572 |
| Satara | 15,10,842 | 14,92,899 | 30,03,741 | 10,480 |
| Sindhudurg | 4,17,332 | 4,32,319 | 8,49,651 | 5,207 |
| Solapur | 22,27,852 | 20,89,904 | 43,17,756 | 14,895 |
| Thane | 58,65,078 | 51,95,070 | 1,10,60,148 | 4,862 |
| Wardha | 6,68,385 | 6,32,389 | 13,00,774 | 6,309 |
| Washim | 6,20,302 | 5,76,858 | 11,97,160 | 4,901 |
| Yavatmal | 14,19,965 | 13,52,383 | 27,72,348 | 13,582 |
| Palghar | 15,45,779 | 14,44,337 | 29,90,116 | 4,696 |

Source: Census of India 2011 and District Handbook of Maharashtra


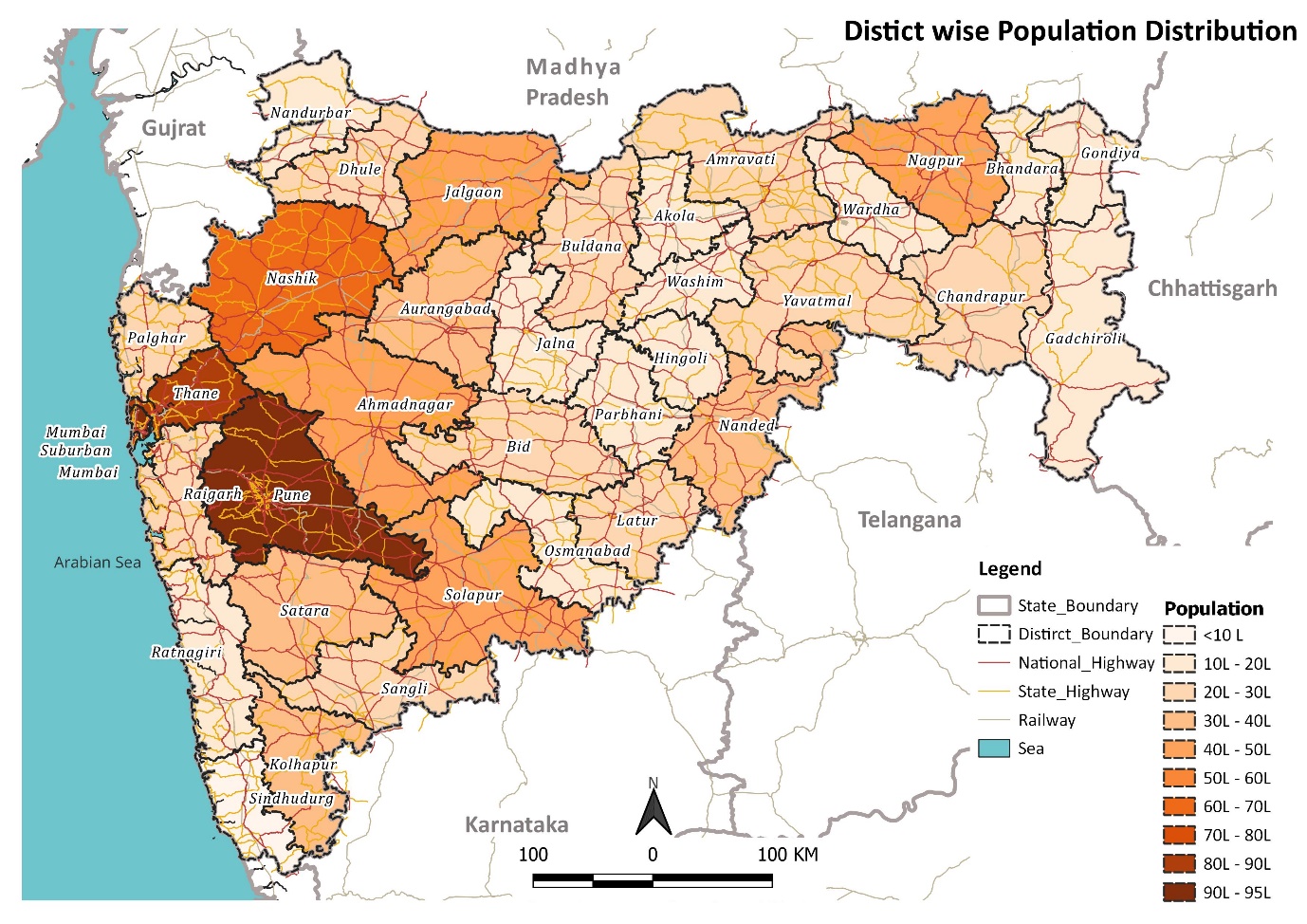


Appendix I: Figure 1: District-wise population distribution in Maharashtra. QGIS Geographic Information System v3.28.3-Firenze. QGIS.org, 2023. QGIS Association. http://www.qgis.org.


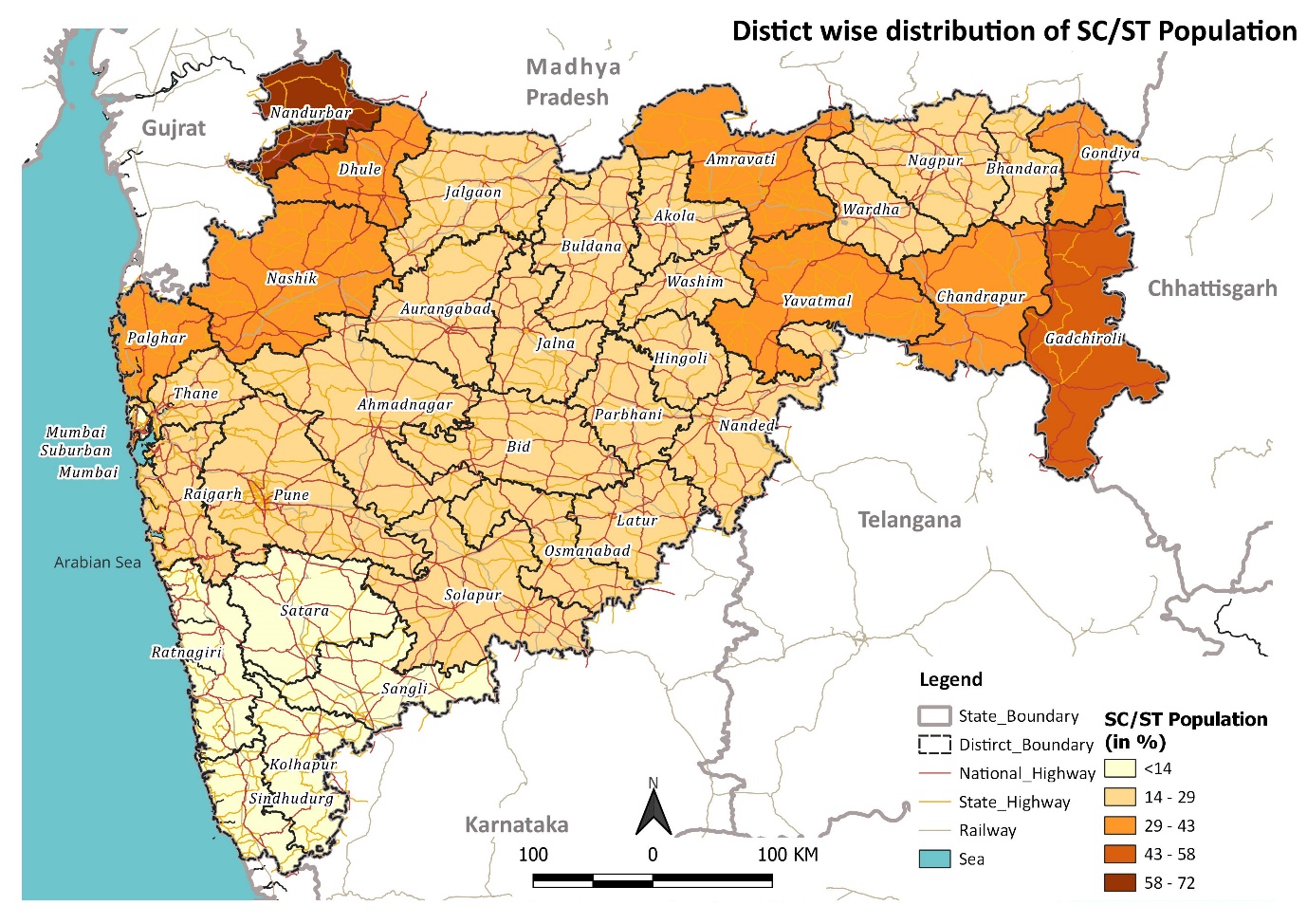


Appendix I: Figure 2: District-wise population distribution of SC/ST population in Maharashtra. QGIS Geographic Information System v3.28.3-Firenze. QGIS.org, 2023. QGIS Association. http://www.qgis.org.

**District wise details of the components of the total episode.**

| Appendix I: Table 2: Emergency Calls | | | | | | | |
| --- | --- | --- | --- | --- | --- | --- | --- |
| District Name | Mean ± SD (t1) | Mean ± SD (t2) | Mean ± SD (t3) | Mean ± SD (t4) | Mean ± SD (t5) | Mean ± SD (t6) | Mean ± SD (T) |
| Ahmednagar | 4.11 ± 5.02 | 22.86 ± 18.85 | 13.1 ± 11.77 | 65.27 ± 45.97 | 11.93 ± 9.97 | 75.5 ± 67.16 | 196.85 ± 113.69 |
| Akola | 3.55 ± 3.55 | 22.38 ± 23.83 | 13.37 ± 11.61 | 65.76 ± 39.24 | 10.51 ± 5.67 | 77.99 ± 66.68 | 197.41 ± 103.72 |
| Amravati | 4.58 ± 5.91 | 27.62 ± 22.6 | 12.89 ± 13.41 | 67.52 ± 52.93 | 10.07 ± 10.29 | 73.07 ± 75.9 | 199.16 ± 128.02 |
| Aurangabad | 4.4 ± 6.79 | 21.7 ± 18.16 | 12.83 ± 12.01 | 54.64 ± 40.69 | 11.3 ± 8.85 | 61.96 ± 58.42 | 171.32 ± 99.81 |
| Beed | 3.22 ± 4.15 | 20.82 ± 17.67 | 10.51 ± 9.88 | 46.31 ± 32.16 | 8.66 ± 7.86 | 53.39 ± 55.6 | 146.33 ± 84.28 |
| Bhandara | 5.27 ± 7.53 | 22.55 ± 18.35 | 15.79 ± 15.39 | 55.86 ± 36.58 | 14.14 ± 13.09 | 62.19 ± 60.51 | 179.78 ± 106.94 |
| Buldhana | 3.55 ± 4.43 | 19.31 ± 19.07 | 13.8 ± 12.49 | 59.19 ± 36.37 | 11.46 ± 9.14 | 74.04 ± 58.12 | 185.31 ± 95.53 |
| Chandrapur | 1.97 ± 3.05 | 19.63 ± 18.36 | 11.2 ± 7.13 | 64.78 ± 50.97 | 11.7 ± 7.85 | 65.99 ± 61.12 | 179.25 ± 114.26 |
| Dhule | 3.13 ± 2.68 | 21.34 ± 16.52 | 9.99 ± 9.82 | 43.83 ± 29.16 | 8.28 ± 4.63 | 51.6 ± 48.93 | 142.18 ± 78.57 |
| Gadchiroli | 2.33 ± 3.37 | 19.57 ± 21.02 | 16.64 ± 9.59 | 93.81 ± 73.3 | 17.06 ± 11.6 | 92.76 ± 89.31 | 246.25 ± 162.42 |
| Gondia | 2.8 ± 2.67 | 22.12 ± 19.03 | 10.2 ± 9.5 | 46.36 ± 38.24 | 8.05 ± 6.32 | 47.06 ± 54.3 | 140.38 ± 92.35 |
| Hingoli | 5.31 ± 4.08 | 19.21 ± 15.83 | 10.39 ± 8.43 | 54.16 ± 44.09 | 9.27 ± 5.31 | 58.45 ± 52.8 | 160.71 ± 96.44 |
| Jalgaon | 3.64 ± 5.39 | 18.17 ± 15.78 | 11.74 ± 10.08 | 50.82 ± 32.25 | 10.53 ± 9.93 | 58.16 ± 49.56 | 156.77 ± 81.59 |
| Jalna | 4.46 ± 7.98 | 24.63 ± 22.46 | 14.38 ± 13.75 | 64.51 ± 38.87 | 11.87 ± 10.59 | 78.41 ± 59.93 | 202.62 ± 102.22 |
| Kolhapur | 2.97 ± 3.54 | 17.71 ± 14 | 12.02 ± 10.07 | 41.72 ± 28.13 | 11.49 ± 10.12 | 57.48 ± 46.45 | 147.07 ± 74.72 |
| Latur | 4.74 ± 7.1 | 20.24 ± 19.72 | 12.59 ± 13.71 | 49.3 ± 37.64 | 9.57 ± 9.36 | 54.17 ± 55.21 | 154.35 ± 94.57 |
| Mumbai | 3.59 ± 3.27 | 10.49 ± 7.62 | 15.21 ± 12.46 | 25.47 ± 18.52 | 17.31 ± 19.08 | 36.53 ± 27.88 | 112.69 ± 54.22 |
| Nagpur | 2.69 ± 3.07 | 23.94 ± 20.89 | 13.66 ± 11.76 | 53.7 ± 36.01 | 12.5 ± 11.03 | 61.36 ± 53.91 | 172.23 ± 96.53 |
| Nanded | 4.29 ± 6.59 | 16.9 ± 17.51 | 13.16 ± 13.07 | 66.81 ± 47.61 | 11.24 ± 9.5 | 85.56 ± 74.02 | 201.55 ± 123.25 |
| Nandurbar | 3.99 ± 7.93 | 24.58 ± 23.26 | 12.35 ± 12.98 | 63.13 ± 52.26 | 9.8 ± 8.22 | 62.82 ± 65.67 | 180.54 ± 117.15 |
| Nashik | 3.93 ± 6.19 | 21.01 ± 18.74 | 12.8 ± 11.48 | 44.47 ± 31.6 | 11.51 ± 12.99 | 49.25 ± 51.41 | 146.93 ± 84.83 |
| Osmanabad | 4.82 ± 7.47 | 16.28 ± 19.62 | 10.1 ± 10.56 | 47.32 ± 34.09 | 8.95 ± 9.95 | 58.38 ± 55.53 | 149.71 ± 88.22 |
| Palghar | 2.95 ± 2.97 | 22.44 ± 19.41 | 13.5 ± 12.3 | 60.76 ± 53.43 | 15.23 ± 15.41 | 67.34 ± 64.64 | 186.45 ± 123.66 |
| Parbhani | 3.94 ± 4.57 | 18.12 ± 19.63 | 12.22 ± 11.54 | 56.13 ± 38.24 | 11.57 ± 11.89 | 84.04 ± 70.88 | 190.23 ± 107.74 |
| Pune | 3.24 ± 4.34 | 17.85 ± 16.96 | 15.22 ± 14.16 | 47.07 ± 42.31 | 14.41 ± 12.79 | 55.59 ± 54.65 | 157.96 ± 102.98 |
| Raigad | 3.54 ± 8.39 | 24.62 ± 26.31 | 20.9 ± 22.09 | 90.01 ± 62.94 | 29.17 ± 39.95 | 104.78 ± 79.22 | 278.03 ± 163.64 |
| Ratnagiri | 2.5 ± 3.16 | 23.05 ± 23.16 | 16.69 ± 14.78 | 76.18 ± 57.51 | 9.94 ± 8.07 | 91.12 ± 73.83 | 223.31 ± 133.83 |
| Sangli | 3.64 ± 5.03 | 19.57 ± 16.19 | 12.8 ± 11.49 | 51.44 ± 35.48 | 12.24 ± 11.27 | 66.12 ± 53.66 | 169.4 ± 92.27 |
| Satara | 3.42 ± 4.09 | 19.06 ± 16.93 | 12.39 ± 11.77 | 47.38 ± 34.48 | 10.49 ± 7.98 | 54.37 ± 51.15 | 150.75 ± 84.6 |
| Sindhudurg | 1.82 ± 3.19 | 18.55 ± 19.76 | 13.9 ± 13.64 | 51.62 ± 33.38 | 9.95 ± 9.13 | 65.33 ± 57.47 | 164.98 ± 94.5 |
| Solapur | 2.74 ± 4.15 | 23.59 ± 19.98 | 11.34 ± 10.76 | 50.48 ± 34.08 | 10.5 ± 9.16 | 58.42 ± 51.23 | 160.72 ± 87.39 |
| Thane | 3.21 ± 5.08 | 20.01 ± 18.14 | 17.66 ± 14.76 | 55.56 ± 33.19 | 17.46 ± 16.44 | 67.08 ± 48.47 | 185.86 ± 91.75 |
| Wardha | 3.36 ± 3.58 | 21.29 ± 20.35 | 11.38 ± 9.14 | 54.69 ± 32.71 | 9.59 ± 6.83 | 65.86 ± 58.22 | 170.18 ± 90.12 |
| Washim | 3.22 ± 2.5 | 26.54 ± 21.58 | 13.39 ± 11.21 | 73.04 ± 42.8 | 11.01 ± 5.78 | 84.31 ± 61.61 | 215.69 ± 104.75 |
| Yavatmal | 3.68 ± 3.37 | 20.26 ± 21.15 | 12.25 ± 10.48 | 65.61 ± 44.13 | 9.84 ± 6.47 | 86.48 ± 72.66 | 201.97 ± 113.98 |
| Note: Mumbai and Mumbai Suburban has been considered together | | | | | | | |

| Appendix I: Table 3: Hospital-to-hospital transfer calls | | | | | | | |
| --- | --- | --- | --- | --- | --- | --- | --- |
| District Name | **Mean ± SD (t1)** | **Mean ± SD (t2)** | **Mean ± SD (t3)** | **Mean ± SD (t4)** | **Mean ± SD (t5)** | **Mean ± SD (t6)** | **Mean SD ± (T)** |
| Ahmednagar | 5.26 ± 8.94 | 20.26 ± 19.64 | 14.64 ± 14.83 | 87.35 ± 44.54 | 13.18 ± 9.83 | 112.47 ± 63.25 | 258.08 ± 107.95 |
| Akola | 5.1±9.01 | 18.4±25.83 | 11.5±9.42 | 76.6±41.07 | 13.11±12.4 | 99.47±69.57 | 228.12±110.38 |
| Amravati | 5.04±6.43 | 18.53±23.21 | 15.52±14.56 | 98.23±63.35 | 13.12±11.67 | 126.6±87.38 | 280.89±151.21 |
| Aurangabad | 4.95±9 | 16.29±20.48 | 14.7±13.51 | 74.18±46.74 | 12.32±9.17 | 93.27±62.9 | 220.44±107.21 |
| Beed | 3.58±7.79 | 11.14±14.64 | 10.84±11.68 | 67.27±42.32 | 8.48±6.57 | 87.75±59.77 | 192.43±103.11 |
| Bhandara | 4.41±3.93 | 22.65±23.22 | 17.91±14.36 | 76.79±39.27 | 17.8±14.86 | 104.37±59.81 | 248.39±107.07 |
| Buldhana | 3.76±5.23 | 19.89±22.05 | 14.88±12.2 | 75.29±37.1 | 11.95±7.6 | 100.28±56.86 | 230.32±90.73 |
| Chandrapur | 1.8±1.93 | 14.29±19.22 | 11.38±7.04 | 90.08±49.16 | 12.65±7.36 | 97.36±58.69 | 231.5±108.4 |
| Dhule | 3.21±4.98 | 18.09±18.32 | 9.97±10.17 | 61.62±32.67 | 8.72±4.41 | 82.07±58.23 | 187.62±90.41 |
| Gadchiroli | 2.38±2 | 18.45±25.11 | 18.73±12.24 | 134.81±71.13 | 19.11±11.39 | 133.36±86.87 | 331.12±152.05 |
| Gondia | 3.54±4.56 | 19±22.18 | 10.47±9.5 | 73.94±38.46 | 8.67±5.24 | 86.67±62.7 | 206.27±96.41 |
| Hingoli | 5.92±6.08 | 13.72±17.18 | 10.53±9.02 | 82.73±47.06 | 11±6.03 | 104.1±64.1 | 232.1±109.43 |
| Jalgaon | 4.21±5.61 | 14.92±15.68 | 13.2±13.12 | 70.38±34.75 | 11.78±9.32 | 86.77±53.2 | 205.21±84.77 |
| Jalna | 5.71±10.52 | 21.88±26.99 | 15.82±16.06 | 86.89±36.05 | 13.12±10.59 | 117.87±54.9 | 265.98±86.35 |
| Kolhapur | 3.2±3.6 | 16.67±15.82 | 14.29±11.81 | 52.88±26.98 | 13±10.78 | 79.04±54.87 | 183.05±81.71 |
| Latur | 5.52±12.39 | 14.43±19.77 | 13.89±15.47 | 70.56±39.24 | 12±12.26 | 89.97±63.74 | 210.47±101.5 |
| Mumbai | 3.69±4.32 | 9.43±7.05 | 17.77±13.85 | 32.09±21.14 | 23.5±29.44 | 44.42±31.81 | 135.58±60.19 |
| Nagpur | 2.98±4.71 | 19.51±22.41 | 14.45±13.14 | 67.71±37.41 | 14.11±12.55 | 86.61±62.04 | 209.58±101.05 |
| Nanded | 4.38±5.14 | 15.76±20.74 | 13.56±11.51 | 97.96±46.19 | 11.98±7.99 | 129.66±69.49 | 276.94±108.96 |
| Nandurbar | 4.23±8.47 | 18.58±23.41 | 14.88±15.05 | 95.19±54.56 | 11.34±6.44 | 109.15±75.66 | 257.49±120.97 |
| Nashik | 3.74±5.6 | 16.24±19.42 | 13.81±12.24 | 60.7±31.49 | 13.76±15.59 | 77.42±56.19 | 189.93±85.03 |
| Osmanabad | 4.78±7.33 | 15.7±20.15 | 13.21±13.52 | 66.57±36.6 | 9.31±7.65 | 94.07±66.08 | 208.02±92.03 |
| Palghar | 3.14±2.78 | 21.72±23.54 | 16.36±16.2 | 94.71±57.98 | 18.63±16.46 | 106.01±66.87 | 265.6±130.17 |
| Parbhani | 3.39±4.04 | 17.41±21.73 | 12.45±10.14 | 76.19±32.38 | 14.17±11.5 | 119.14±60.31 | 247.34±91.43 |
| Pune | 3.57±5.26 | 16.19±19.36 | 16.32±14.69 | 61.3±50.67 | 15.49±12.09 | 77.2±66.69 | 194.93±121.63 |
| Raigad | 3.72±9.58 | 25.34±26.93 | 24.1±21.42 | 108.22±57.57 | 27.88±35.05 | 131.41±77.08 | 326.49±150.8 |
| Ratnagiri | 3.05±8.02 | 19.41±23.65 | 19.84±19.97 | 98.4±56.43 | 10.51±10.6 | 129.49±80.45 | 284.62±136.69 |
| Sangli | 4.1±5.57 | 15.64±16.7 | 13.53±11.54 | 69.53±37.09 | 13.01±13.54 | 101.21±69.62 | 220.86±101.29 |
| Satara | 3.36±4.22 | 14.46±16.62 | 14.12±12.87 | 62.35±32.13 | 11.33±9.75 | 83.18±55.61 | 192.67±85.76 |
| Sindhudurg | 1.7±1.8 | 17.11±19.85 | 16.75±13.37 | 68.07±37.63 | 11.21±7.85 | 85.23±53.99 | 203.88±93.54 |
| Solapur | 2.81±5.72 | 19.64±21.09 | 11.72±10.27 | 68.9±38.64 | 11.71±10.93 | 84.05±51.19 | 203.2±91.78 |
| Thane | 2.99±3.54 | 18.1±19.49 | 19.53±16.27 | 67.22±30.5 | 19.3±17.2 | 88.58±50.82 | 220.79±90.16 |
| Wardha | 3.34±2.7 | 20.58±22.55 | 11.97±10.9 | 69.92±29.94 | 10±7.53 | 88.48±52.41 | 208.47±80.51 |
| Washim | 2.95±2.33 | 23.21±24.12 | 14.83±12.96 | 101.62±41.49 | 12.64±9.27 | 126.36±63.34 | 285.9±107.25 |
| Yavatmal | 3.67±3.55 | 17.82±22.74 | 13.41±15.97 | 85.03±45.16 | 10.59±6.84 | 115.96±72.16 | 250.68±115.85 |
| Note: Mumbai and Mumbai Suburban has been considered together | | | | | | | |
