# Supplementary material for: Examining district-level disparity and determinants of timeliness of emergency medical services in Maharashtra, India
Source: Sci Rep. 2023 Dec 1;13:21239. doi: 10.1038/s41598-023-48713-1 (PMC10692338; doi:10.1038/s41598-023-48713-1)
Supplement: Supplementary file 2 — Supplementary Information 2. [file 41598_2023_48713_MOESM2_ESM.pptx]

## Slide 1
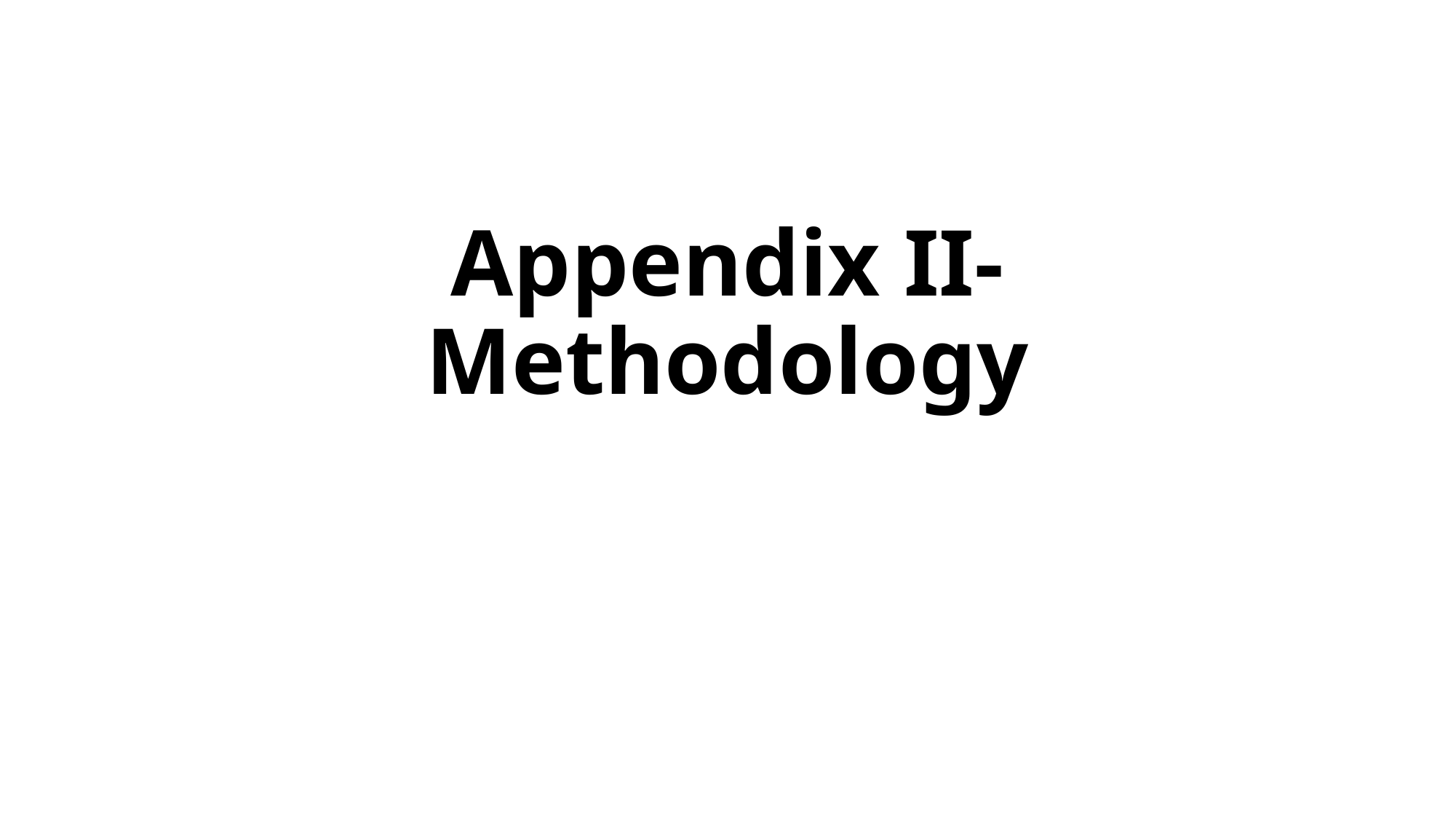

# Appendix II- Methodology

## Slide 2
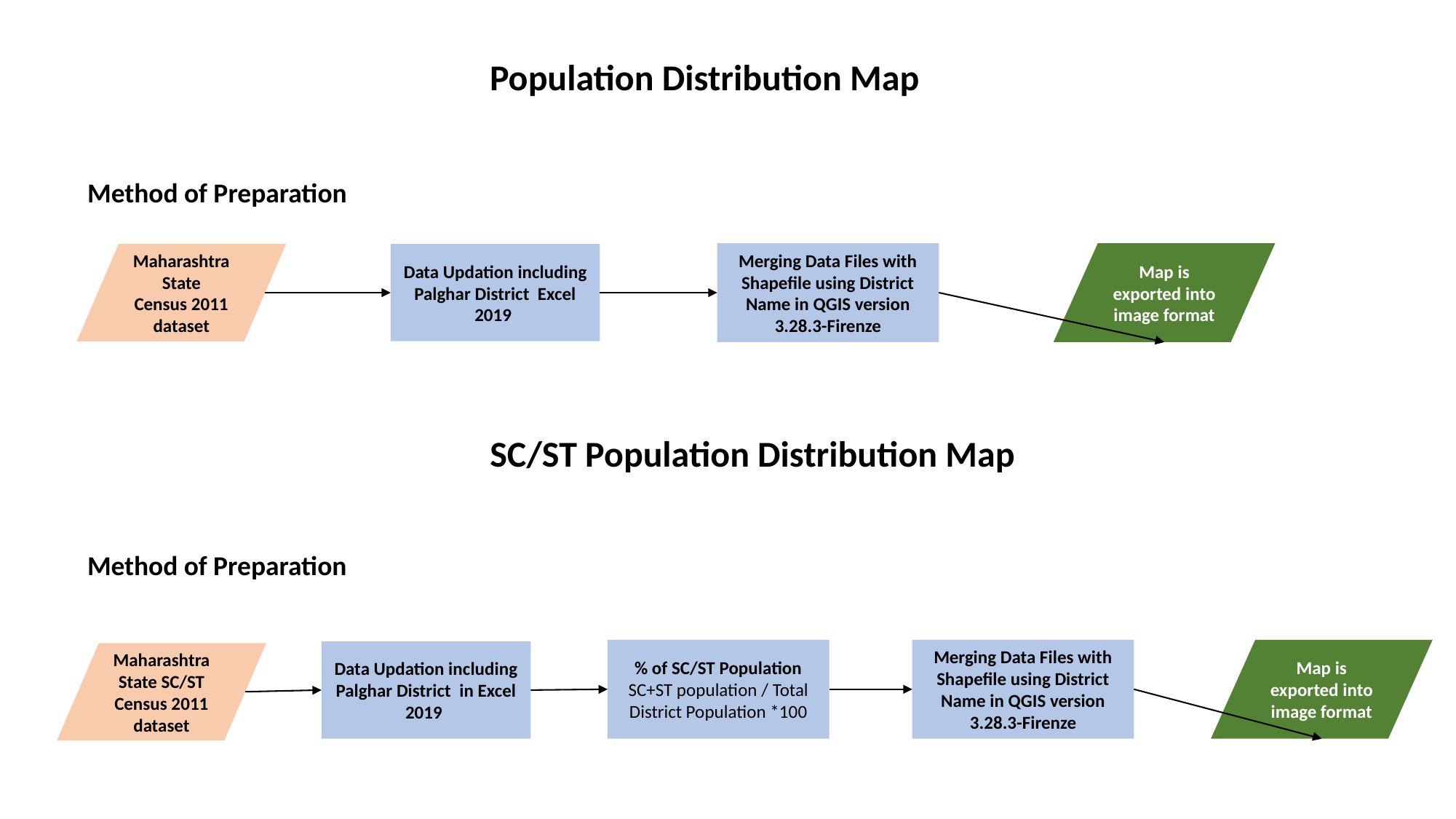

Population Distribution Map
Method of Preparation
Merging Data Files with Shapefile using District Name in QGIS version 3.28.3-Firenze
Map is exported into image format
Data Updation including Palghar District Excel 2019
Maharashtra State
Census 2011 dataset
SC/ST Population Distribution Map
Method of Preparation
% of SC/ST Population SC+ST population / Total District Population *100
Merging Data Files with Shapefile using District Name in QGIS version 3.28.3-Firenze
Map is exported into image format
Data Updation including Palghar District in Excel 2019
Maharashtra State SC/ST
Census 2011 dataset

## Slide 3
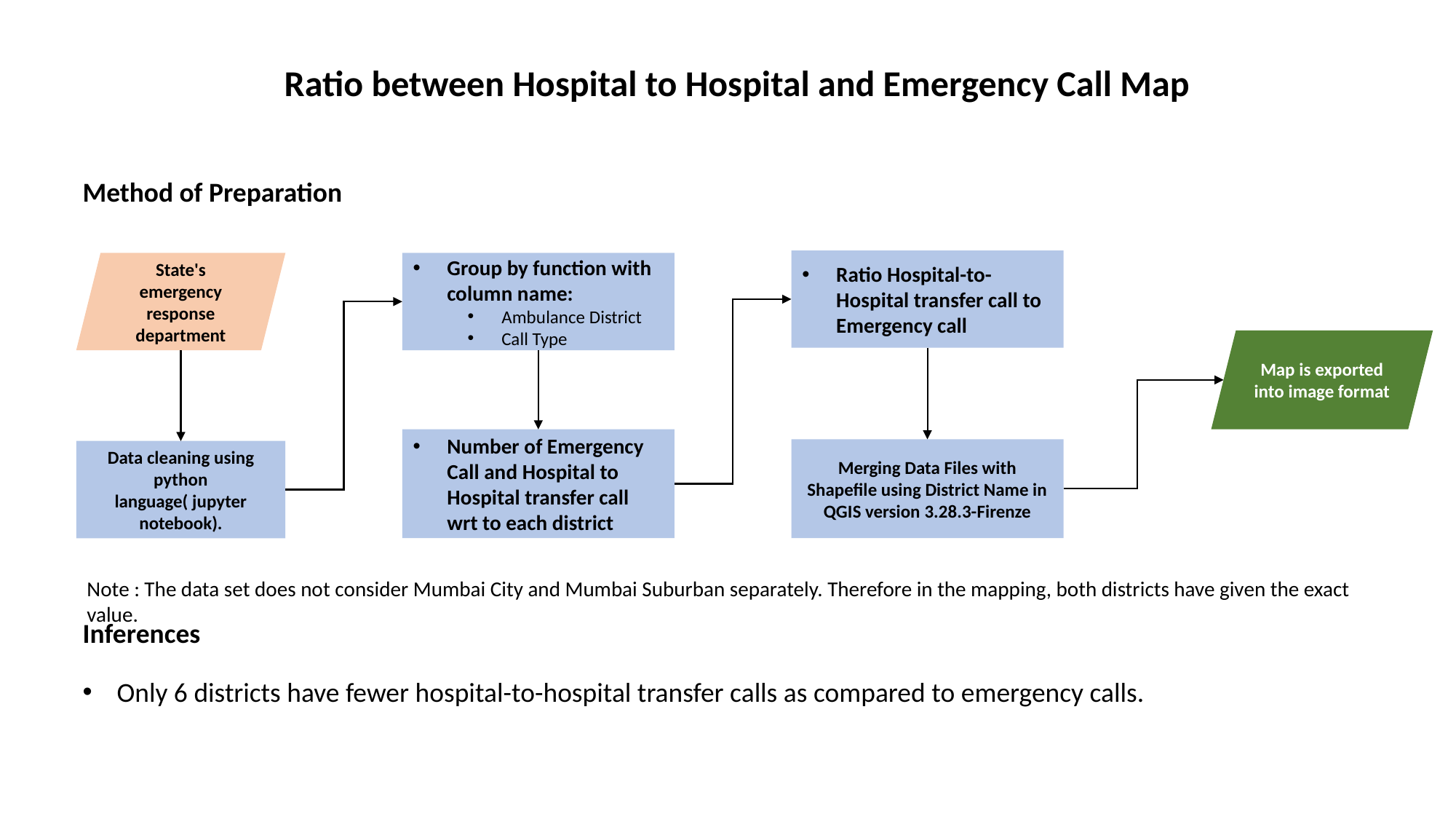

Ratio between Hospital to Hospital and Emergency Call Map
Method of Preparation
Ratio Hospital-to-Hospital transfer call to Emergency call
Group by function with column name:
Ambulance District
Call Type
State's emergency response department
Map is exported into image format
Number of Emergency Call and Hospital to Hospital transfer call wrt to each district
Merging Data Files with Shapefile using District Name in QGIS version 3.28.3-Firenze
Data cleaning using python language( jupyter notebook).
Note : The data set does not consider Mumbai City and Mumbai Suburban separately. Therefore in the mapping, both districts have given the exact value.
Inferences
Only 6 districts have fewer hospital-to-hospital transfer calls as compared to emergency calls.

## Slide 4
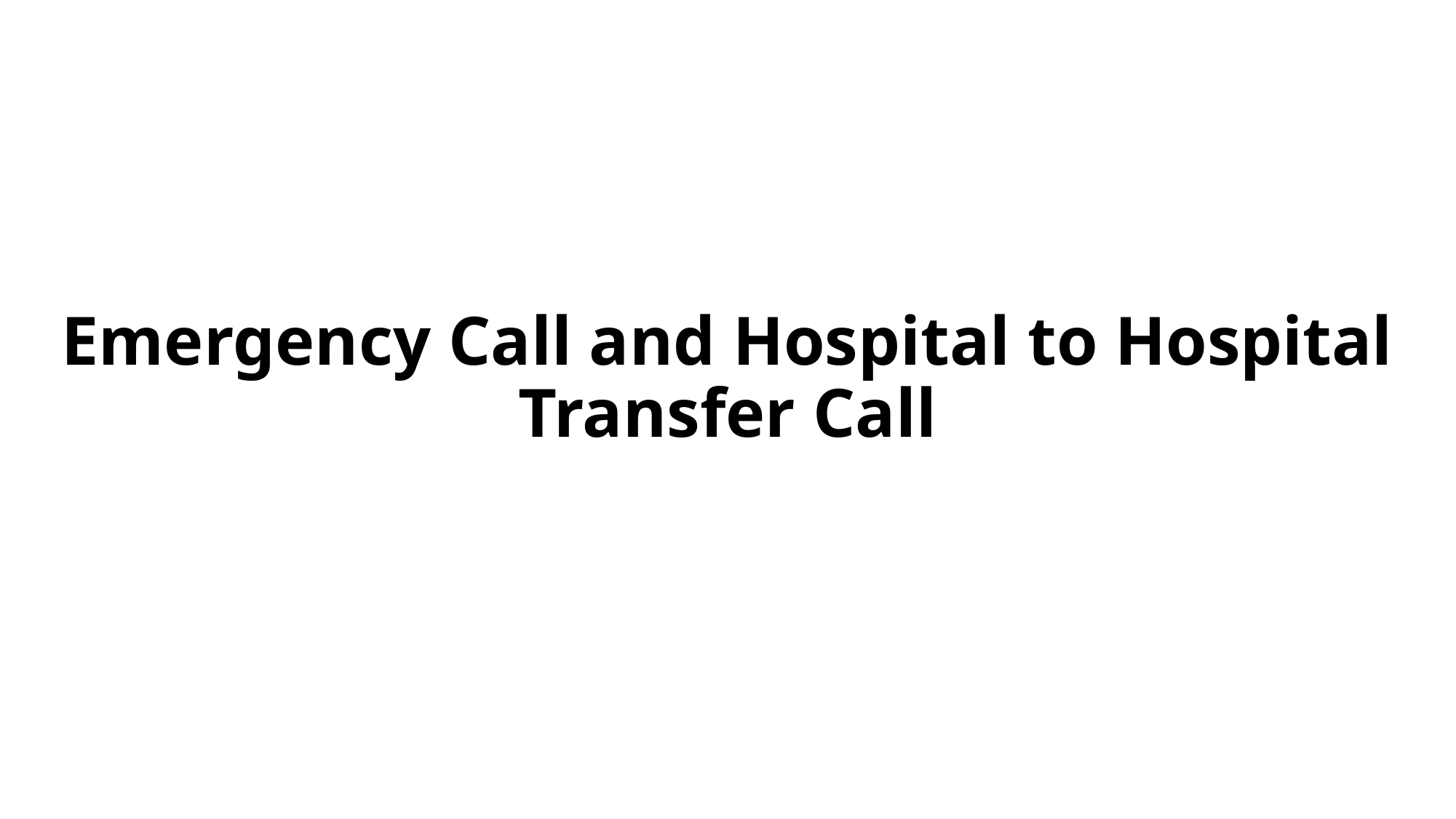

# Emergency Call and Hospital to Hospital Transfer Call

## Slide 5
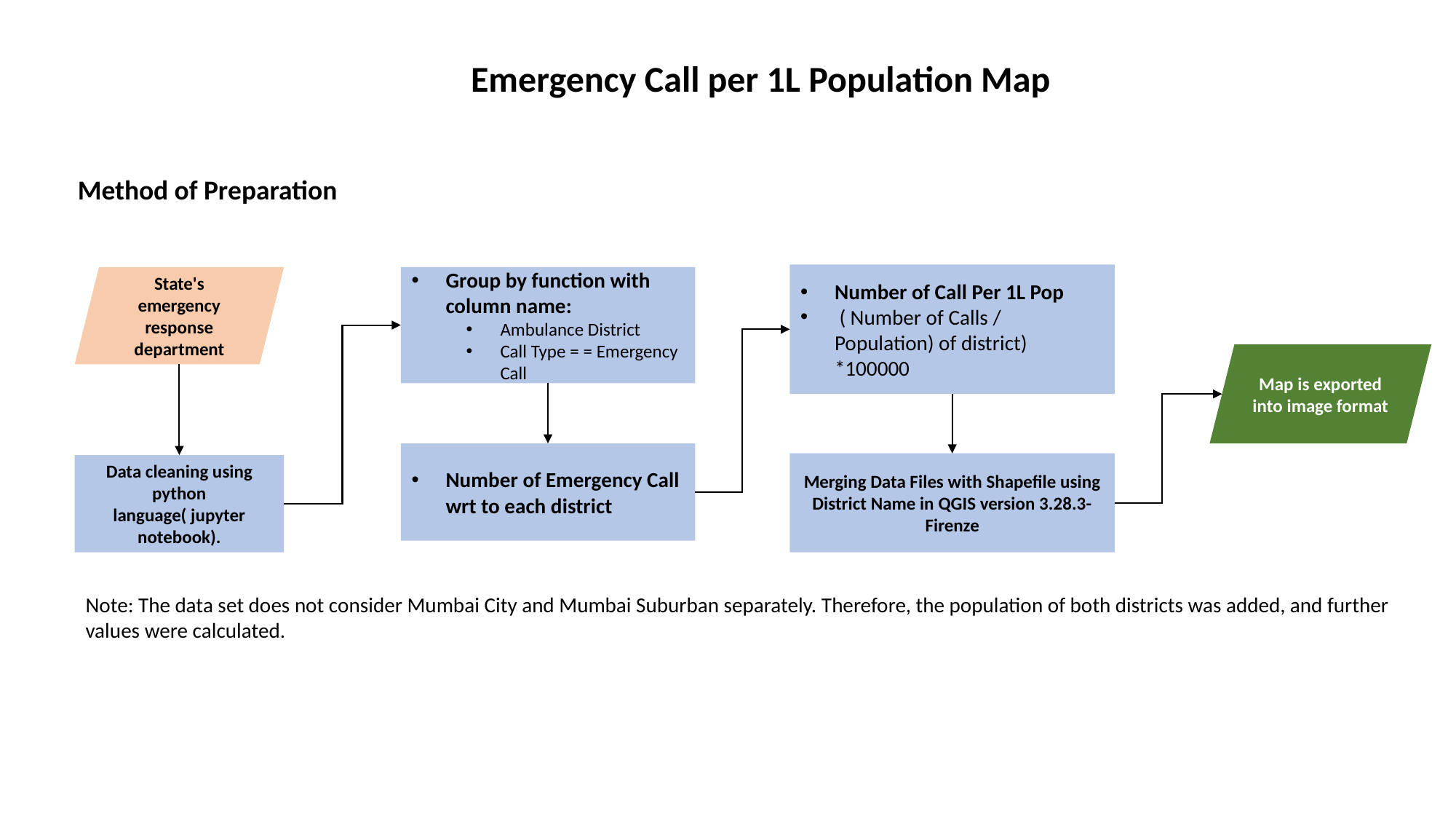

Emergency Call per 1L Population Map
Method of Preparation
Number of Call Per 1L Pop
 ( Number of Calls / Population) of district) *100000
Group by function with column name:
Ambulance District
Call Type = = Emergency Call
State's emergency response department
Map is exported into image format
Number of Emergency Call wrt to each district
Merging Data Files with Shapefile using District Name in QGIS version 3.28.3-Firenze
Data cleaning using python language( jupyter notebook).
Note: The data set does not consider Mumbai City and Mumbai Suburban separately. Therefore, the population of both districts was added, and further values were calculated.

## Slide 6
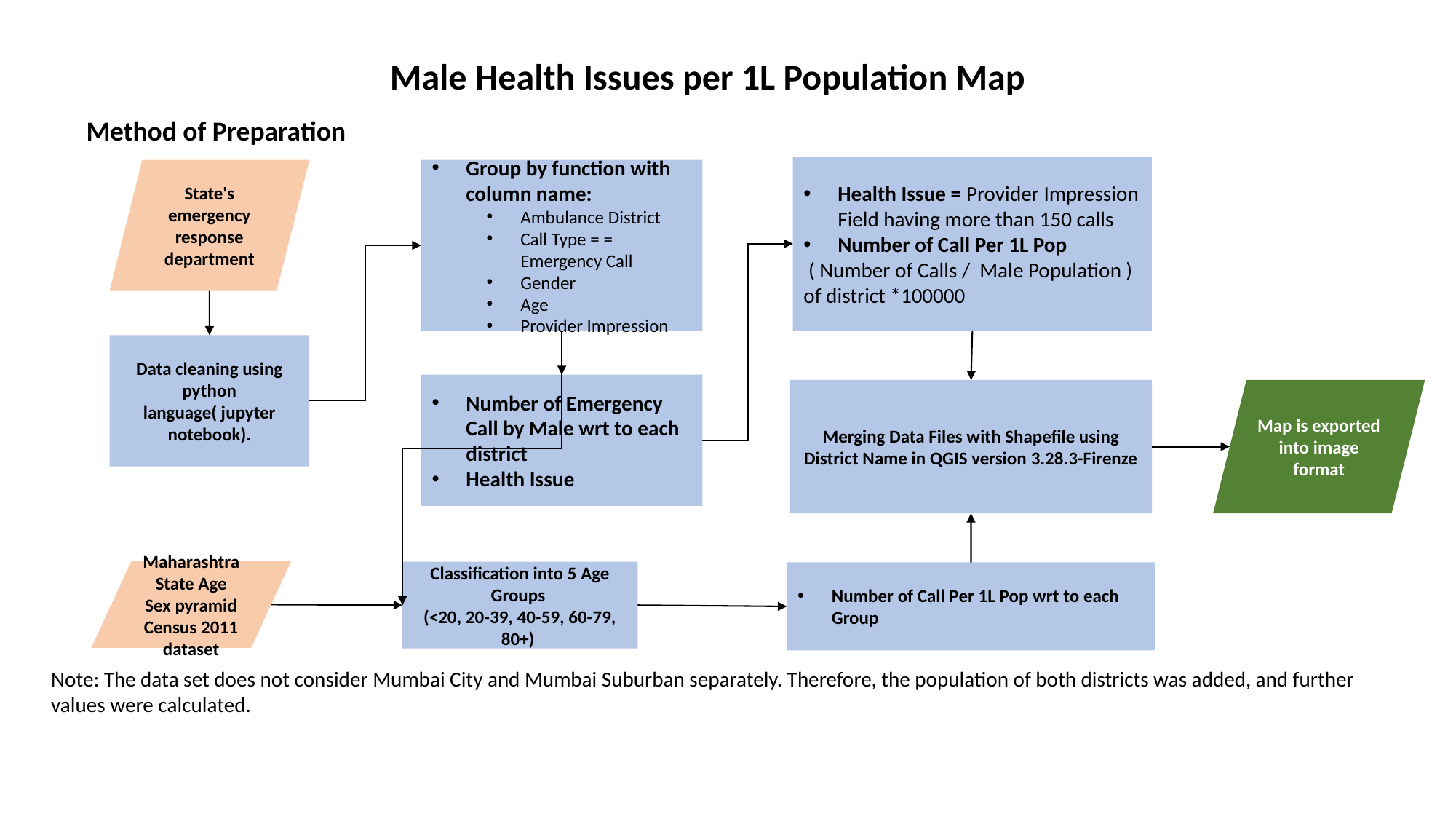

Male Health Issues per 1L Population Map
Method of Preparation
Health Issue = Provider Impression Field having more than 150 calls
Number of Call Per 1L Pop
 ( Number of Calls / Male Population ) of district *100000
Group by function with column name:
Ambulance District
Call Type = = Emergency Call
Gender
Age
Provider Impression
State's emergency response department
Data cleaning using python language( jupyter notebook).
Number of Emergency Call by Male wrt to each district
Health Issue
Map is exported into image format
Merging Data Files with Shapefile using District Name in QGIS version 3.28.3-Firenze
Maharashtra State Age Sex pyramid
Census 2011 dataset
Classification into 5 Age Groups
(<20, 20-39, 40-59, 60-79, 80+)
Number of Call Per 1L Pop wrt to each Group
Note: The data set does not consider Mumbai City and Mumbai Suburban separately. Therefore, the population of both districts was added, and further values were calculated.

## Slide 7
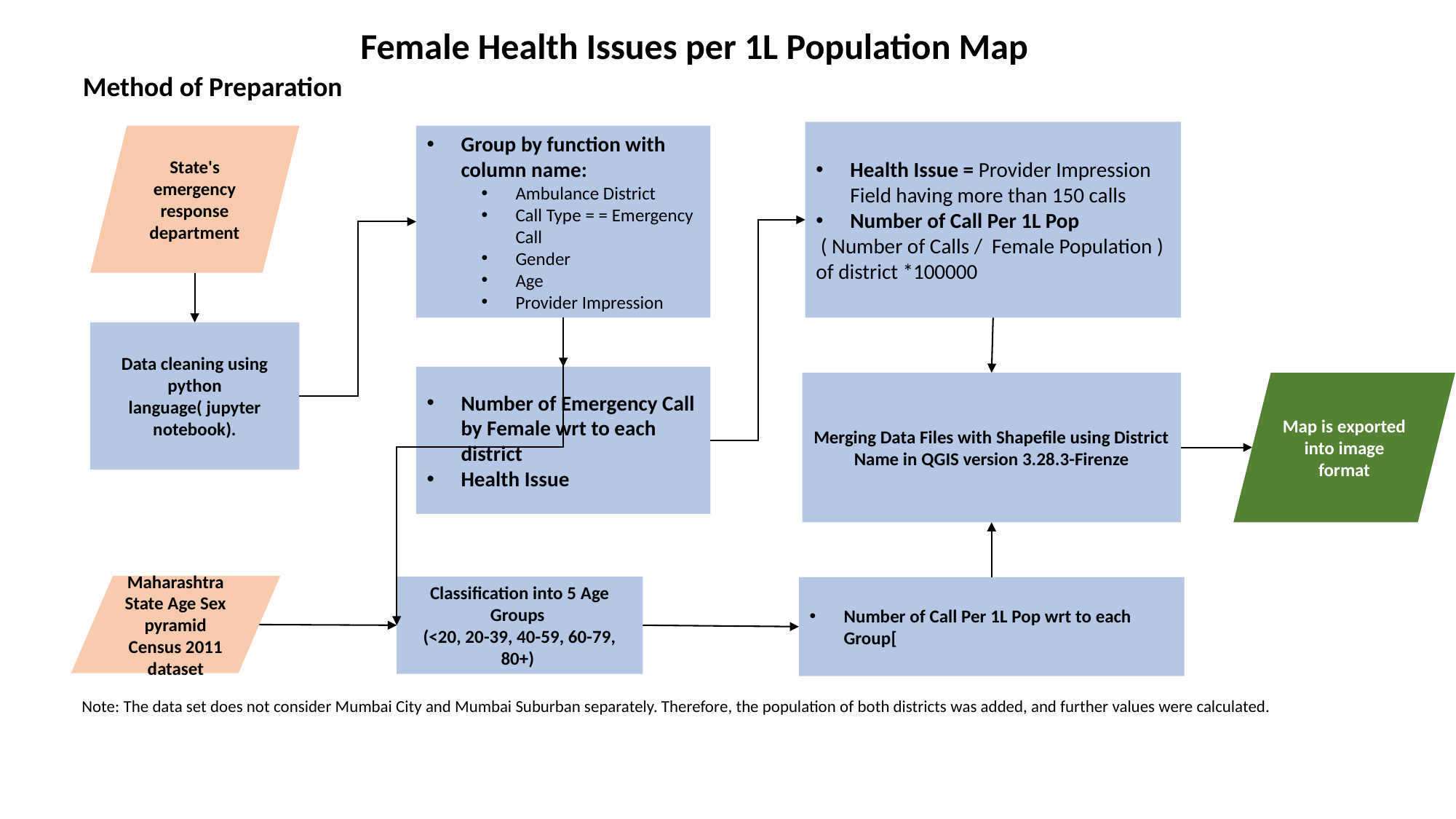

Female Health Issues per 1L Population Map
Method of Preparation
Health Issue = Provider Impression Field having more than 150 calls
Number of Call Per 1L Pop
 ( Number of Calls / Female Population ) of district *100000
Group by function with column name:
Ambulance District
Call Type = = Emergency Call
Gender
Age
Provider Impression
State's emergency response department
Data cleaning using python language( jupyter notebook).
Number of Emergency Call by Female wrt to each district
Health Issue
Map is exported into image format
Merging Data Files with Shapefile using District Name in QGIS version 3.28.3-Firenze
Maharashtra State Age Sex pyramid
Census 2011 dataset
Classification into 5 Age Groups
(<20, 20-39, 40-59, 60-79, 80+)
Number of Call Per 1L Pop wrt to each Group[
Note: The data set does not consider Mumbai City and Mumbai Suburban separately. Therefore, the population of both districts was added, and further values were calculated.

## Slide 8
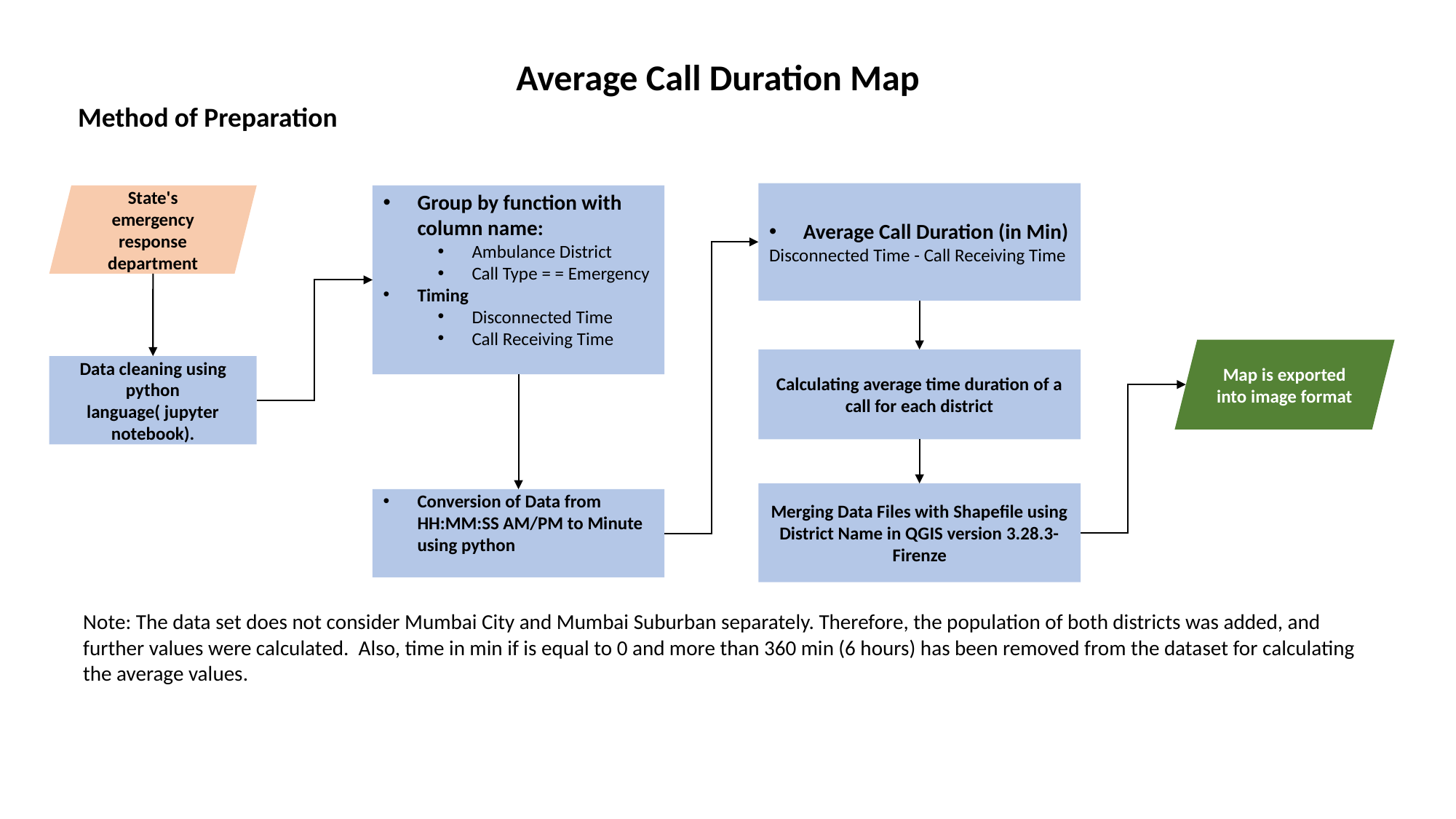

Average Call Duration Map
Method of Preparation
Average Call Duration (in Min)
Disconnected Time - Call Receiving Time
Group by function with column name:
Ambulance District
Call Type = = Emergency
Timing
Disconnected Time
Call Receiving Time
State's emergency response department
Map is exported into image format
Calculating average time duration of a call for each district
Data cleaning using python language( jupyter notebook).
Conversion of Data from HH:MM:SS AM/PM to Minute using python
Merging Data Files with Shapefile using District Name in QGIS version 3.28.3-Firenze
Note: The data set does not consider Mumbai City and Mumbai Suburban separately. Therefore, the population of both districts was added, and further values were calculated. Also, time in min if is equal to 0 and more than 360 min (6 hours) has been removed from the dataset for calculating the average values.

## Slide 9
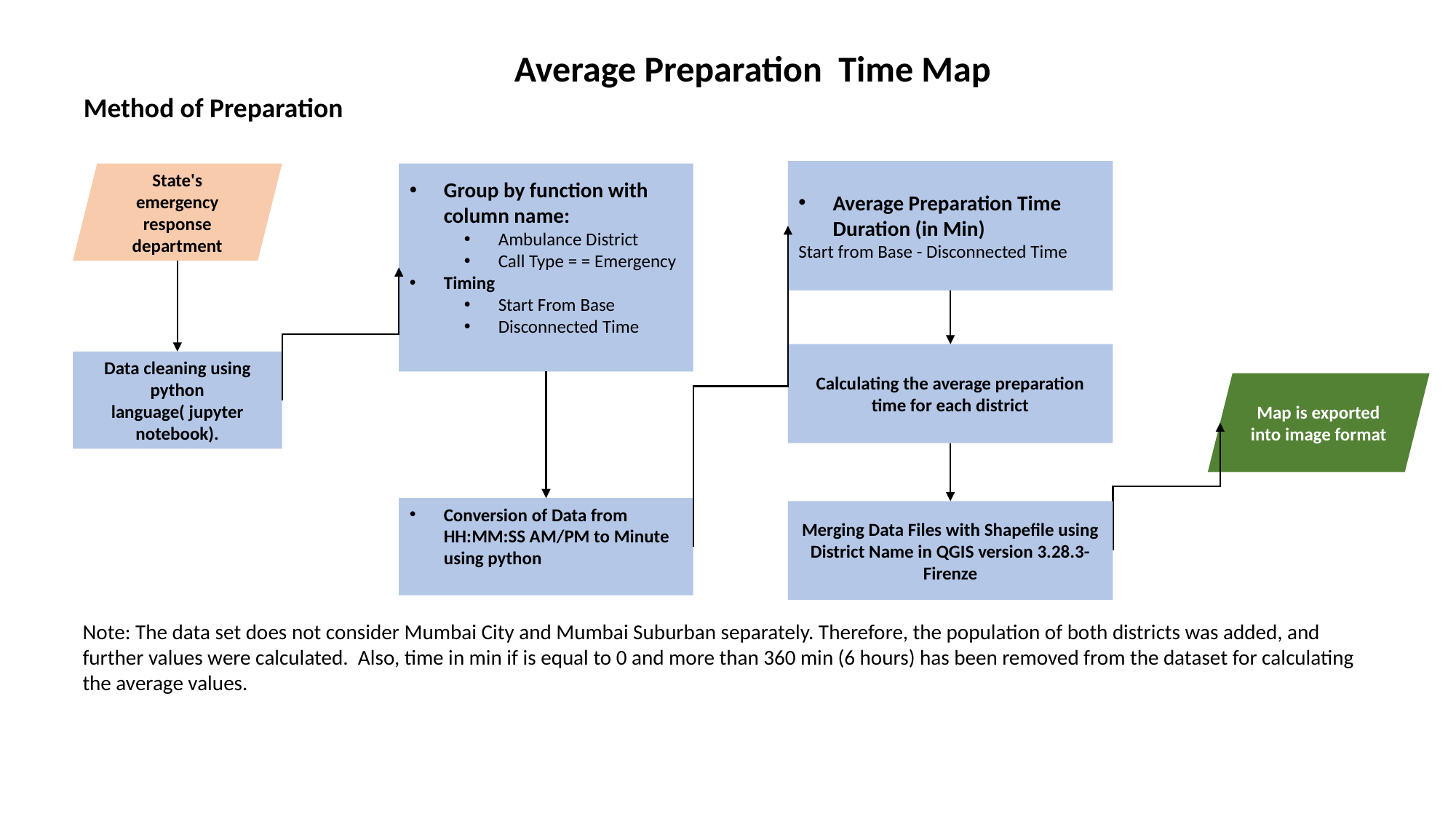

Average Preparation Time Map
Method of Preparation
Average Preparation Time Duration (in Min)
Start from Base - Disconnected Time
Group by function with column name:
Ambulance District
Call Type = = Emergency
Timing
Start From Base
Disconnected Time
State's emergency response department
Calculating the average preparation time for each district
Data cleaning using python language( jupyter notebook).
Map is exported into image format
Conversion of Data from HH:MM:SS AM/PM to Minute using python
Merging Data Files with Shapefile using District Name in QGIS version 3.28.3-Firenze
Note: The data set does not consider Mumbai City and Mumbai Suburban separately. Therefore, the population of both districts was added, and further values were calculated. Also, time in min if is equal to 0 and more than 360 min (6 hours) has been removed from the dataset for calculating the average values.

## Slide 10
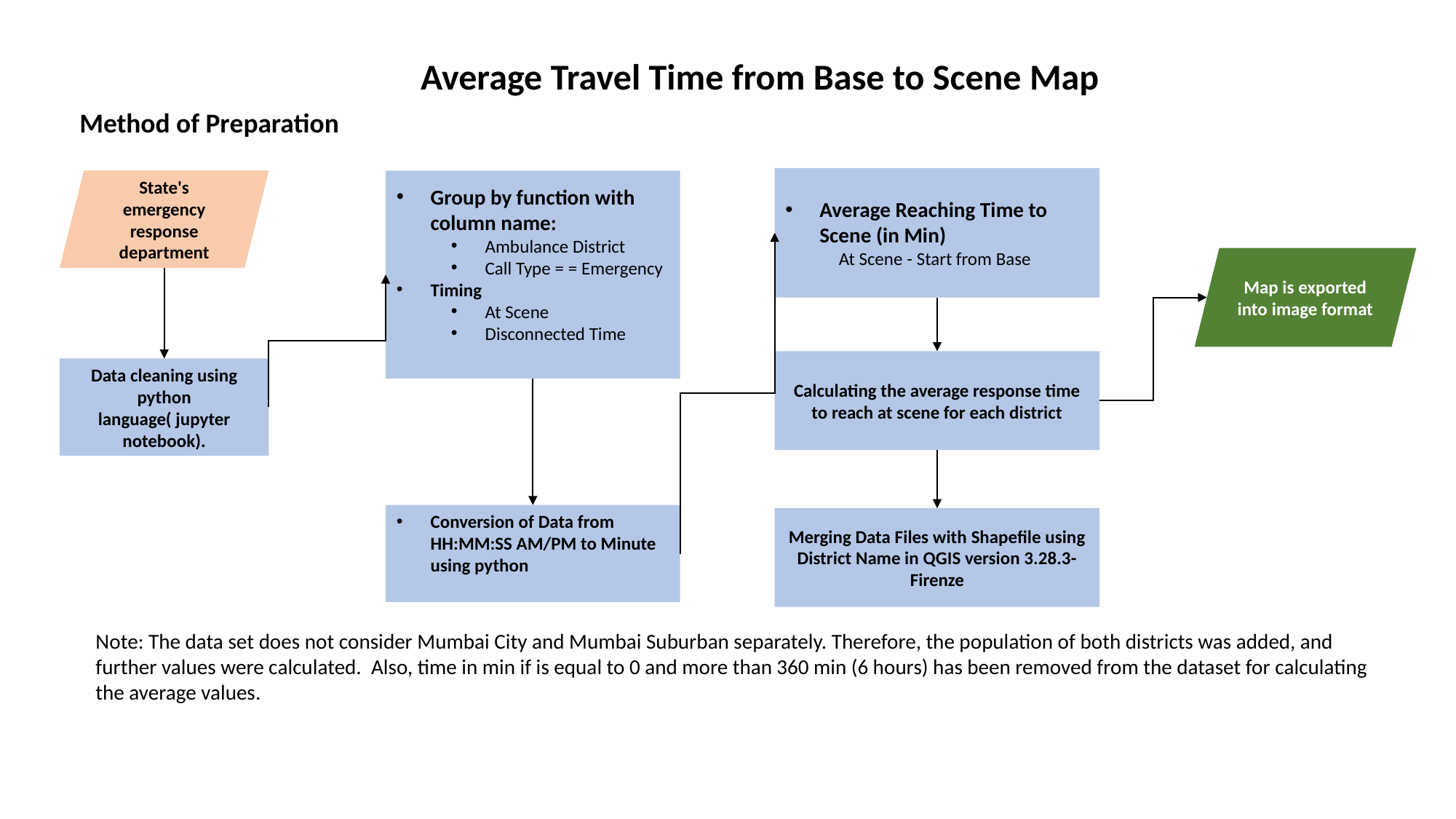

Average Travel Time from Base to Scene Map
Method of Preparation
Average Reaching Time to Scene (in Min)
At Scene - Start from Base
Group by function with column name:
Ambulance District
Call Type = = Emergency
Timing
At Scene
Disconnected Time
State's emergency response department
Map is exported into image format
Calculating the average response time to reach at scene for each district
Data cleaning using python language( jupyter notebook).
Conversion of Data from HH:MM:SS AM/PM to Minute using python
Merging Data Files with Shapefile using District Name in QGIS version 3.28.3-Firenze
Note: The data set does not consider Mumbai City and Mumbai Suburban separately. Therefore, the population of both districts was added, and further values were calculated. Also, time in min if is equal to 0 and more than 360 min (6 hours) has been removed from the dataset for calculating the average values.

## Slide 11
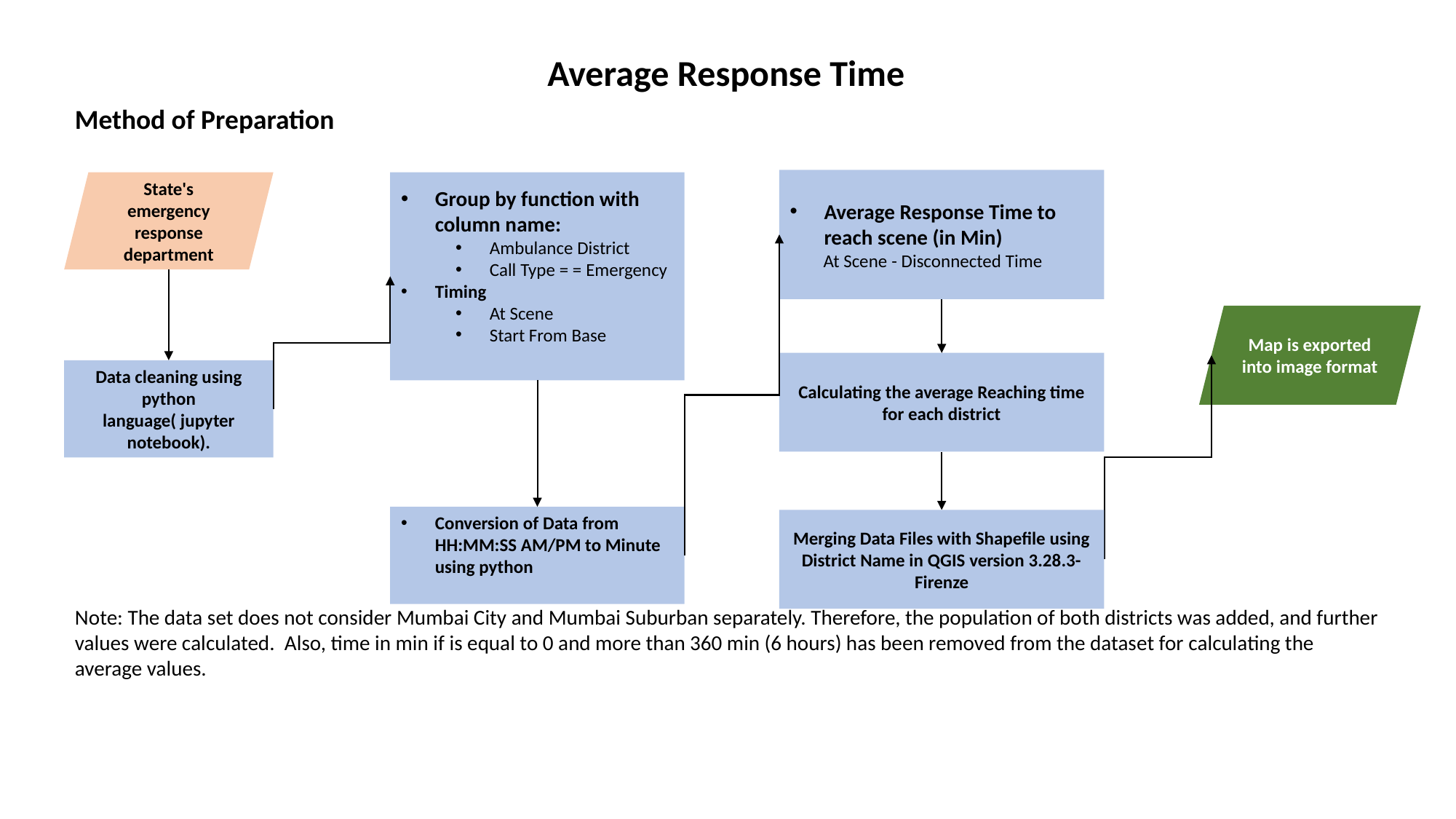

Average Response Time
Method of Preparation
Average Response Time to reach scene (in Min)
 At Scene - Disconnected Time
Group by function with column name:
Ambulance District
Call Type = = Emergency
Timing
At Scene
Start From Base
State's emergency response department
Map is exported into image format
Calculating the average Reaching time for each district
Data cleaning using python language( jupyter notebook).
Conversion of Data from HH:MM:SS AM/PM to Minute using python
Merging Data Files with Shapefile using District Name in QGIS version 3.28.3-Firenze
Note: The data set does not consider Mumbai City and Mumbai Suburban separately. Therefore, the population of both districts was added, and further values were calculated. Also, time in min if is equal to 0 and more than 360 min (6 hours) has been removed from the dataset for calculating the average values.

## Slide 12
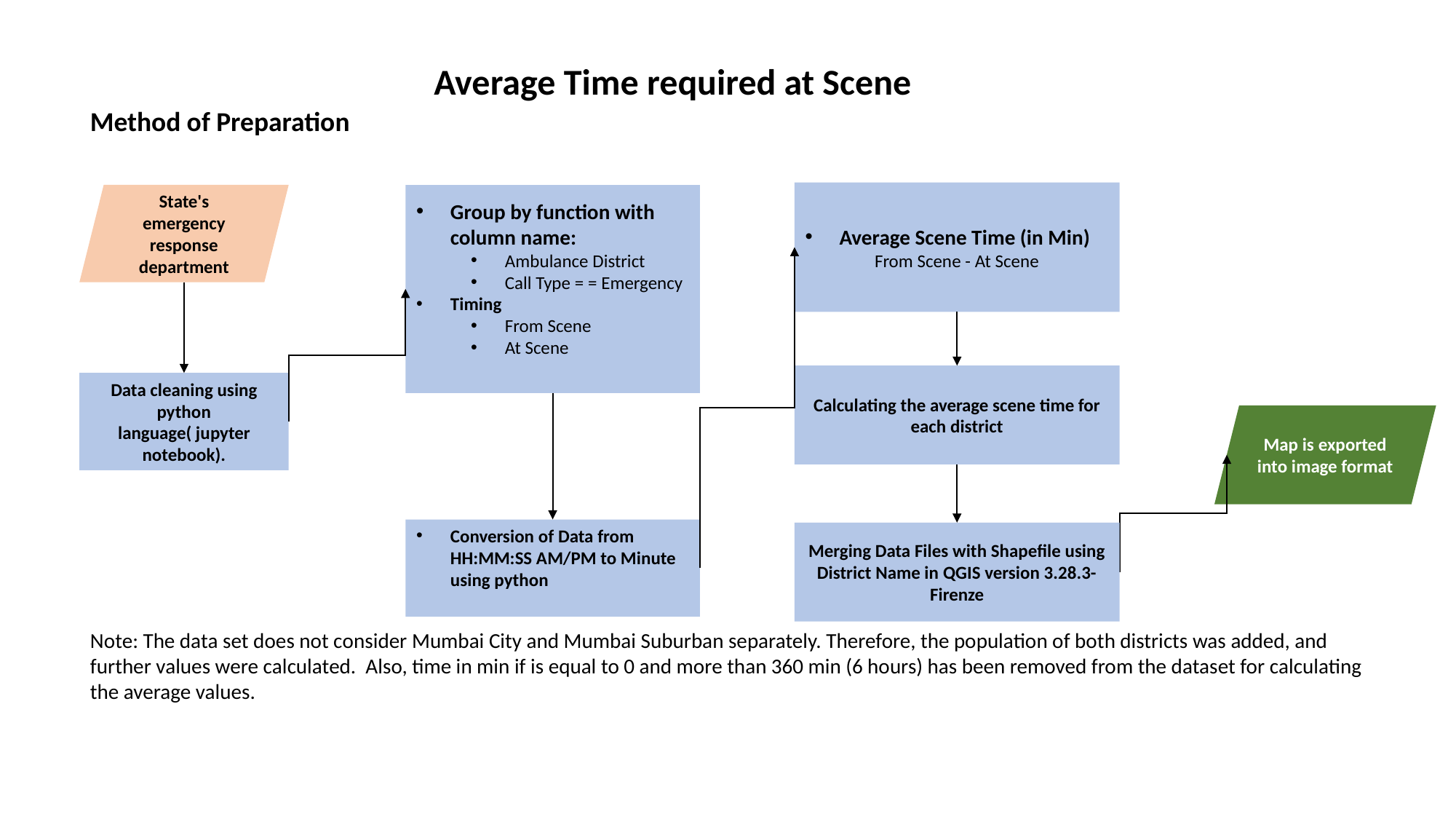

Average Time required at Scene
Method of Preparation
Average Scene Time (in Min)
From Scene - At Scene
Group by function with column name:
Ambulance District
Call Type = = Emergency
Timing
From Scene
At Scene
State's emergency response department
Calculating the average scene time for each district
Data cleaning using python language( jupyter notebook).
Map is exported into image format
Conversion of Data from HH:MM:SS AM/PM to Minute using python
Merging Data Files with Shapefile using District Name in QGIS version 3.28.3-Firenze
Note: The data set does not consider Mumbai City and Mumbai Suburban separately. Therefore, the population of both districts was added, and further values were calculated. Also, time in min if is equal to 0 and more than 360 min (6 hours) has been removed from the dataset for calculating the average values.

## Slide 13
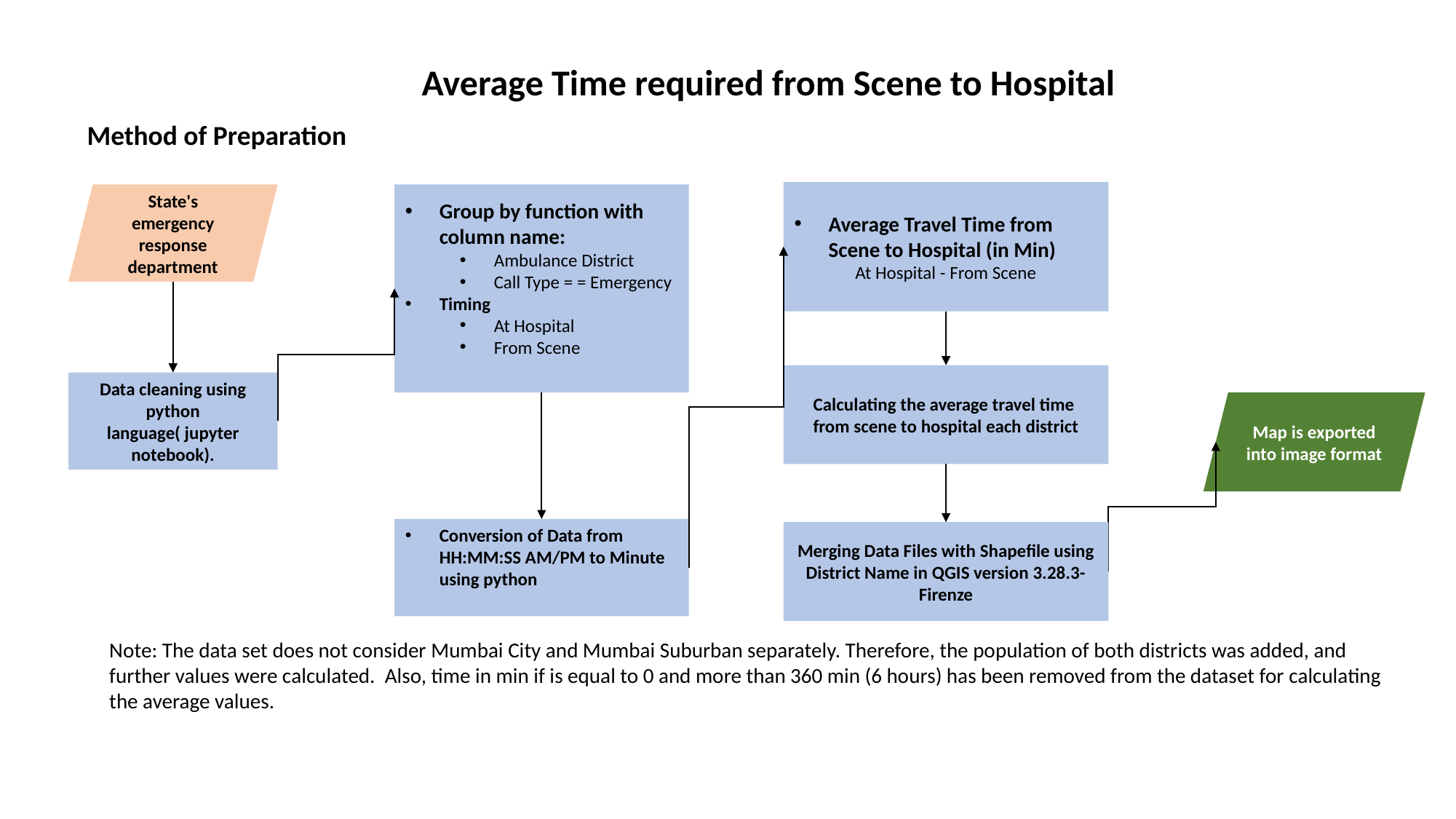

Average Time required from Scene to Hospital
Method of Preparation
Average Travel Time from Scene to Hospital (in Min)
At Hospital - From Scene
Group by function with column name:
Ambulance District
Call Type = = Emergency
Timing
At Hospital
From Scene
State's emergency response department
Calculating the average travel time from scene to hospital each district
Data cleaning using python language( jupyter notebook).
Map is exported into image format
Conversion of Data from HH:MM:SS AM/PM to Minute using python
Merging Data Files with Shapefile using District Name in QGIS version 3.28.3-Firenze
Note: The data set does not consider Mumbai City and Mumbai Suburban separately. Therefore, the population of both districts was added, and further values were calculated. Also, time in min if is equal to 0 and more than 360 min (6 hours) has been removed from the dataset for calculating the average values.

## Slide 14
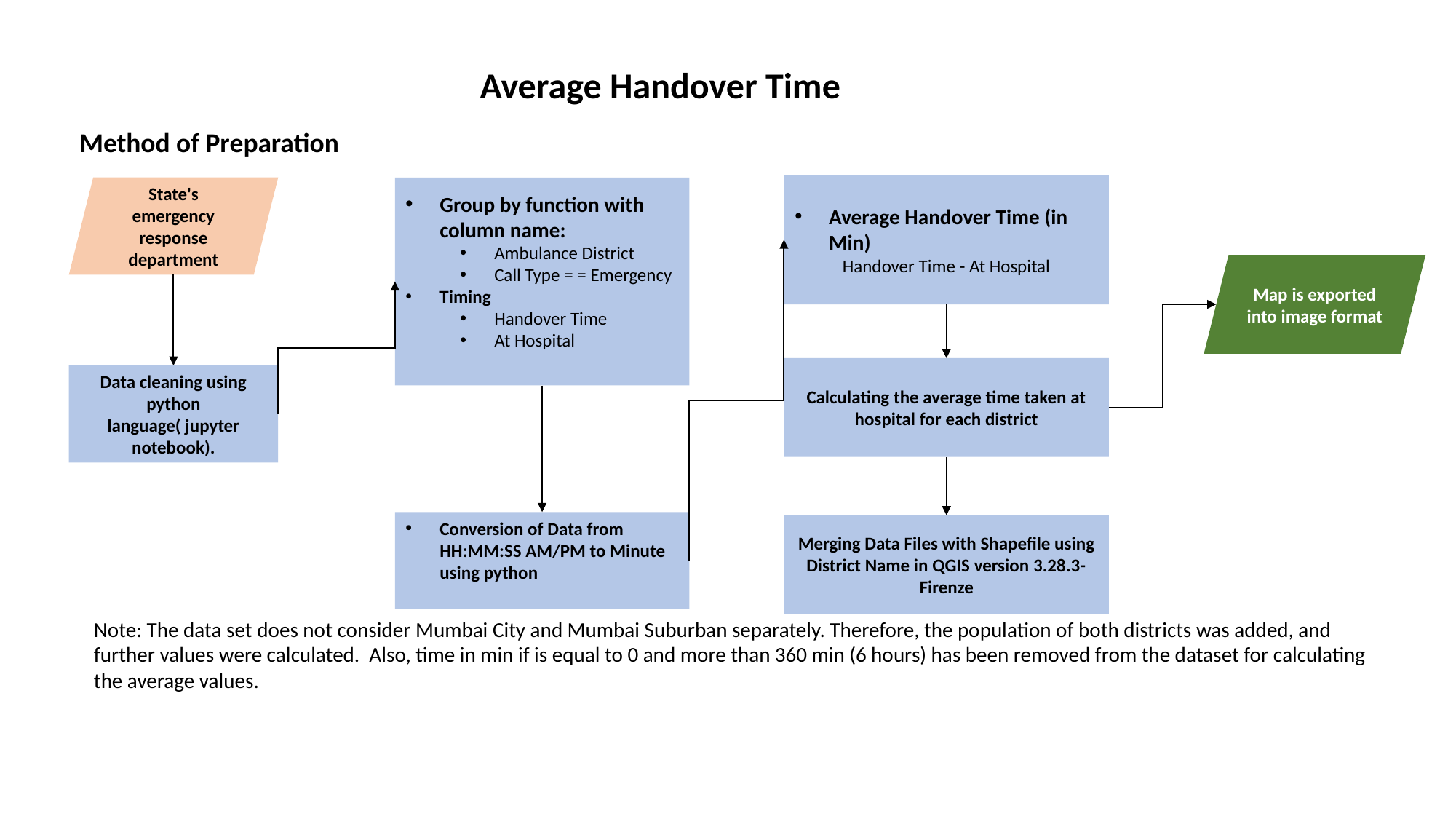

Average Handover Time
Method of Preparation
Average Handover Time (in Min)
Handover Time - At Hospital
Group by function with column name:
Ambulance District
Call Type = = Emergency
Timing
Handover Time
At Hospital
State's emergency response department
Map is exported into image format
Calculating the average time taken at hospital for each district
Data cleaning using python language( jupyter notebook).
Conversion of Data from HH:MM:SS AM/PM to Minute using python
Merging Data Files with Shapefile using District Name in QGIS version 3.28.3-Firenze
Note: The data set does not consider Mumbai City and Mumbai Suburban separately. Therefore, the population of both districts was added, and further values were calculated. Also, time in min if is equal to 0 and more than 360 min (6 hours) has been removed from the dataset for calculating the average values.

## Slide 15
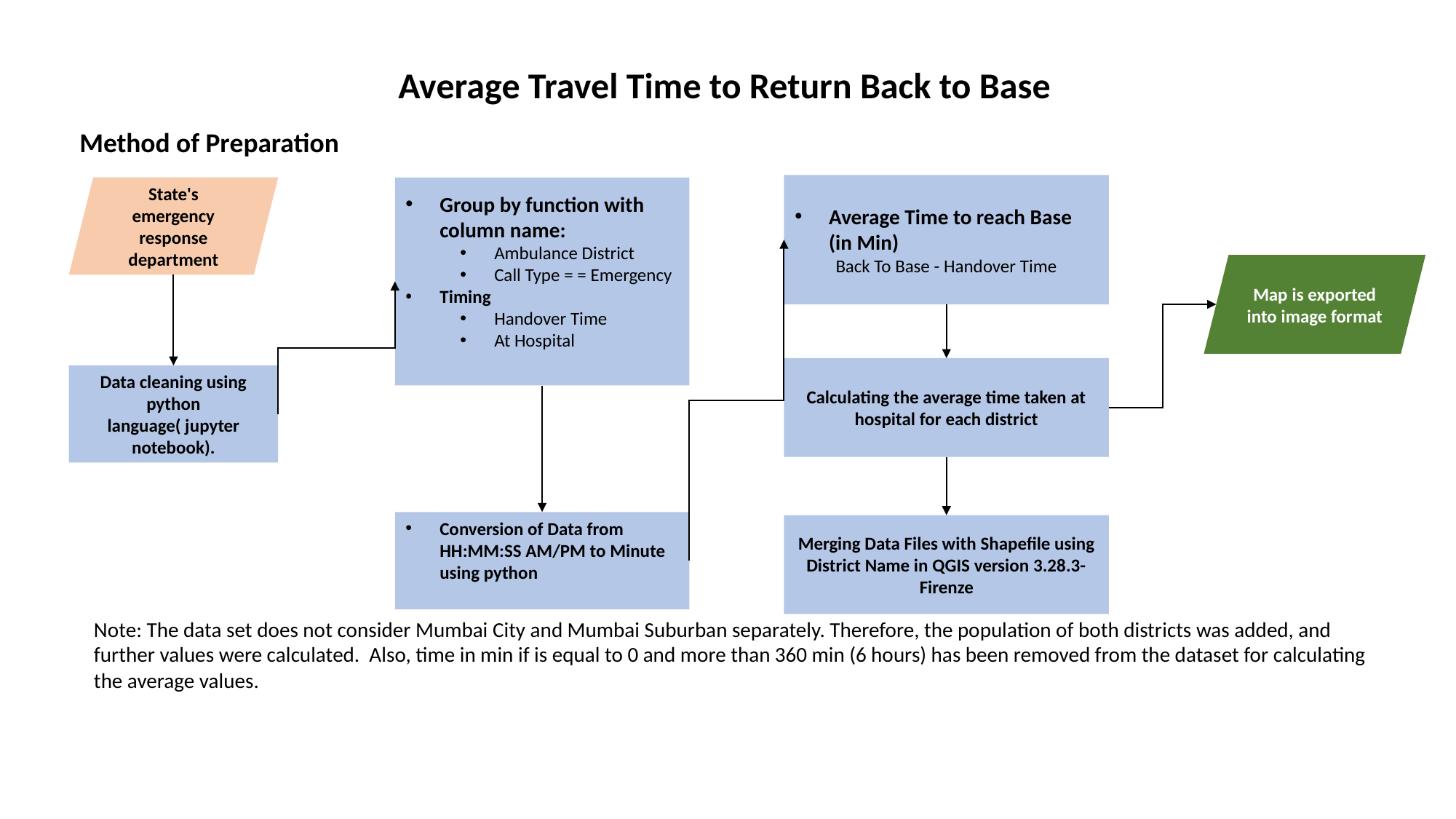

Average Travel Time to Return Back to Base
Method of Preparation
Average Time to reach Base (in Min)
Back To Base - Handover Time
Group by function with column name:
Ambulance District
Call Type = = Emergency
Timing
Handover Time
At Hospital
State's emergency response department
Map is exported into image format
Calculating the average time taken at hospital for each district
Data cleaning using python language( jupyter notebook).
Conversion of Data from HH:MM:SS AM/PM to Minute using python
Merging Data Files with Shapefile using District Name in QGIS version 3.28.3-Firenze
Note: The data set does not consider Mumbai City and Mumbai Suburban separately. Therefore, the population of both districts was added, and further values were calculated. Also, time in min if is equal to 0 and more than 360 min (6 hours) has been removed from the dataset for calculating the average values.

## Slide 16
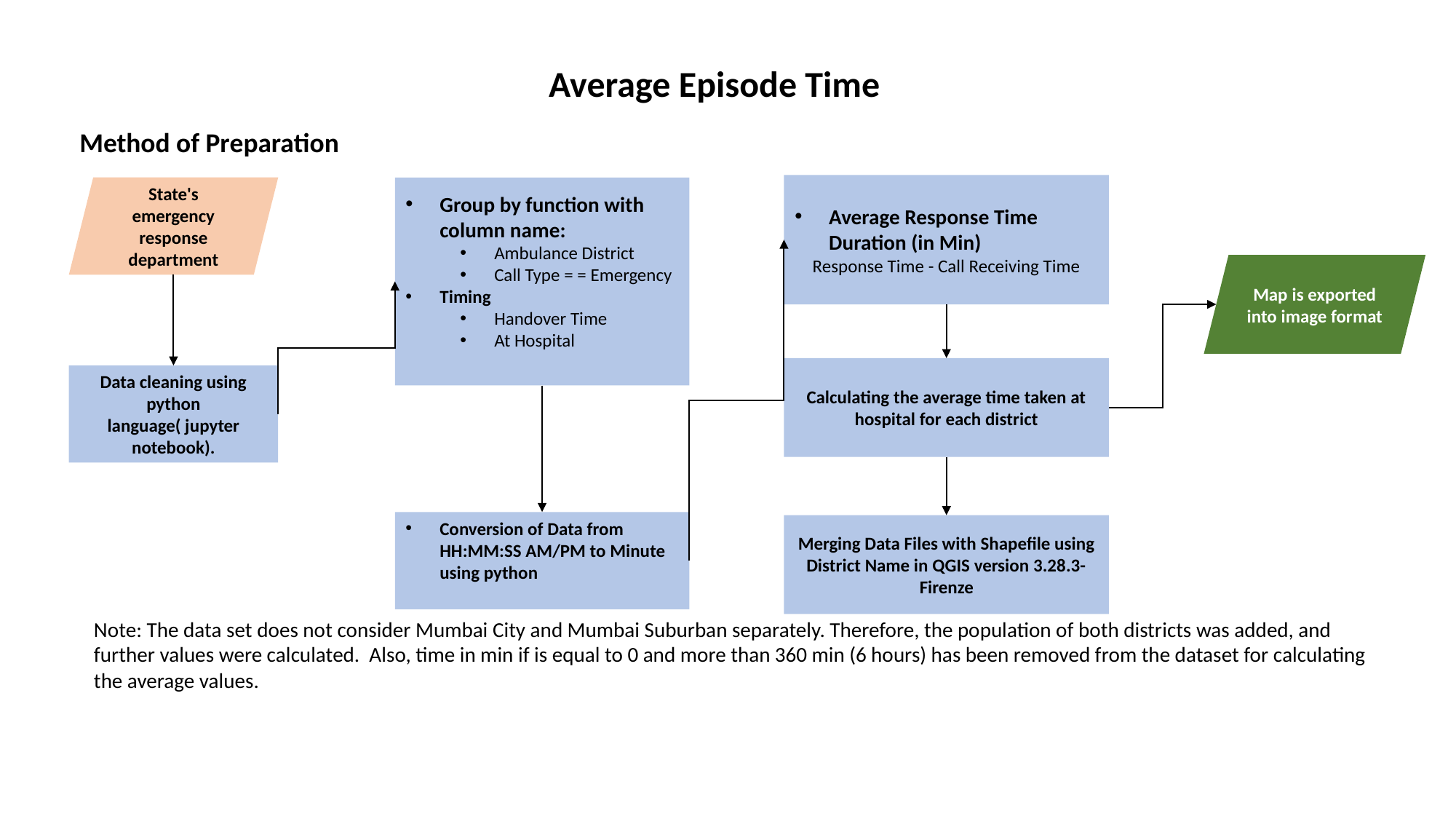

Average Episode Time
Method of Preparation
Average Response Time Duration (in Min)
Response Time - Call Receiving Time
Group by function with column name:
Ambulance District
Call Type = = Emergency
Timing
Handover Time
At Hospital
State's emergency response department
Map is exported into image format
Calculating the average time taken at hospital for each district
Data cleaning using python language( jupyter notebook).
Conversion of Data from HH:MM:SS AM/PM to Minute using python
Merging Data Files with Shapefile using District Name in QGIS version 3.28.3-Firenze
Note: The data set does not consider Mumbai City and Mumbai Suburban separately. Therefore, the population of both districts was added, and further values were calculated. Also, time in min if is equal to 0 and more than 360 min (6 hours) has been removed from the dataset for calculating the average values.

## Slide 17
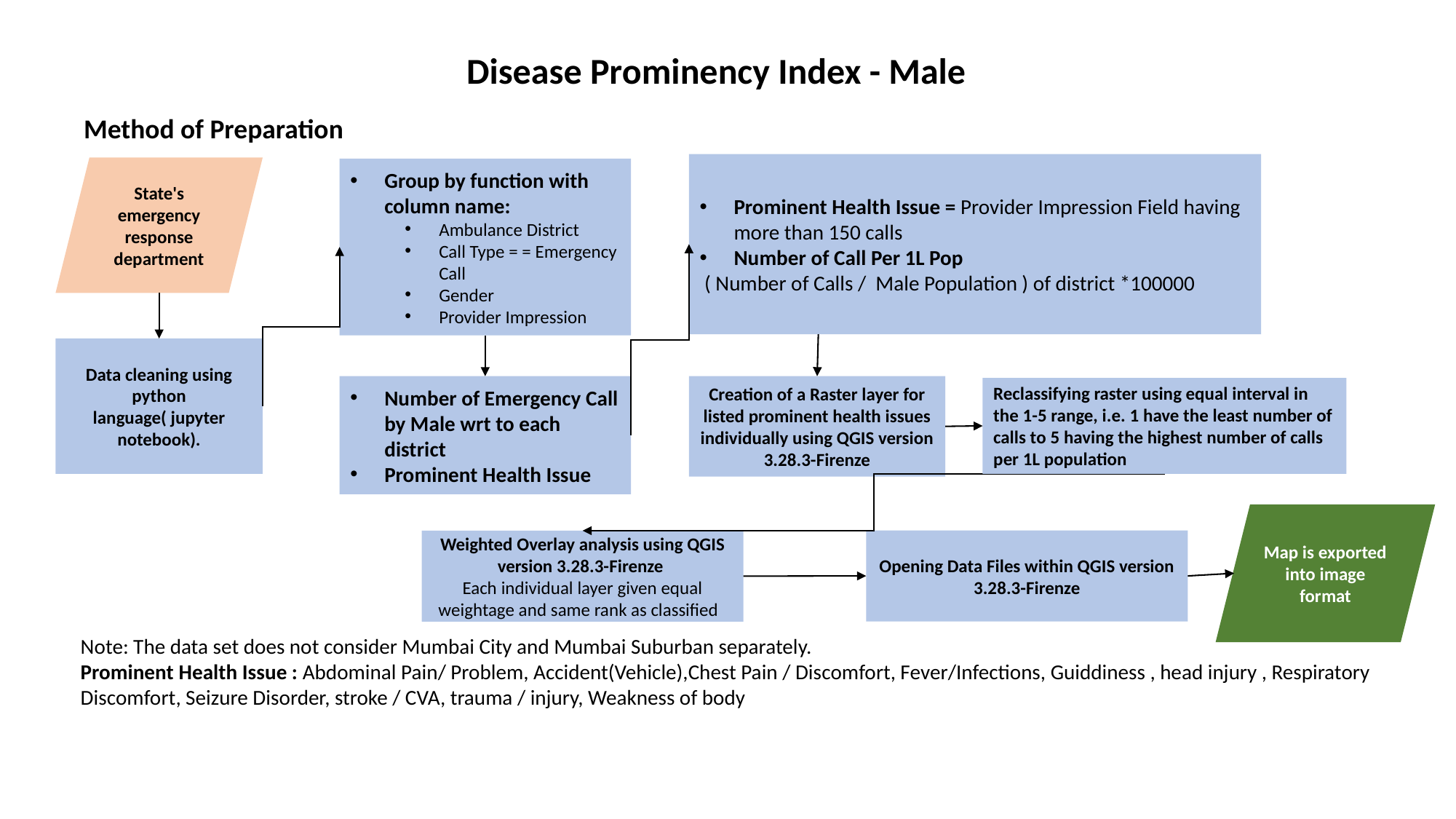

Disease Prominency Index - Male
Method of Preparation
Prominent Health Issue = Provider Impression Field having more than 150 calls
Number of Call Per 1L Pop
 ( Number of Calls / Male Population ) of district *100000
State's emergency response department
Group by function with column name:
Ambulance District
Call Type = = Emergency Call
Gender
Provider Impression
Data cleaning using python language( jupyter notebook).
Creation of a Raster layer for listed prominent health issues individually using QGIS version 3.28.3-Firenze
Number of Emergency Call by Male wrt to each district
Prominent Health Issue
Reclassifying raster using equal interval in the 1-5 range, i.e. 1 have the least number of calls to 5 having the highest number of calls per 1L population
Map is exported into image format
Opening Data Files within QGIS version 3.28.3-Firenze
Weighted Overlay analysis using QGIS version 3.28.3-Firenze
Each individual layer given equal weightage and same rank as classified
Note: The data set does not consider Mumbai City and Mumbai Suburban separately.
Prominent Health Issue : Abdominal Pain/ Problem, Accident(Vehicle),Chest Pain / Discomfort, Fever/Infections, Guiddiness , head injury , Respiratory Discomfort, Seizure Disorder, stroke / CVA, trauma / injury, Weakness of body

## Slide 18
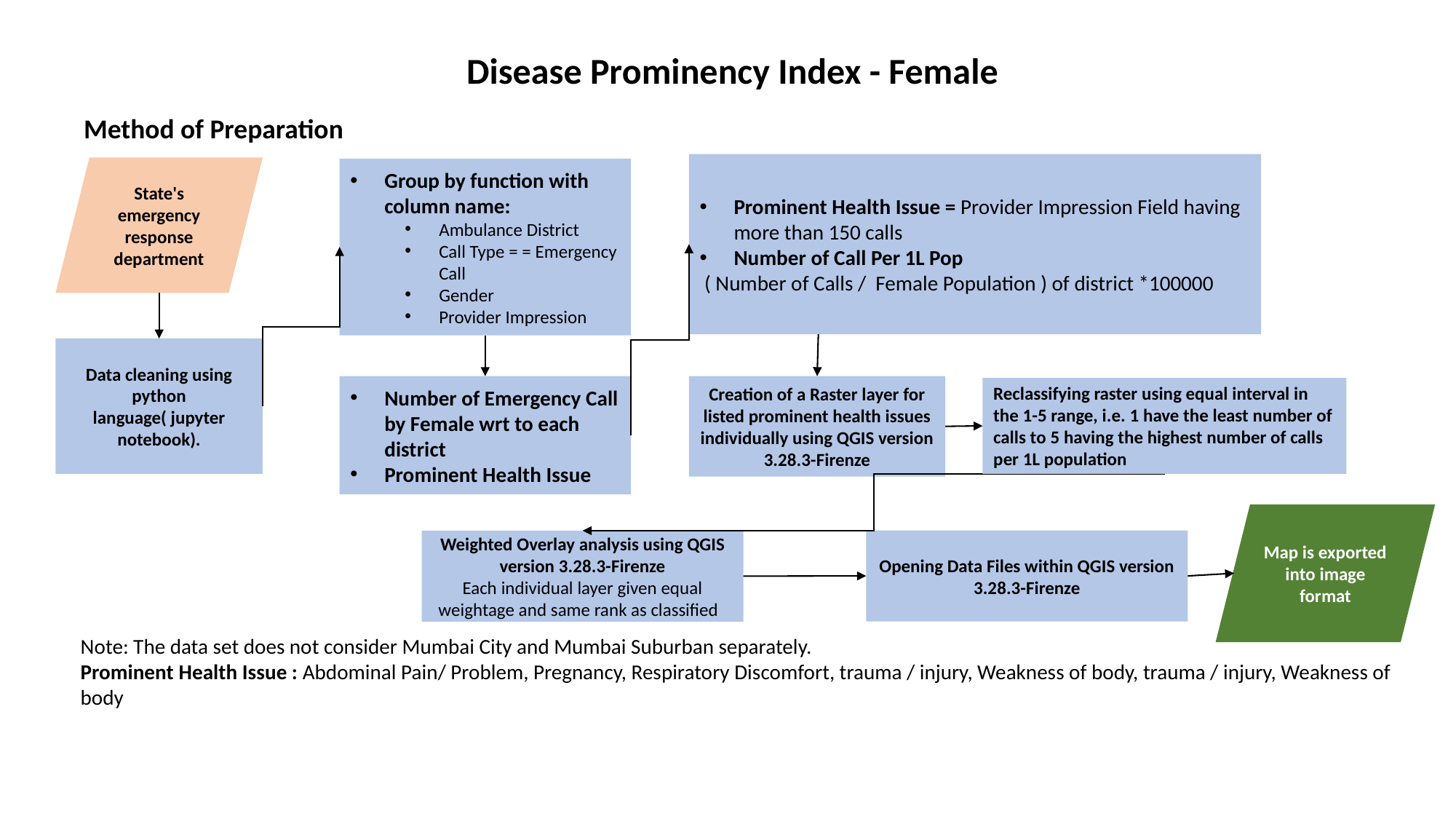

Disease Prominency Index - Female
Method of Preparation
Prominent Health Issue = Provider Impression Field having more than 150 calls
Number of Call Per 1L Pop
 ( Number of Calls / Female Population ) of district *100000
State's emergency response department
Group by function with column name:
Ambulance District
Call Type = = Emergency Call
Gender
Provider Impression
Data cleaning using python language( jupyter notebook).
Creation of a Raster layer for listed prominent health issues individually using QGIS version 3.28.3-Firenze
Number of Emergency Call by Female wrt to each district
Prominent Health Issue
Reclassifying raster using equal interval in the 1-5 range, i.e. 1 have the least number of calls to 5 having the highest number of calls per 1L population
Map is exported into image format
Opening Data Files within QGIS version 3.28.3-Firenze
Weighted Overlay analysis using QGIS version 3.28.3-Firenze
Each individual layer given equal weightage and same rank as classified
Note: The data set does not consider Mumbai City and Mumbai Suburban separately.
Prominent Health Issue : Abdominal Pain/ Problem, Pregnancy, Respiratory Discomfort, trauma / injury, Weakness of body, trauma / injury, Weakness of body
