# Supplementary material for: Examining district-level disparity and determinants of timeliness of emergency medical services in Maharashtra, India
Source: Sci Rep. 2023 Dec 1;13:21239. doi: 10.1038/s41598-023-48713-1 (PMC10692338; doi:10.1038/s41598-023-48713-1)
Supplement: Supplementary file 3 — Supplementary Information 3. [file 41598_2023_48713_MOESM3_ESM.docx]

**Appendix III**

**District wise MEMS call details with respect to disease type – November 2022**

| Appendix III: Table 1: District wise MEMS call details with respect to disease type – November 2022 \| Emergency Call | | | | | | | | |
| --- | --- | --- | --- | --- | --- | --- | --- | --- |
| District | **Pregnancy** | **Trauma / injury** | **Abdominal Pain/ Problem** | **Respiratory Discomfort** | **Weakness of body** | **Head injury** | **Other** | **Total** |
| Ahmednagar | 750 | 359 | 178 | 81 | 74 | 42 | 610 | 2094 |
| Akola | 373 | 106 | 62 | 40 | 17 | 27 | 228 | 853 |
| Amravati | 540 | 373 | 454 | 131 | 147 | 87 | 839 | 2571 |
| Aurangabad | 492 | 198 | 82 | 56 | 20 | 64 | 313 | 1225 |
| Beed | 583 | 162 | 63 | 52 | 27 | 45 | 292 | 1224 |
| Bhandara | 241 | 51 | 93 | 49 | 43 | 32 | 130 | 639 |
| Buldhana | 447 | 208 | 77 | 57 | 35 | 51 | 329 | 1204 |
| Chandrapur | 684 | 250 | 199 | 126 | 100 | 107 | 506 | 1972 |
| Dhule | 630 | 302 | 79 | 27 | 50 | 74 | 271 | 1433 |
| Gadchiroli | 197 | 83 | 82 | 76 | 46 | 42 | 156 | 682 |
| Gondia | 223 | 181 | 111 | 59 | 84 | 40 | 216 | 914 |
| Hingoli | 404 | 86 | 24 | 23 | 12 | 48 | 124 | 721 |
| Jalgaon | 655 | 299 | 111 | 66 | 44 | 77 | 419 | 1671 |
| Jalna | 296 | 101 | 56 | 51 | 25 | 44 | 186 | 759 |
| Kolhapur | 912 | 435 | 213 | 155 | 115 | 89 | 732 | 2651 |
| Latur | 386 | 174 | 82 | 60 | 21 | 59 | 293 | 1075 |
| Mumbai | 359 | 483 | 291 | 361 | 272 | 114 | 1064 | 2944 |
| Nagpur | 546 | 270 | 213 | 110 | 94 | 52 | 447 | 1732 |
| Nanded | 287 | 261 | 86 | 84 | 19 | 73 | 413 | 1223 |
| Nandurbar | 338 | 177 | 66 | 34 | 36 | 59 | 201 | 911 |
| Nashik | 1655 | 477 | 212 | 155 | 114 | 130 | 963 | 3706 |
| Osmanabad | 200 | 128 | 34 | 30 | 27 | 48 | 313 | 780 |
| Palghar | 643 | 232 | 138 | 89 | 65 | 41 | 409 | 1617 |
| Parbhani | 276 | 98 | 42 | 31 | 10 | 34 | 175 | 666 |
| Pune | 1576 | 692 | 358 | 326 | 272 | 109 | 1259 | 4592 |
| Raigad | 170 | 80 | 57 | 50 | 36 | 22 | 168 | 583 |
| Ratnagiri | 167 | 109 | 72 | 64 | 32 | 16 | 294 | 754 |
| Sangli | 450 | 358 | 135 | 101 | 78 | 110 | 442 | 1674 |
| Satara | 924 | 423 | 196 | 109 | 96 | 80 | 598 | 2426 |
| Sindhudurg | 100 | 118 | 95 | 72 | 45 | 55 | 349 | 834 |
| Solapur | 1032 | 436 | 236 | 91 | 90 | 81 | 659 | 2625 |
| Thane | 490 | 254 | 179 | 98 | 102 | 54 | 446 | 1623 |
| Wardha | 111 | 91 | 47 | 39 | 26 | 29 | 166 | 509 |
| Washim | 277 | 86 | 63 | 43 | 12 | 30 | 137 | 648 |
| Yavatmal | 290 | 222 | 129 | 69 | 53 | 72 | 375 | 1210 |
| Note: Mumbai and Mumbai Suburban has been considered together | | | | | | | | |

| Appendix III: Table 2: District wise MEMS call details with respect to disease type – November 2022  \| Hospital-to-hospital Transfer Call | | | | | | | | |
| --- | --- | --- | --- | --- | --- | --- | --- | --- |
| District | **Pregnancy** | **Trauma / injury** | **Respiratory Discomfort** | **Abdominal Pain/ Problem** | **Poisoning** | **Chest Pain / Discomfort** | **Other** | **Total** |
| Ahmednagar | 217 | 46 | 48 | 39 | 26 | 11 | 251 | 638 |
| Akola | 134 | 33 | 31 | 12 | 30 | 12 | 128 | 380 |
| Amravati | 125 | 151 | 93 | 114 | 62 | 48 | 391 | 984 |
| Aurangabad | 152 | 51 | 36 | 24 | 33 | 22 | 128 | 446 |
| Beed | 165 | 59 | 40 | 28 | 24 | 11 | 132 | 459 |
| Bhandara | 69 | 32 | 22 | 36 | 10 | 6 | 100 | 275 |
| Buldhana | 184 | 60 | 47 | 45 | 60 | 16 | 186 | 598 |
| Chandrapur | 297 | 112 | 91 | 120 | 55 | 55 | 325 | 1055 |
| Dhule | 213 | 73 | 24 | 29 | 19 | 13 | 168 | 539 |
| Gadchiroli | 106 | 43 | 40 | 43 | 9 | 18 | 136 | 395 |
| Gondia | 68 | 48 | 26 | 30 | 8 | 8 | 96 | 284 |
| Hingoli | 98 | 42 | 28 | 12 | 21 | 10 | 80 | 291 |
| Jalgaon | 247 | 74 | 27 | 45 | 78 | 10 | 231 | 712 |
| Jalna | 114 | 49 | 29 | 26 | 32 | 12 | 115 | 377 |
| Kolhapur | 391 | 75 | 81 | 89 | 43 | 48 | 384 | 1111 |
| Latur | 119 | 71 | 35 | 29 | 25 | 20 | 159 | 458 |
| Mumbai | 372 | 22 | 107 | 108 | 7 | 25 | 271 | 912 |
| Nagpur | 256 | 82 | 46 | 78 | 28 | 46 | 230 | 766 |
| Nanded | 68 | 101 | 76 | 37 | 76 | 70 | 190 | 618 |
| Nandurbar | 145 | 47 | 19 | 29 | 14 | 5 | 180 | 439 |
| Nashik | 544 | 147 | 109 | 87 | 46 | 44 | 462 | 1439 |
| Osmanabad | 68 | 45 | 36 | 31 | 17 | 19 | 171 | 387 |
| Palghar | 203 | 71 | 47 | 43 | 8 | 13 | 214 | 599 |
| Parbhani | 138 | 44 | 14 | 22 | 20 | 14 | 95 | 347 |
| Pune | 807 | 138 | 161 | 138 | 29 | 68 | 532 | 1873 |
| Raigad | 128 | 42 | 26 | 33 | 8 | 12 | 168 | 417 |
| Ratnagiri | 98 | 21 | 28 | 28 | 14 | 18 | 155 | 362 |
| Sangli | 194 | 42 | 44 | 33 | 22 | 20 | 168 | 523 |
| Satara | 291 | 65 | 59 | 54 | 28 | 25 | 265 | 787 |
| Sindhudurg | 63 | 40 | 47 | 32 | 8 | 26 | 261 | 477 |
| Solapur | 320 | 76 | 53 | 50 | 17 | 18 | 231 | 765 |
| Thane | 300 | 109 | 81 | 77 | 20 | 32 | 336 | 955 |
| Wardha | 15 | 35 | 26 | 10 | 15 | 15 | 104 | 220 |
| Washim | 129 | 36 | 23 | 28 | 20 | 7 | 78 | 321 |
| Yavatmal | 96 | 91 | 46 | 57 | 65 | 24 | 221 | 600 |
| Note: Mumbai and Mumbai Suburban has been considered together | | | | | | | | |


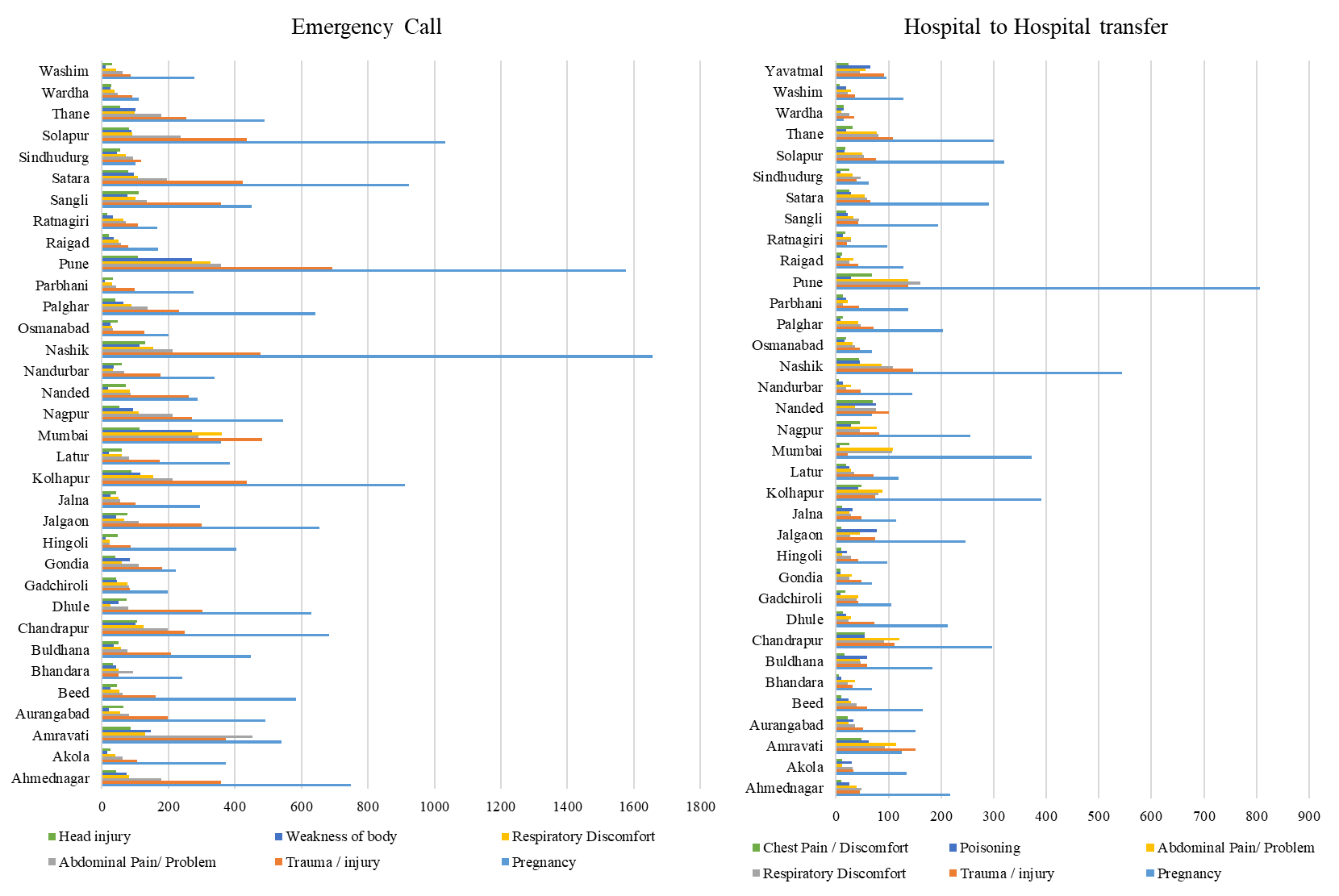


Appendix III: Figure 1: District wise MEMS call details with respect to disease type – November 2022 (Authors’ computation).

**District-wise distribution of Male and Female Health Issues per 1,00,000 population for Emergency Calls**


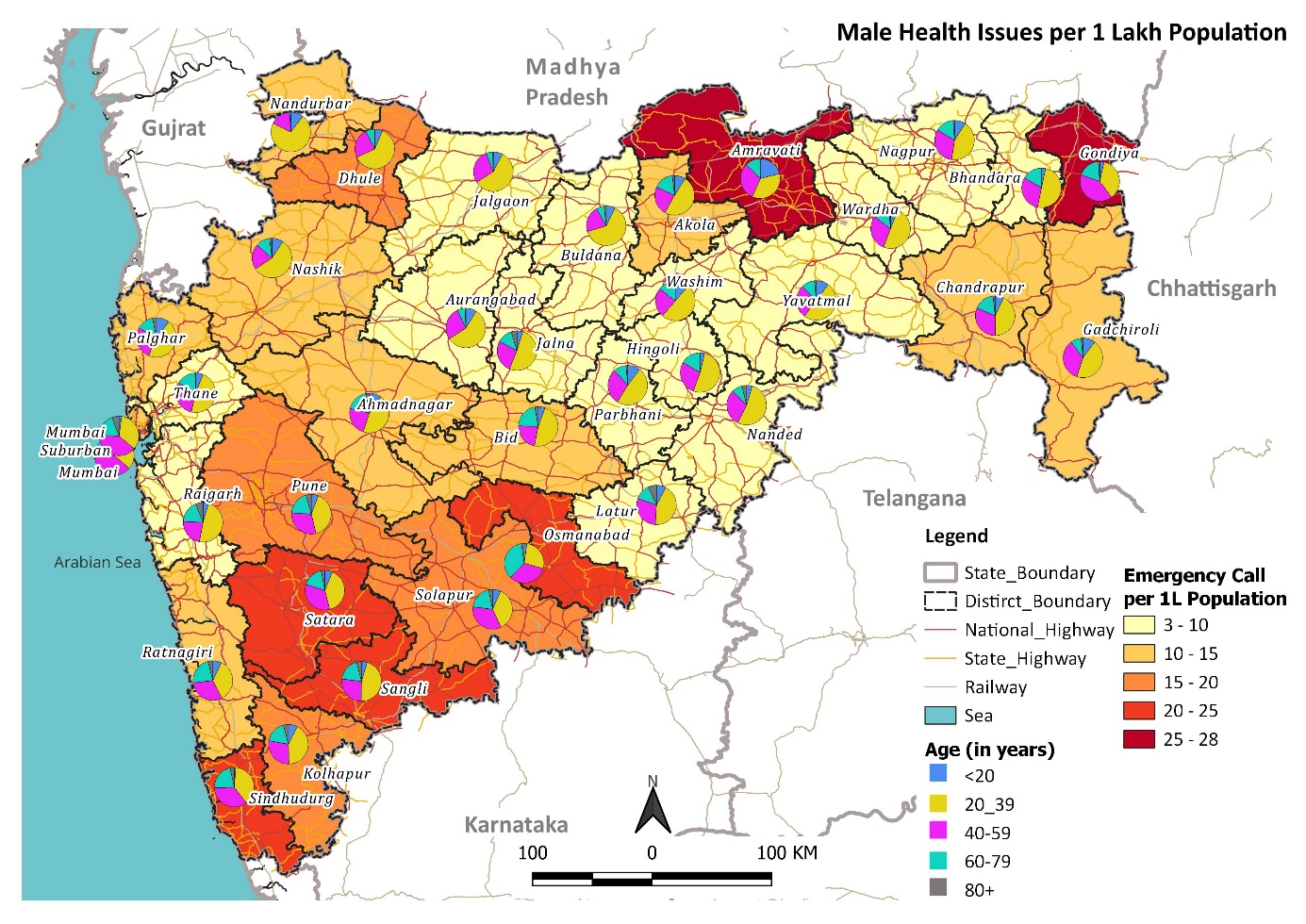


Appendix III: Figure 2: District-wise distribution of Male Health Issues per 1,00,000 population for Emergency Calls. QGIS Geographic Information System v3.28.3-Firenze. QGIS.org, 2023. QGIS Association. http://www.qgis.org.


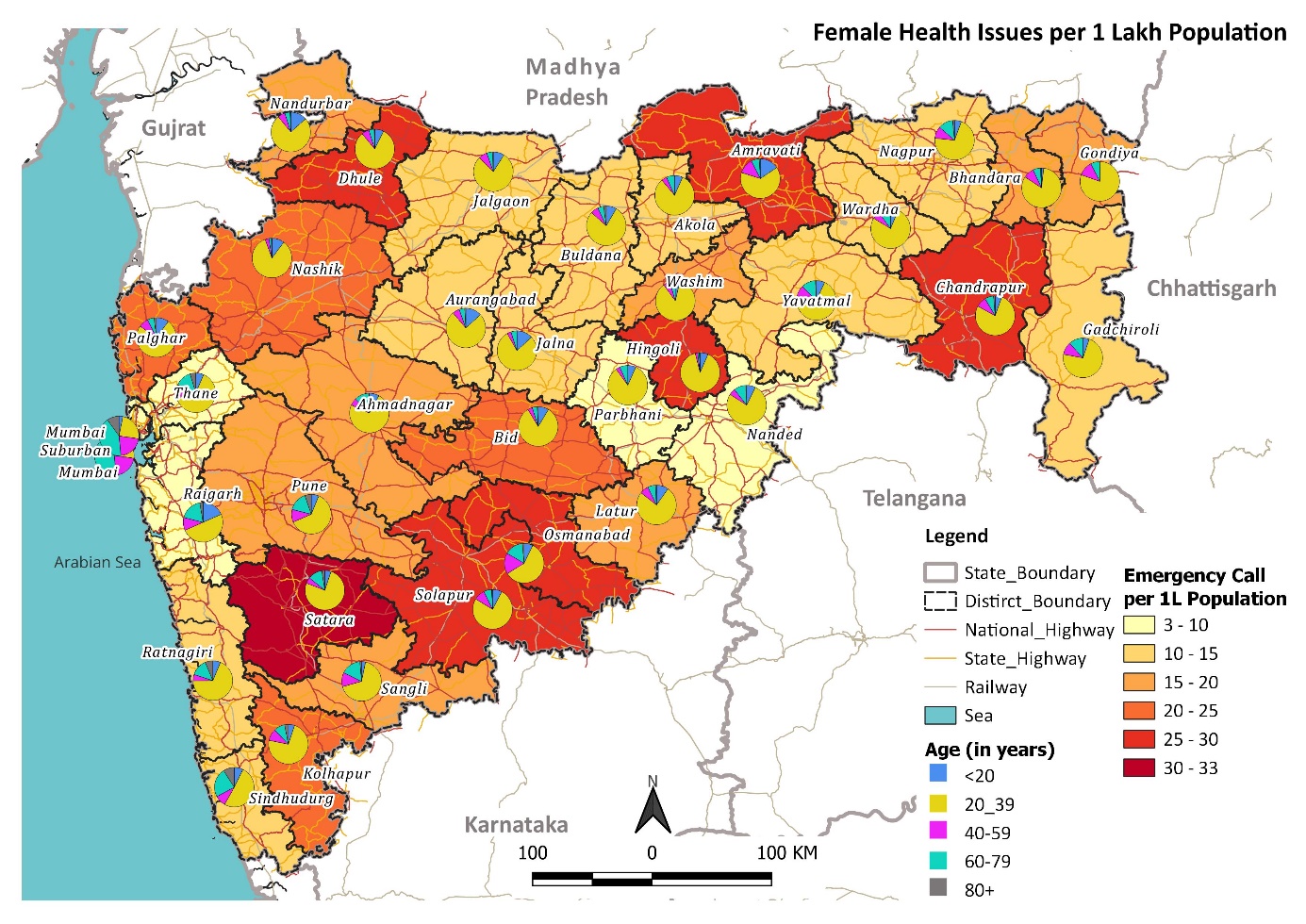


Appendix III: Figure 3: District-wise distribution of Female Health Issues per 1,00,000 population for Emergency Calls. QGIS Geographic Information System v3.28.3-Firenze. QGIS.org, 2023. QGIS Association. http://www.qgis.org.

**District-wise distribution of Disease Prominency Index for males and females per 1,00,000 Population for Emergency Calls**

Note:

Prominent health issues were assessed by taking into account the number of calls received. Health concerns that involve more than 150 calls and 250 calls are considered significant for emergency and hospital-to-hospital transfer calls, respectively.

***For Emergency ( No of calls >=150 are considered)***

For male

Abdominal Pain/ Problem, Accident(Vehicle),Chest Pain / Discomfort, Fever/Infections, Guiddiness , head injury , Respiratory Discomfort, Seizure Disorder, stroke / CVA, trauma / injury, Weakness of body

For female

Abdominal Pain/ Problem, Pregnancy, Respiratory Discomfort, trauma / injury, Weakness of body, trauma / injury, Weakness of body

***For Hospital to Hospital calls ( No of calls >=250 are considered)***

For male

Abdominal Pain/ Problem, Assault, Chest Pain / Discomfort, Fever/Infections, head injury, Poisoning, Respiratory Discomfort, Seizure Disorder, Snake Bite, Stings / Venomous bites, stroke / CVA

For female

Abdominal Pain/ Problem, Chest Pain / Discomfort, Fever/Infections, Poisoning, Pregnancy, Respiratory Discomfort, trauma / injury, Weakness of body


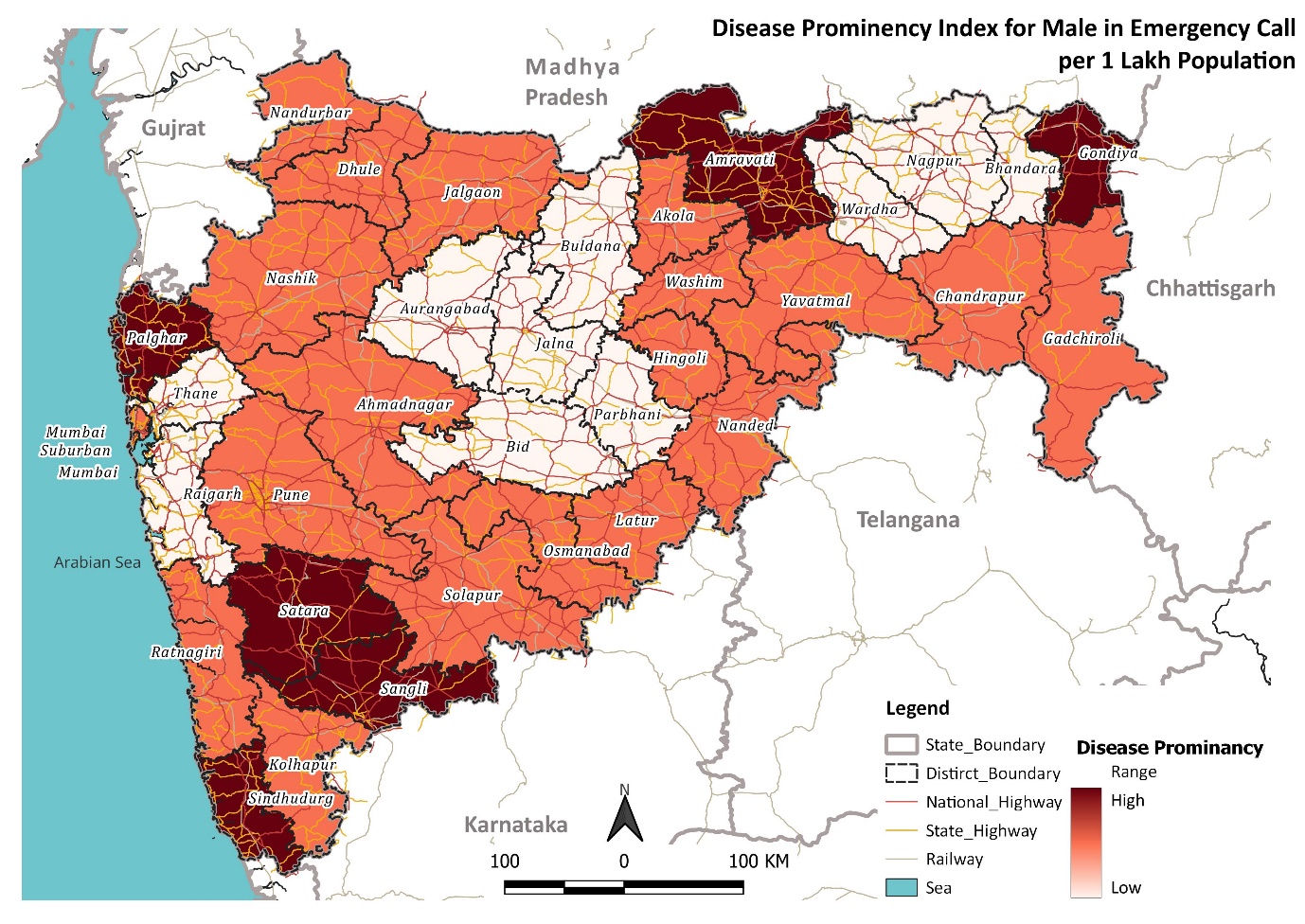


Appendix III: Figure 4: District-wise distribution of Disease Prominency Index for males per 1,00,000 Population for Emergency Calls. QGIS Geographic Information System v3.28.3-Firenze. QGIS.org, 2023. QGIS Association. http://www.qgis.org.


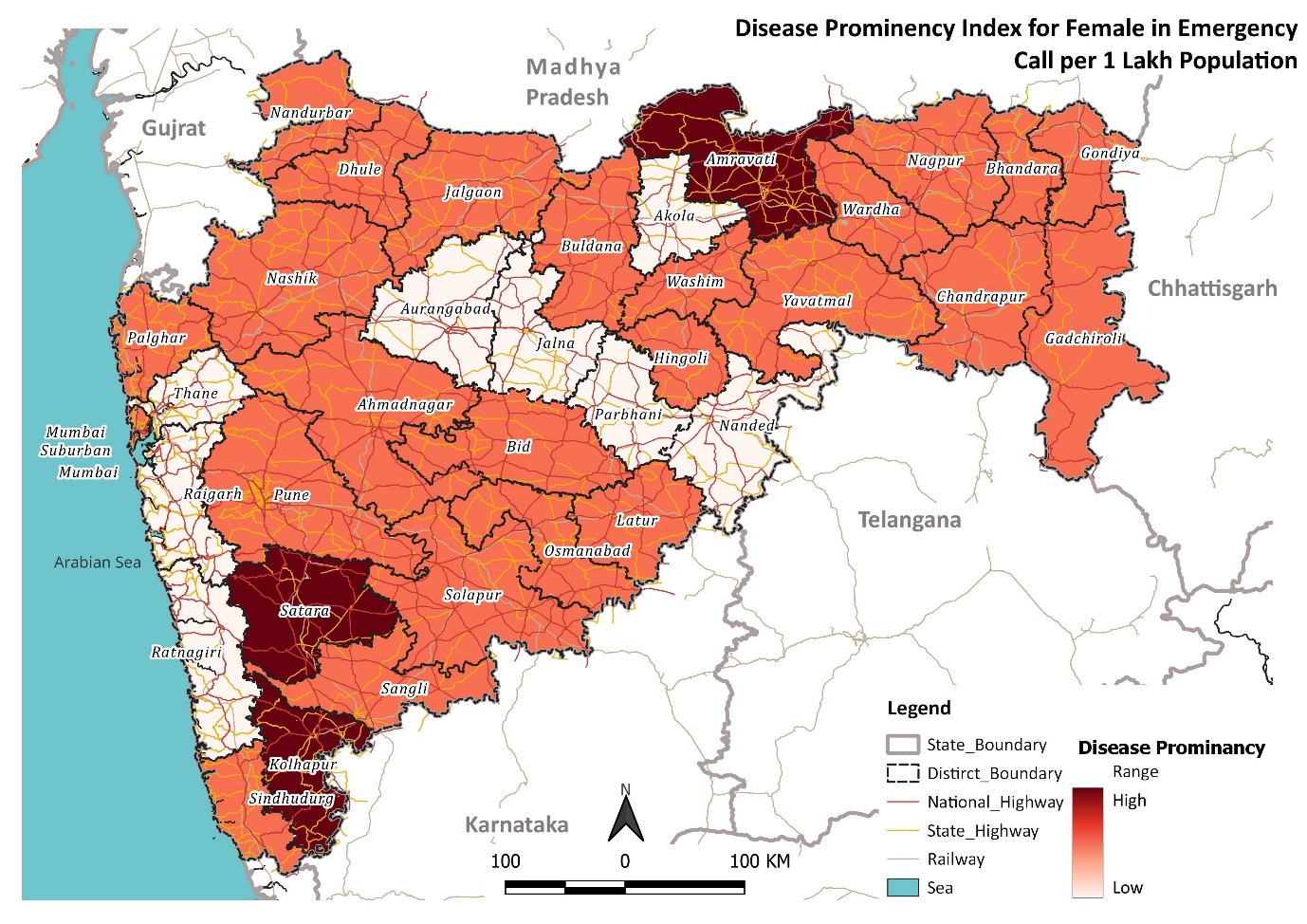


Appendix III: Figure 5: District-wise distribution of Disease Prominency Index for females per 1,00,000 Population for Emergency Calls. QGIS Geographic Information System v3.28.3-Firenze. QGIS.org, 2023. QGIS Association. http://www.qgis.org.

**District-wise distribution of Male and Female Health Issues per 1,00,000 population for Hospital-to-Hospital Transfer Calls**


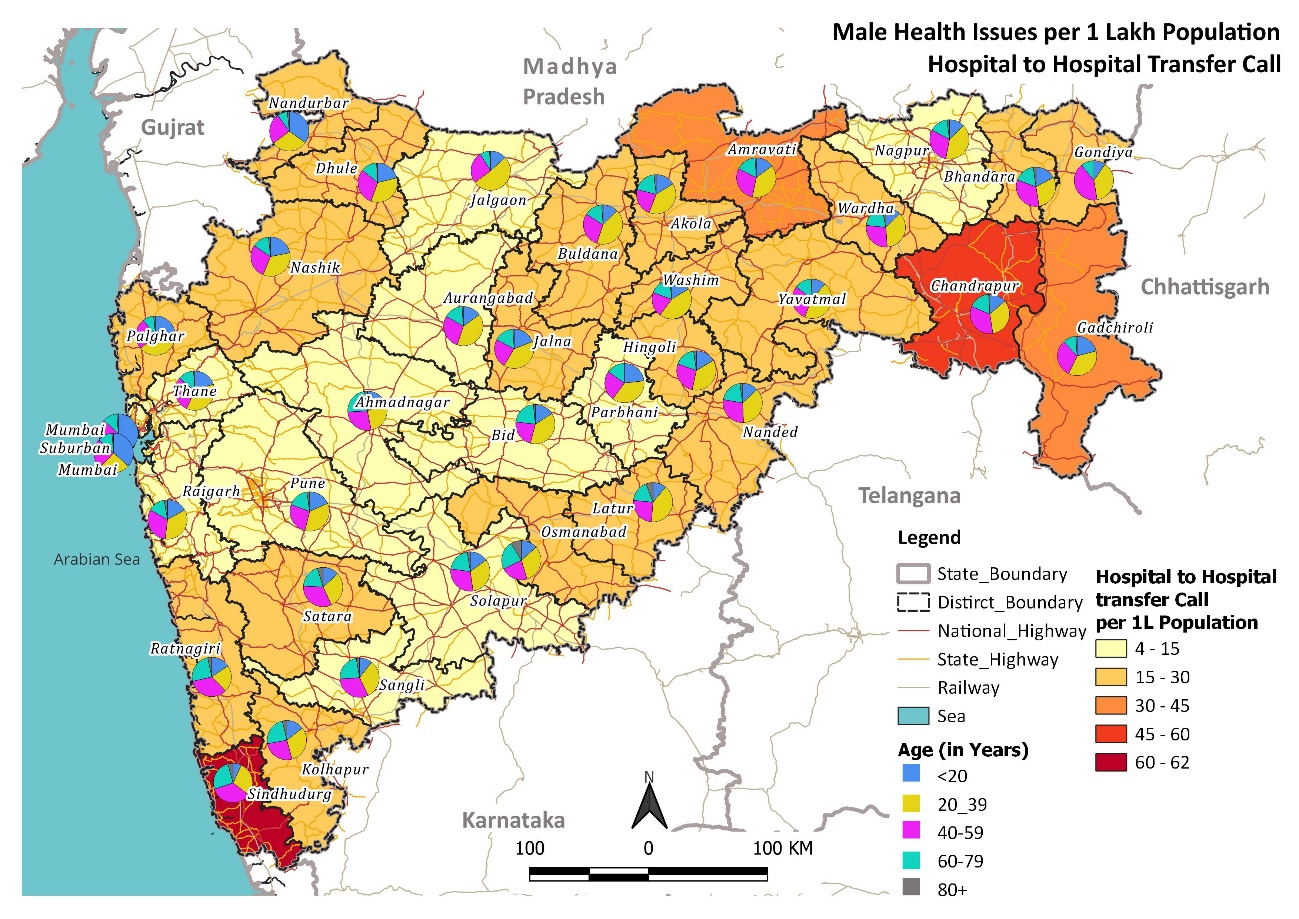


Appendix III: Figure 6: District-wise distribution of Male Health Issues per 1,00,000 population for Hospital-to-Hospital Transfer Calls. QGIS Geographic Information System v3.28.3-Firenze. QGIS.org, 2023. QGIS Association. http://www.qgis.org.


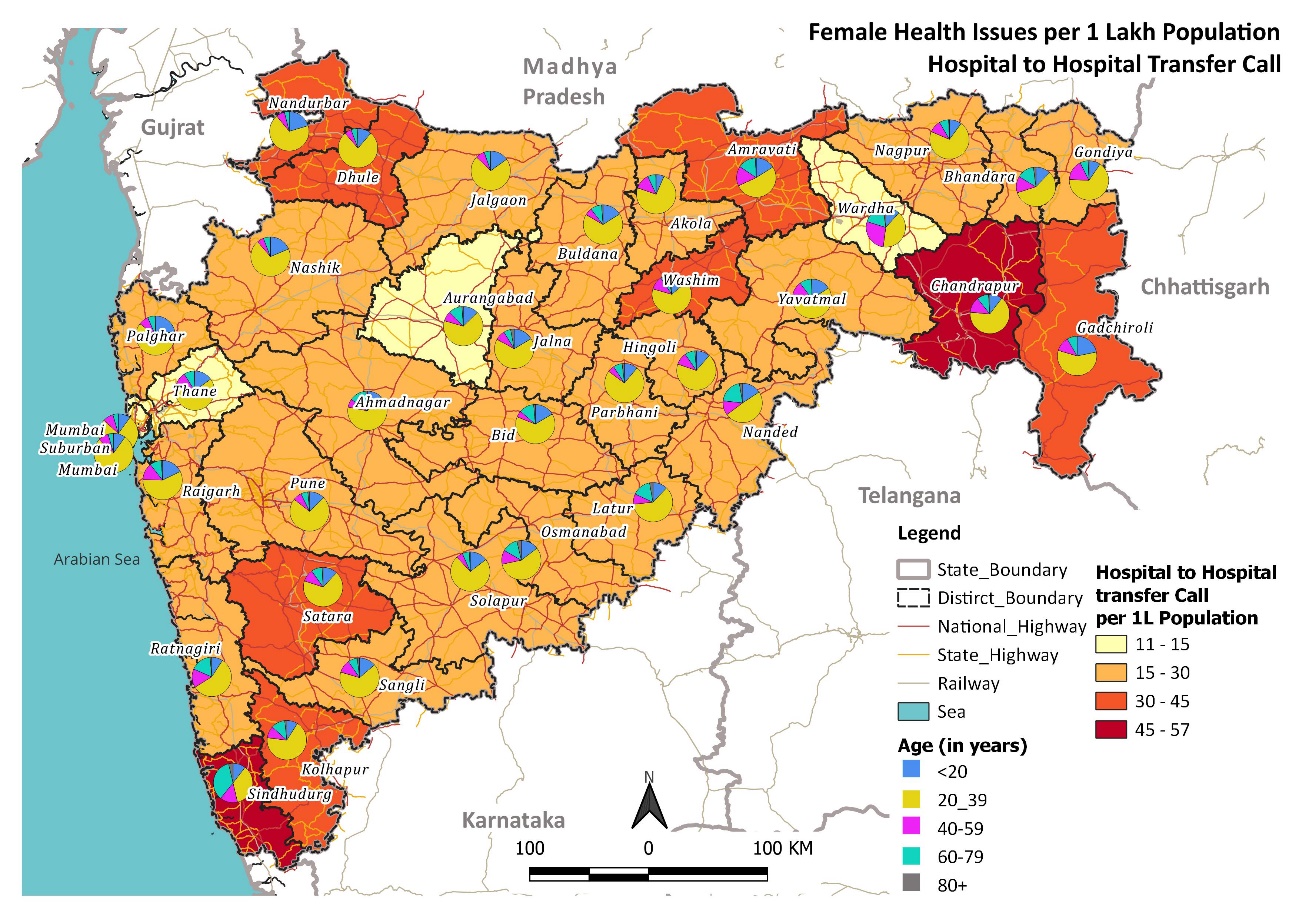


Appendix III: Figure 7: District-wise distribution of female Health Issues per 1,00,000 population for Hospital-to-Hospital Transfer Calls. QGIS Geographic Information System v3.28.3-Firenze. QGIS.org, 2023. QGIS Association. http://www.qgis.org.

**District-wise distribution of Disease Prominency Index for males and females per 1,00,000 Population for Hospital-to-Hospital Transfer Calls**


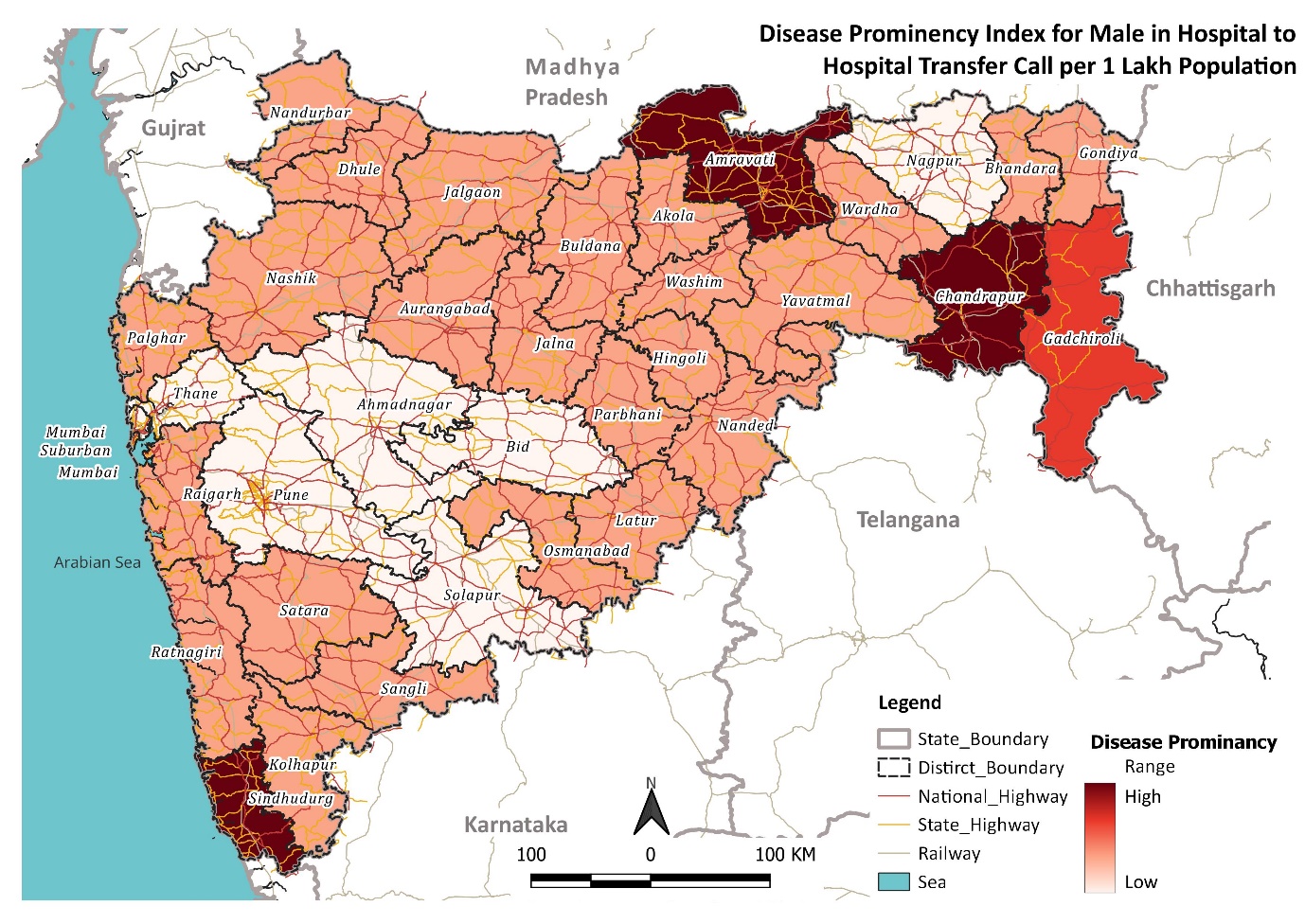


Appendix III: Figure 8: District-wise distribution of Disease Prominency Index for males per 1,00,000 Population for Hospital-to-Hospital Transfer Calls. QGIS Geographic Information System v3.28.3-Firenze. QGIS.org, 2023. QGIS Association. http://www.qgis.org.


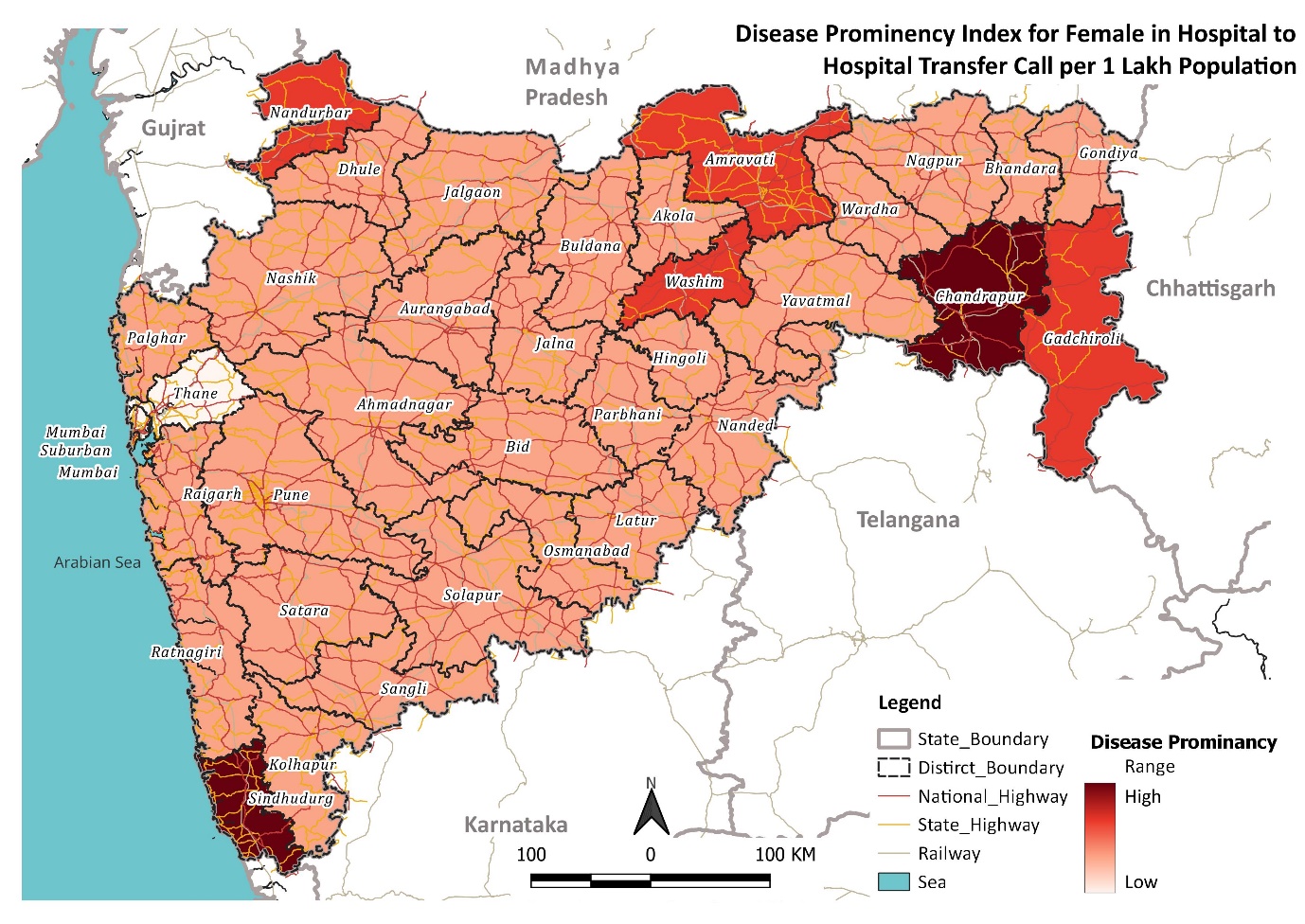


Appendix III: Figure 9: District-wise distribution of Disease Prominency Index for females per 1,00,000 Population for Hospital-to-Hospital Transfer Calls. QGIS Geographic Information System v3.28.3-Firenze. QGIS.org, 2023. QGIS Association. http://www.qgis.org.
