# Supplementary material for: Examining district-level disparity and determinants of timeliness of emergency medical services in Maharashtra, India
Source: Sci Rep. 2023 Dec 1;13:21239. doi: 10.1038/s41598-023-48713-1 (PMC10692338; doi:10.1038/s41598-023-48713-1)
Supplement: Supplementary file 4 — Supplementary Information 4. [file 41598_2023_48713_MOESM4_ESM.docx]

**Appendix IV**

Appendix IV: Table 1: Hourly variation of emergency and hospital to hospital transfer calls (Total Calls) in Maharashtra EMS – November 2022

| Time | Emergency Call (Total) | Hospital to Hospital Transfer Call (Total) |
| --- | --- | --- |
| 12 AM-1 AM | 482 | 614 |
| 1 AM-2 AM | 397 | 415 |
| 2 AM-3 AM | 389 | 340 |
| 3 AM-4 AM | 370 | 265 |
| 4 AM-5 AM | 325 | 246 |
| 5 AM-6 AM | 333 | 217 |
| 6 AM-7 AM | 390 | 235 |
| 7 AM-8 AM | 579 | 318 |
| 8 AM-9 AM | 751 | 615 |
| 9 AM-10 AM | 1003 | 1073 |
| 10 AM-11 AM | 1085 | 1561 |
| 11 AM-12 PM | 1125 | 1921 |
| 12 PM-1 PM | 905 | 1882 |
| 1 PM-2 PM | 808 | 1580 |
| 2 PM-3 PM | 712 | 1427 |
| 3 PM-4 PM | 739 | 1390 |
| 4 PM-5 PM | 685 | 1235 |
| 5 PM-6 PM | 799 | 1032 |
| 6 PM-7 PM | 794 | 1037 |
| 7 PM-8 PM | 803 | 1058 |
| 8 PM-9 PM | 795 | 1136 |
| 9 PM-10 PM | 750 | 1147 |
| 10 PM-11 PM | 663 | 1083 |
| 11 PM-12 AM | 515 | 799 |
|  | 16197 | 22626 |

Appendix IV: Figure 1: Hourly variation of emergency and hospital to hospital transfer calls (Total Calls) in Maharashtra EMS – November 2022 (Authors’ computation).

Appendix IV: Table 2: Hourly variation of Pregnancy related emergency and hospital to hospital transfer calls in Maharashtra EMS – November 2022

| Time | Total Calls | Emergency Calls | Hospital to Hospital Calls |
| --- | --- | --- | --- |
| 12AM-1AM | 421 | 185 | 236 |
| 1AM-2AM | 348 | 175 | 173 |
| 2AM-3AM | 322 | 179 | 143 |
| 3AM-4AM | 303 | 179 | 124 |
| 4AM-5AM | 302 | 181 | 121 |
| 5AM-6AM | 295 | 184 | 111 |
| 6AM-7AM | 325 | 196 | 129 |
| 7AM-8AM | 386 | 259 | 127 |
| 8AM-9AM | 479 | 270 | 209 |
| 9AM-10AM | 651 | 335 | 316 |
| 10AM-11AM | 813 | 339 | 474 |
| 11AM-12PM | 946 | 319 | 627 |
| 12PM-1PM | 871 | 287 | 584 |
| 1PM-2PM | 673 | 243 | 430 |
| 2PM-3PM | 613 | 194 | 419 |
| 3PM-4PM | 612 | 197 | 415 |
| 4PM-5PM | 570 | 191 | 379 |
| 5PM-6PM | 529 | 216 | 313 |
| 6PM-7PM | 457 | 191 | 266 |
| 7PM-8PM | 502 | 250 | 252 |
| 8PM-9PM | 533 | 254 | 279 |
| 9PM-10PM | 618 | 272 | 346 |
| 10PM-11PM | 576 | 249 | 327 |
| 11PM-12AM | 482 | 173 | 309 |
| Grand Total | **12627** | **5518** | **7109** |

Appendix IV: Figure 2: Hourly variation of Pregnancy related emergency and hospital to hospital transfer calls in Maharashtra EMS – November 2022 (Authors computation).
