# Supplementary material for: Examining district-level disparity and determinants of timeliness of emergency medical services in Maharashtra, India
Source: Sci Rep. 2023 Dec 1;13:21239. doi: 10.1038/s41598-023-48713-1 (PMC10692338; doi:10.1038/s41598-023-48713-1)
Supplement: Supplementary file 5 — Supplementary Information 5. [file 41598_2023_48713_MOESM5_ESM.docx]

Supplement 1 Extended Methods section (Qualitative Interview)

This paper is a part of a research project regarding analyzing the current MEMS service in Maharashtra including both quantitative and qualitative analyses. The qualitative analysis included semi-structured interviews with medical officers at MEMS ERC, and officials from the Dept. of Health, Govt. of Maharashtra. Here, the authors have reported only one of the findings of the interviews that focused on major socio-economic and socio-cultural oriented determinant of ambulance response time, particularly the patient assessment time at the scene and patient handover time at hospitals (other findings will be published elsewhere). The aim of the qualitative study here was to validate the findings from the quantitative analyses.

Study documents were reviewed by the Institutional Review Board (IRB) of Indian Institute of Technology Bombay. The committee approved the ethical conduct of the study and did not forward the same for Institute Ethics Clearance (IEC). IRB (IITB) issued a declaration of no objection (IITB-IRB/2022/049) confirming that the project fulfills the scientific and ethical standards for research. Written informed consent was obtained prior to data collection. An information sheet including brief introduction of the nature of the project was provided to the interviewees.

Research Team

Interviews were conducted by A.S, researcher, and V.P, a PhD student. The researchers have background in public health ethics and experience in qualitative research. One of the interview partners, who also co-supervises the project, is a co-author of the paper. No prior relationship existed between the study participants and interviewer. This study seeks to gain a better understanding of the determinants of ambulance response time, particularly the patient assessment time at the scene and patient handover time at hospitals. The analysis does not pretend to unveil the objective truth that slumbers in the data.

Study Design

Semi-structured interviews with medical officers from MEMS control centre and officials from the Dept. of Health, Govt. of Maharashtra working with 108 emergency service in Maharashtra were conducted. To align with the inquisitive approach of our qualitative research strategy, we utilized a combination of purposive sampling and snowball sampling techniques to enhance our ability to reach and engage with the participant group. We included medical officers from Pune MEMS control centre and officials from Department of Health, Maharashtra. All the participants were directly linked with MEMS service. Initial telephonic discussion followed by study invitation letters were sent to them as consent. Upon their agreeing, online meeting-based interview was conducted with officials from Dept of Health, whereas telephonic or in person meeting was scheduled with medical officers based on their preferences.

We interviewed 10 officials, including medical officers from MEMS Control Centre, Pune and officials from Dept of Health, Govt of Maharashtra. The rationale behind these numbers is rooted in their widespread acceptance as an appropriate size for qualitative research [1-3]. Interviews were conducted between January 2023 and February 2023, in Hindi and English. Only the interviewer and study participant were present during the interview. All interviews were recorded (in paper) and had a mean duration range of 26 minutes. The responses were transliterated verbatim and personal as well as local names were encrypted upon transcription.

Narrative-based interviewing was selected for its capability to explore current awareness, contextual interpretation of response time determinants, and real-life experiences. These interviews allowed for deeper investigation of socio-cultural and ethnic factors impacting response time. Semi-structured, open-ended, qualitative interviews were selected to cover the topic in a comparable, structured way. In the qualitative interviews, the authors intentionally embraced reduced standardization to yield more in-depth answers, thus affording a more comprehensive insight into the perspectives of medical officers.

Data analysis

Inductive thematic analysis was performed by V.P using the transcriptions using a qualitative data analysis software application. A coding system was developed by collaboratively comparing and discussing individually created codes, and coded segments. An iterative and interpretive process was employed to generate themes related to the subject of query (here, varying determinants of ambulance response time), and these themes were critically discussed with co-authors. This method facilitated a comprehensive understanding of the research topic.

References:

1. M Selveindran, S., Samarutilake, G. D., Rao, K. M. N., Pattisapu, J. V., Hill, C., Kolias, A. G., ... & Vijaya Sekhar, M. V. (2021). An exploratory qualitative study of the prevention of road traffic collisions and neurotrauma in India: perspectives from key informants in an Indian industrial city (Visakhapatnam). BMC public health, 21, 1-19.
2. Alawode, G. O., & Adewole, D. A. (2021). Assessment of the design and implementation challenges of the National Health Insurance Scheme in Nigeria: a qualitative study among sub-national level actors, healthcare and insurance providers. BMC Public Health, 21(1), 1-12.
3. Patel, A., Vissoci, J. R. N., Hocker, M., Molina, E., Gil, N. M., & Staton, C. (2017). Qualitative evaluation of trauma delays in road traffic injury patients in Maringá, Brazil. BMC health services research, 17(1), 1-8.
